# Effectiveness and mechanisms of interventions to reduce low-value thyroid function tests: a systematic review

**DOI:** 10.1186/s13643-026-03119-8

**Published:** 2026-02-25

**Authors:** Carolina Pioch, Meik Hildebrandt, Gregor Goetz, Verena Vogt

**Affiliations:** 1 Department of Health Care Management, Technical University of Berlin, Berlin, Germany; 2 Institute of General Practice and Family Medicine, Jena University Hospital, Friedrich Schiller University, Jena, Germany; 3 HTA Austria - Austrian Institute for Health Technology Assessment GmbH, Vienna, Austria

**Keywords:** Systematic review, Thyroid function tests, Low-value care, De-implementation, Clinical decision support systems

## Abstract

**Objective:**

Thyroid function tests are frequently overused. This systematic review aims to summarise the effectiveness of behaviour change interventions to reduce low-value thyroid testing and to identify theoretical foundations and contextual factors associated with their success.

**Design:**

We conducted a comprehensive search of Medline, Embase, Scopus, and the Cochrane Library for randomised and non-randomised controlled trials as well as before-and-after studies. We followed PRISMA guidelines, critically appraised study quality, and applied the GRADE approach to assess certainty of evidence. We categorised interventions as soft (education, reminders, feedback, guidelines) or structural (change in funding, clinical decision support systems).

**Results:**

We included 47 studies (54 interventions) including five randomised trials. Structural interventions, particularly clinical decision support systems, were the most common (*n* = 28). Most interventions reported a reduction in low-value thyroid testing (*n* = 52), with 40 of them having effects ≥ 20%. However, the certainty of evidence was very low to moderate. Among 49 interventions assessing volume reduction (test rates, expenditure), only two reported increased test rates. All 24 studies that measured improvement of care (appropriateness, shift in ordering pattern, coefficient of variation among physicians) indicated positive developments. Only four interventions referenced theoretical foundations or contextual factors.

**Conclusions:**

Structural interventions, especially clinical decision support systems, were most effective in reducing thyroid testing. While most interventions showed positive effects, the certainty of evidence remains limited, highlighting the need for more high-quality studies to support robust clinical practice changes. Our results may inform targeted interventions to reduce low-value thyroid testing at national, regional, and local levels.

**Supplementary Information:**

The online version contains supplementary material available at 10.1186/s13643-026-03119-8.

## Introduction

Thyroid function tests (TFTs) rank among the most frequently ordered laboratory tests worldwide [1], particularly thyroid-stimulating hormone (TSH) tests for diagnosing and managing thyroid disorders [2]. Alongside TSH, free hormone tests (free T3, T4) are often included in thyroid panels, further increasing the number of TFTs conducted [3]. For instance, approximately 10 million TFTs are performed annually in the UK at an estimated cost of £30 million [4, 5]. In Germany, about 30% of the adult population undergo thyroid function testing each year [6].

Despite the high volume of TFTs, the prevalence of thyroid dysfunction in Europe is only 3.8%, with 259.1 new cases per 100,000 people per year [7]. This discrepancy has raised concerns about the potential overdiagnosis and unnecessary treatment of conditions such as subclinical, asymptomatic hypothyroidism, which often resolves without intervention [8]. Unnecessary testing for suspected thyroid diseases appears to be widespread, frequently leading to additional and potentially avoidable medical procedures [9].

Clinical practice guidelines recommend against routine TSH screening for asymptomatic adults without known thyroid disease (consensus-based evidence, [10, 11]). Additionally, Free T3 or T4 tests are not advised for screening hypothyroidism unless pituitary or hypothalamic disease is suspected or known (consensus-based evidence, [12, 13]). Instead, TSH measurement is the most reliable method for detecting common forms of hypothyroidism and hyperthyroidism [14–16]. Despite the Choosing Wisely (CW) initiative’s aims to implement these guidelines, significant challenges remain in effectively translating them into practice.

Recent research identified a considerable amount of low-value care in German claims data, with 24.8% to 35.5% of TFTs deemed inappropriate [17, 18]. A bi-national study from Canada and the UK showed that approximately one-third of adult patients without a clear indication for testing underwent at least one TSH test within a 2-year period [19]. Similarly, a study from France suggests that inappropriate TFT ordering remains common [20]. TFTs therefore represent an emblematic example of low-value diagnostic testing, characterised by high baseline use, limited alignment with disease prevalence, and the potential to trigger downstream testing cascades. De-implementation of such unnecessary tests is frequently discussed in international literature [21]. Several systematic reviews have shown that targeted de-implementation strategies can effectively reduce low-value care [6]. Various interventions, including educational programmes for healthcare providers [22, 23], reminders [24, 25], decision support systems [26, 27], and stricter regulatory policies [28, 29], have been proposed to reduce unnecessary TFTs in different healthcare settings. These interventions can optimise clinical laboratory stewardship, contribute to cost savings, and improve healthcare resource allocation [3, 30].

Theoretical frameworks are essential for informing interventions aimed at de-implementing low-value care by identifying key elements that need to be addressed [31]. For instance, the Theoretical Domains Framework (TDF) explores barriers and facilitators for behaviour change [32], while the Choosing Wisely De-Implementation Framework systematically reduces low-value care [33]. A systematic review published by Zhelev et al. in 2016 provided an overview of behaviour change interventions to reduce the volume of TFTs ordered [34]. However, due to the studies’ poor methodological quality and reporting, strong conclusions could not be drawn, nor could specific intervention types be recommended. Furthermore, the theoretical foundations and contextual factors that contribute to the successful implementation of the interventions have not yet been thoroughly investigated. Since the publication of that review, a substantial number of new research related to interventions aimed at reducing the ordering of TFTs has emerged. In particular, as the latest studies included in the earlier review were published in 2014, before the widespread adoption of digital ordering systems and the increasing use of digital interventions, technological developments also need to be considered. In light of both the limitations of the earlier review and the growing body of relevant research, we are conducting a new systematic review, building on the work of Zhelev et al. [34].

Our review aims to identify effective strategies and their contexts by examining a series of interventions targeting the reduction of TFT orders. We address the following research questions (RQ):

1. What is the effectiveness of behaviour change interventions in reducing the ordering of TFTs?
2. Which theoretical foundations are used to explain the mechanisms underlying the interventions and which contextual factors are associated with the success of interventions aimed at improving evidence-based thyroid diagnostics?

In doing so, we build on the scope of the previous review: RQ1 was retained for continuity, while RQ2 was newly introduced to enhance the understanding of how and why interventions may work in different settings, drawing on information available in the included studies.

## Methods

We first manually searched for well-conducted systematic reviews on the same topic and identified one published in 2016 [34]. The review was assessed for methodological quality and risk of bias, showing moderate quality according to the AMSTAR 2 tool [35, 36] and a low risk of bias (RoB) evaluated by the ROBIS tool (RoB In systematic reviews, [37]). Assessments are available in Additional files 1 and 2.

Our review was guided by the Cochrane methodology and followed the Preferred Reporting Items for Systematic Reviews and Meta-Analyses (PRISMA) 2020 statement [38–40]. The PRISMA checklist is provided in Additional file 3.

### Registration

We prospectively registered the review in PROSPERO (CRD42023492441). Changes to the information provided at registration are reported in Additional file 4.

### Data sources, searches, and selection

We conducted a systematic literature search in line with the previous review by Zhelev et al. [34], using the same databases: MEDLINE (Ovid), Embase (Ovid), and the Cochrane Central Register of Controlled Trials (CENTRAL, the Cochrane Library). Searches were performed on the 21 st of November 2023. To increase comprehensiveness, we extended the methodology by also searching Scopus, screening the first 300 references from Google Scholar, hand-searching the reference lists of included articles, and contacting experts in the field to identify any additional relevant studies. We limited the search period for MEDLINE, Embase, and CENTRAL to articles published between the 1 st of January 2014 and the 21 st of November 2023, as articles published before 2014 were already screened for eligibility in the earlier review [34]. For Scopus and Google Scholar, no publication date limits were applied, as these databases were not included in the earlier review. We performed an update of the search on the 7th of July 2024.

We applied the same search strategy as Zhelev et al. [34], consisting of search terms related to (1) thyroid function tests and (2) inappropriate testing (Additional file 5). We included studies involving adults receiving TFTs in inpatient, outpatient, or emergency care settings. Eligible interventions comprised behaviour change interventions targeting physicians. Studies were required to compare these interventions to usual care and report outcomes related to test volume, appropriateness, costs, or patient health. We included randomised controlled trials (RCTs), non-randomised controlled studies, and before-and-after studies providing comparative data. We excluded studies that lacked a comparator, did not provide relevant outcome data, focused only on thyroid tests as part of a broader panel without disaggregated results, or were cross-sectional studies, opinion pieces, dissertations, or meeting abstracts. Only studies published in English or German were considered. All inclusion and exclusion criteria for assessing the effectiveness of the interventions (RQ1) followed those of the prior review and are outlined in Table 1.

**Table 1:** Inclusion and exclusion criteria of studies based on the PICO framework (Population, Intervention, Control, Outcome)

Attribute	Inclusion criteria	Exclusion criteria
Population	Adults receiving thyroid function tests	
Intervention	Behaviour change intervention types [41, 42]: Educational interventions Guideline and protocol development and implementation Changes to funding policy Reminders of existing guidelines and protocols Clinical decision support systems, including test request forms and computer-based decision support Audit and feedback	
Control	Usual care	
Outcome	Change in the total number of thyroid function tests Number of inappropriately ordered thyroid function tests Test-related expenditure Health benefits to individual patients	Studies encompassing ordered thyroid function tests along with other laboratory tests but reporting only the average effect (across all tests)
Study type	Randomised controlled trials Non-randomised controlled studies Before-and-after studies providing comparative data on at least one of the outcome measures	Cross-sectional studies Editorials and opinions Studies without comparative data Dissertations and meeting abstracts
Setting	Inpatient care, outpatient care, emergency department	
Language	English or German	All other languages

To address our additional RQ2, we supplemented the prior methodology by searching grey literature and conducting targeted searches for additional publications by the authors of the included interventions. This was done to determine whether potential contextual factors and theoretical backgrounds were described in the development and implementation of the interventions under review. We imported all search results into Endnote software and removed any duplicates before proceeding to the screening phase [43]. Based on the pre-defined eligibility criteria, two review authors (CP, MH) independently conducted title-abstract and full-text screening. Discrepancies were resolved by consensus and by consulting a third researcher (VV). No automation tools were used in the process.

### Data extraction and analysis

Data from the included studies were extracted by means of piloted data-extraction tables by two independent researchers (CP, MH). A list of all data items and detailed descriptions can be found in Additional file 6. Any discrepancies between the researchers were discussed until consensus was reached. For all identified RCTs, we contacted study authors via email in case of missing study protocols.

We used relative change (improvement/deterioration) as the standardised outcome metric, chosen due to the heterogeneity in outcomes and reporting. Following the approach outlined by Zhelev et al. [34], we set a threshold of ± 20% to interpret a relative change as large, indicating a substantial change in testing behaviour rather than minor variation. The outcomes included (1) changes in the total number of thyroid function tests, (2) test-related expenditure, (3) the number of inappropriately ordered thyroid function tests (appropriateness), (4) the pattern of ordering, and (5) the coefficient of variation (CoV) among physicians. We extracted the direction of the effect (positive/negative subject to the desired outcome measure). Confidence intervals and differences in means were extracted when reported. All calculated values are indicated as such.

We refrained from conducting a meta-analysis due to anticipated clinical heterogeneity among the studies. Specifically, we expected variability in intervention types, timing, and outcome measures, such as shifts towards TSH ordering, test volumes per population unit, or counts of laboratory tests per provider. Given these anticipated differences, a quantitative synthesis was not planned. Instead, we employed a narrative synthesis method. The analysis framework relied on an existing typology of behaviour change intervention types [41, 42], encompassing the following categories: (1) clinical decision support systems (CDSS), (2) changes to funding policy, (3) educational interventions, (4) reminders of existing guidelines and protocols, (5) guideline and protocol development and implementation, and (6) audit and feedback [34]. Furthermore, we introduced a grouping for the interventions and outcomes to support descriptive analysis and improve clarity of reporting (visualised in Additional file 7). We grouped the six types of interventions into *structural* and *soft interventions* based on their influence on physician behaviour. While we were guided by terminologies used in previous literature (active and soft [44], strict and soft [45], structural [46]), we developed the final categorisation to best reflect the characteristics of the interventions identified. The five outcome measures were divided into two categories: *volume reduction* (test rates, expenditure) and *improvement of care* (appropriateness, pattern, CoV).

*Structural interventions* directly influence physicians at the point of care and are integrated into their routine workflow, making them difficult to bypass. These interventions include *changes in funding*, where modifications to financial structures or incentives directly affect decision-making, and *CDSS*, which are embedded tools that directly guide or restrict physicians' choices during patient care. CDSS include alerts, changes in the existing order form, cost displays and reflex testing, respectively, as automatic discharge of tests. *Soft interventions* are those that provide physicians with informational resources or guidance outside of the immediate care setting, i.e. *reminders, education, guidelines/protocols*, and *audit/feedback*. These interventions are less directly tied to the point of care and may require active engagement from physicians to be used effectively, such as through educational meetings or reminder messages. They may also include tools that are not necessarily part of the standard working routine, like memorandum pocket cards or guidelines.

For RQ2, we extracted information on theoretical foundations and contextual factors when explicitly reported in the included studies or related publications. However, reporting was sparse and inconsistent, leaving little scope for synthesis or categorisation. Therefore, findings are presented narratively, with reference to specific theoretical models or quality improvement frameworks where available.

### Bias assessment and certainty of evidence

We used the Cochrane RoB 2.0 tool (RoB 2) for (cluster) RCTs and the ROBINS-I tool (RoB In Non-randomised Studies—of Interventions) for non-randomised studies of interventions (NRSI [47, 48]). RoB figures were created for each outcome domain separately using the robvis application [49].

The interventions in our review were evaluated using the Grading of Recommendations, Assessment, Development, and Evaluations (GRADE) approach, following Murad et al.’s guide for complex interventions [50, 51]. We graded the evidence both by outcome—assessing the overall effectiveness of intervention bundles from a policymaker/payer perspective—and by grouped interventions and outcomes. This dual grading approach aimed to inform policymakers on the efficacy of intervention bundles and to guide practitioners on the most effective components for patient care. To minimise potential publication bias, we expanded the databases included in the initial review, performed a grey literature search, and looked into registered trials (EU Clinical Trials Register *clinicaltrialsregister.eu/ctr-search/search*, WHO *trialsearch.who.int/Default.aspx*). All assessments were performed independently by two reviewers (CP and MH). Any disagreements were resolved by consensus involving a third reviewer (GG).

## Results

### Study selection

**Fig. 1:**
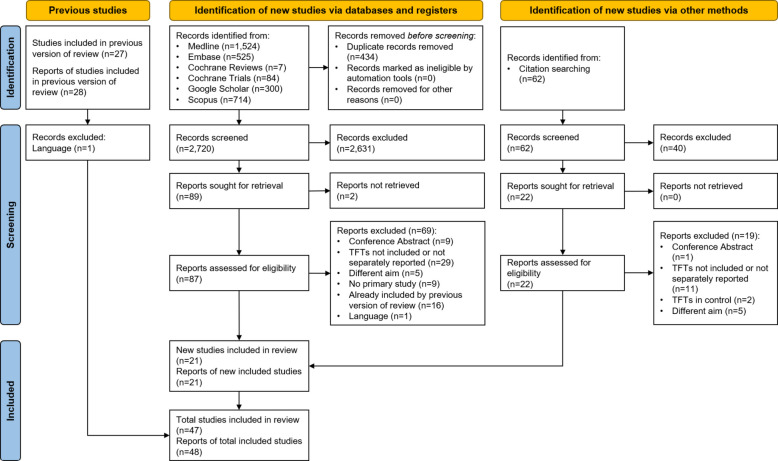
PRISMA flowchart of the systematic literature search. TFTs, thyroid function tests

From 2782 records screened, we identified 21 additional studies beyond those reported by Zhelev et al. [34], leading to a total of 47 unique studies included in this review. The updated search revealed no additional studies (213 new records screened). All identified studies were completed. Most of the identified studies used a before-and-after design (*n* = 34, including time series analysis), followed by nine non-randomised controlled studies and five (cluster) RCTs, two of which had a registration record. We contacted the authors of the remaining three studies and received a response from one of them. The study selection process is presented in Fig. 1. A list of all articles excluded after full-text screening and reasons for exclusion can be found in Additional file 8.

### Study characteristics

Most studies were conducted in the USA (*n* = 16), Canada (*n* = 8), and the UK (*n* = 6). They were published between 1979 and 2022. The majority of the studies were conducted in outpatient (*n* = 21) and inpatient care (*n* = 14). The remainder were conducted in both or in emergency departments (*n* = 12, Tables 2 and 3). Most of the studies were performed at a single medical site (*n* = 29, Table 2).

**Table 2:** Characteristics of the included studies

Study and country	Year	Study design	Setting	Target tests	Thyroid tests
**Studies identified in present review (*n* = 21)**					
Bateman et al., Canada [52]	2019	Before and after, single site	Inpatient rehabilitation centre	TSH + Vitamin D	TSH
Bejjanki et al., USA [53]	2018	Before and after, single site	Academic medical centre	17 laboratory tests	TSH, FT4
Bellodi et al., Italy [54]	2017	Controlled study, multiple sites	3 hospitals (10 wards)	8 laboratory tests	FT4
Bradshaw et al., USA [55]	2021	Before and after, single site	Academic Medical Centre	TFTs only	TSH, FT3, FT4
Caldarelli et al., Italy [56]	2017	Before and after, single site	Hospital (clinical laboratory)	TFTs only	TSH, FT3, FT4
Chami et al., Canada [45]	2021	Time series analysis with control group, multiple sites	Outpatient laboratories	8 laboratory tests	TSH
Dalal et al., USA [57]	2017	Before and after, single site	Urban teaching hospital	TFTs only	TSH, FT3, FT4
Delvaux et al., Belgium [58]	2020	Cluster RCT	72 primary care centres	17 laboratory tests	TSH
Elrewini et al., Saudi Arabia [59]	2022	Before and after, single site	Armed Forces Hospital	TFTs only	TSH
Gilmour et al., Canada [30]	2017	Before and after, single site	Academic ambulatory hospital	TFTs only	TSH, FT3, FT4
Janssens et al., Netherlands [44]	2015	Before and after, single site	General care and teaching hospital	82 laboratory tests	TSH, FT4
Krouss et al., USA [60]	2022	Interrupted time series, multiple sites	11 hospitals and over 70 ambulatory centres	TFTs only	T3, FT3
Leis et al., Canada [61]	2019	Before and after, single site	Coronary Care Unit (teaching hospital)	TFTs only	TSH
Leung et al., USA [62]	2017	Before and after, single site	Resident clinic in an outpatient clinic	70 laboratory tests	TSH, FT3, FT4
MacPherson et al., Australia [63]	2005	Before and after, single site	Pre-admission clinic	8 pathology tests + various investigations	TFTs (unspecified)
Muris et al., Netherlands [64]	2021	Before and after, multiple sites	57 general practices	22 laboratory tests	TSH, FT4
Notas et al., Greece [65]	2018	Before and after, single site	Tertiary teaching hospital	TFTs only	TSH, FT3, FT4, TGAb TPOAb
Salinas et al., Spain [66]	2016	Before and after, multiple sites	Public University Hospital + 9 primary care centres	13 laboratory tests	TSH, FT4
Sue et al., USA [67]	2019	Before and after, multiple sites	All outpatients within urban tertiary/quaternary care academic health system	TFTs only	T3
Taher et al., Canada [68]	2020	Before and after, single site	Tertiary hospital	TFTs only	FT3, FT4
Wintemute et al., Canada [69]	2019	Controlled study, multiple sites	6 family health teams	TFTs only	TSH
**Studies included in previous review (*n* = 27)**					
Adlan et al., UK [70]	2011	Before and after, single site	Medical Assessment Unit (hospital, acutely ill patients)	TFTs only	TFTs (TSH, FT4, TPOAb, TRAb)
Baker et al., UK [71]	2003	Cluster RCT	33 general practices	5 laboratory test groups	TFTs (TSH, FT4)
Berwick and Coltin, USA [23]	1986	Controlled cross-over, multiple sites	3 ambulatory centres (same health maintenance organization)	13 laboratory and imaging tests	T4
Chu et al., Australia [72]	2013	Before and after, single site	tertiary teaching hospital emergency department	23 laboratory tests	TFTs (unspecified)
Cipullo and Mostoufizadeh, USA [73]	1996	Before and after, single site	Community hospital	20 laboratory tests and preoperative testing (unspecified)	TFTs (T3RU)
Daucourt et al., France [25]	2000	Cluster RCT	General and psychiatric hospitals	TFTs only	TFTs (TSH, FT3, FT4, TRH)
Dowling et al., USA [74]	1989	Before and after, single site	Inner city community health centre	TSH + CBC	TSH
Emerson and Emerson, USA [26]	2001	Before and after, single site	University medical centre	All laboratory tests	TSH, T3, FT4, T4, FTI/T3RU
Feldkamp and Carey, USA [75]	1996	Before and after, single site	Metropolitan hospital and 22 satellite clinics	TFTs only	TSH, T3, T4, FTI/T3RU
Gama et al., UK [76]	1991	Controlled study, single site	District general hospital	5 laboratory test groups + others	TFTs (TSH, FT4)
Grivell et al., Australia [77]	1981	Before and after, single site	Tertiary-care community hospital	55 laboratory tests	T4
Hardwick et al., Canada [29]	1982	Before and after, multiple sites	Outpatient laboratories	TFTs only	T3, T4, ETR
Horn et al., USA [78]	2013	Interrupted time series with control group, multiple sites	Alliance of 5 multispecialty group practices	27 laboratory tests	TSH
Larsson et al., Sweden [22]	1999	Before and after, multiple sites	19 primary care centres	14 laboratory test groups	TSH, T3, FT4
Mindemark and Larsson, Sweden (follow up) [79]	2009	Before and after, multiple sites	16 primary healthcare centres	12 laboratory test groups	TSH, T3, T4, FT4
Nightingale et al., UK [80]	1994	Before and after, single site	Supra-regional liver unit (teaching hospital)	Various laboratory tests	TSH
Rhyne and Gehlbach, USA [81]	1979	Before and after, single site	Family Medicine group practice	TFTs only	TFTs (T3RU and T4)
Schectman et al., USA [82]	1991	Controlled study, single site	Primary care health maintenance organization practice	TFTs only	TFTs (TSH, T3-RIA, T3RU, T4)
Stuart et al., Australia [83]	2002	Before and after, single site	Public hospital emergency department	14 laboratory and 10 imaging tests	TFTs (unspecified)
Thomas et al., UK [24]	2006	Cluster RCT	85 primary-care practices	9 laboratory tests	TSH
Tierney et al., USA [27]	1988	RCT, single site	Academic internal medicine practice	8 laboratory tests	TSH
Tomlin et al., New Zealand [84]	2011	Controlled study, multiple sites	New Zealand primary care	8 laboratory tests	TSH, FT3, FT4
Toubert et al., France [85]	2000	Before and after, single site	Teaching hospital	TFTs only	TSH, FT3, FT4, TPOAb, TGAb, TRAb
Van Walraven et al., Canada [28]	1998	Interrupted time series, multiple sites	All clinical laboratories (not based in hospitals)	7 laboratory tests	TSH, T3RU, T4
Vidal-Trecan et al., France [86]	2003	Before and after, multiple sites	50 university hospitals	TFTs only	TSH, T3, FT3, T4, FT4
Willis and Datta, UK [87]	2013	Before and after, single site	Medical admissions unit in a district general hospital	3 laboratory test groups	TFTs (unspecified)
Wong et al., USA [88]	1983	Controlled study, single site	University teaching hospital	6 laboratory tests	TSH, T3RU,T3-RIA, T4-RIA

*CBC* complete blood count, *ETR* effective thyroxine ratio, *FT4* free thyroxine, *FT3* free triiodothyronine, *FTI* free T4 index or free thyroxine index, *RCT* randomised controlled trial, *RIA* radioimmunoassay, *TFTs* thyroid function tests, *TGAb* thyroglobulin antibodies, *TPOAb* thyroid peroxidase antibodies, *TSH* thyroid stimulating hormone (thyrotropin), *T3* triiodothyronine, *T3RU* triiodothyronine resin uptake, *TRH* TSH-releasing hormone, *TRAb* TSH-receptor antibodies

**Table 3:** Interventions of the included studies

Study and country	Setting	Soft interventions				Structural interventions		
		**Audit and feedback**	**Educational programmes**	**Guidelines and protocols**	**Reminders**	**Changes to funding**	**CDSS**	**Description of decision tool**
**Studies identified in present review (*n* = 21)**								
Bateman et al., Canada [52]	Inpatient	X	X					-
Bejjanki et al., USA [53]	Inpatient						X	Alert
Bellodi et al., Italy [54]	Inpatient						X	Alert
Bradshaw et al., USA [55]	Inpatient + ED		X	X			X	Alert
Caldarelli et al., Italy [56]	Outpatient						X	Reflex/Discharge
Chami et al., Canada [45]	Outpatient						X	Change of order form
Dalal et al., USA [57]	Inpatient						X	Reflex/Discharge
Delvaux et al., Belgium [58]	Outpatient						X	Alert
Elrewini et al., Saudi Arabia [59]	Unspecified		X	X			X	Alert
Gilmour et al., Canada [30]	Outpatient		X				X	Reflex/Discharge
Janssens et al., Netherlands [44]	Inpatient + outpatient			X				-
Krouss et al., USA [60]	Inpatient + outpatient						X	Alert
Leis et al., Canada [61]	Inpatient						X	Change of order form
Leung et al., USA [62]	Inpatient		X		X			-
MacPherson et al., Australia [63]	Inpatient			X			X	Change of order form
Muris et al., Netherlands [64]	Outpatient						X	Cost display
Notas et al., Greece [65]	Inpatient + outpatient						X	Reflex/Automatic discharge
Salinas et al., Spain [66]	Inpatient + outpatient						X	Reflex/Automatic discharge
Sue et al., USA [67]	Outpatient						X	Alert
Taher et al., Canada [68]	Inpatient + outpatient						X	Change of order form + Reflex/Automatic discharge
Wintemute et al., Canada [69]	Outpatient	X		X	X			-
**Studies included in previous review (*n* = 27)**								
Adlan et al., UK [70]	Inpatient			X				-
Baker et al., UK [71]	Outpatient	X		X				-
Berwick and Coltin, USA PCF$ [23]	Outpatient	X						-
Berwick and Coltin, USA PCFY [23]	Outpatient	X						-
Berwick and Coltin, USA TSE [23]	Outpatient		X					-
Chu et al., Australia [72]	ED						X	Change of order form
Cipullo and Mostoufizadeh, USA [73]	Inpatient			X				-
Daucourt et al., France MPC [25]	Inpatient				X			-
Daucourt et al., France TRF [25]	Inpatient						X	Change of order form
Daucourt et al., France Both [25]	Inpatient				X		X	Change of order form
Dowling et al., USA [74]	Outpatient	X	X					-
Emerson and Emerson, USA [26]	Outpatient						X	Change of order form
Feldkamp and Carey, USA [75]	Inpatient + outpatient						X	Reflex/Automatic discharge
Gama et al., UK [76]	Inpatient + outpatient	X						-
Grivell et al., Australia [77]	Inpatient	X						-
Hardwick et al., Canada [29]	Outpatient			X		X		-
Horn et al., USA [78]	Outpatient						X	Cost display
Larsson et al., Sweden [22]	Outpatient		X					-
Mindemark and Larsson, Sweden (follow up) [79]	Outpatient		X					-
Nightingale et al., UK [80]	Inpatient	X		X			X	Change of order form
Rhyne and Gehlbach, USA [81]	Outpatient		X	X				-
Schectman et al., USA Reminder + Feedback [82]	Outpatient	X	X		X			-
Schectman et al., USA Reminder [82]	Outpatient		X		X			-
Stuart et al., Australia [83]	ED	X	X	X				-
Thomas et al., UK Feedback [24]	Outpatient	X						-
Thomas et al., UK Reminder [24]	Outpatient				X			-
Thomas et al., UK Both [24]	Outpatient	X			X			-
Tierney et al., USA [27]	Outpatient						X	Alert
Tomlin et al., New Zealand [84]	Outpatient	X	X	X				-
Toubert et al., France [85]	Inpatient + outpatient			X	X			-
Van Walraven et al., Canada [28]	Outpatient			X		X	X	Change of order form
Vidal-Trecan et al., France [86]	Inpatient + outpatient		X	X			X	Change of order form
Willis and Datta, UK [87]	Inpatient		X	X				-
Wong et al., USA [88]	Inpatient			X			X	Change of order form

Multiple interventions per study (indicated in study column) or multiple types of intervention per intervention (multi-component interventions) possible. Alerts including best practice pop-ups, test suggestions or information (e.g. test rates, disease probabilities)

*CDSS* clinical decision support system, *ED* emergency department, *MPC* memorandum pocket card, *TRF* test request form, *TSE* test-specific education, *PCFY* peer comparison feedback on yield of tests, *PCF$* peer comparison feedback on cost of test use

The average total observation period of the studies was 25 months, with an average of 12-month preintervention phase and a 14-month intervention/postintervention period (additional information on study characteristics is provided in Additional file 9). In total, 19 studies targeted TFTs only, while the remaining 28 studies aimed to reduce a broader number of laboratory and imaging tests. A total of 54 distinct interventions were performed within the 47 studies (Table 2).

While the previous review identified soft interventions (education, guidelines/protocols, reminders, and audit/feedback) as the most common types [34], our review revealed that most of the studies introduced structural interventions (CDSS and changes in funding, *n* = 30). Of these, only two studies (no newly identified studies) assessed changes to funding (Table 3). Therefore, CDSS is the most widely used type (*n* = 28), with 17 of the 21 newly identified studies employing CDSS. The most common CDSS involved changes of the order form (*n* = 12), alerts (*n* = 8), or reflex with automatic discharge (*n* = 7). Alerts included information about best practice, suggestions for testing and discharge, or probabilities of thyroid disease. Additionally, two studies displayed the costs of the tests being ordered. Among the soft interventions, guidelines/protocols (*n* = 18) and education (*n* = 16) were most commonly employed, followed by audit/feedback (*n* = 14) and reminders (*n* = 9). In total, 19 interventions consisted solely of soft components, 25 involved structural changes, and ten combined both components (Table 3).

### Bias assessment

The majority of the studies did not have a control group (*n* = 33). Following an approach outlined by HTACG, we refrained from conducting a formal RoB assessment for these studies, as their inherent lack of internal validity is unlikely to be changed by a RoB assessment [89].

Two RCTs were of low RoB; the remaining three had some concerns due to potential selection bias. Primarily due to the risk of confounding, the RoB in controlled studies (*n* = 9) was *critical* in three studies (*n* = 4 outcomes), *serious* in six studies (*n* = 8 outcomes), and *moderate* in one study. The outcomes within a study showed the same RoB for each domain due to the relation of the outcome measures. The full RoB assessment can be found in Additional file 10.

### Effectiveness of the interventions

Of the 54 interventions, the majority showed a positive direction of change (*n* = 52), a considerable number had effects ≥ 20% (*n* = 40), and many reported significant changes (*n* = 29, with 19 not reported; Table 4) (RQ1). Further synthesis was impracticable due to the heterogeneity of measures, tests, and reporting standards. Only 12 interventions reported confidence intervals, while the difference in means was even less common (*n* = 7). Relative change ranged from a 32% decrease to a 172% improvement (additional information on study results and reported effect measures is provided in Additional file 11). We refrained from computing means due to the variability of measures within the outcomes. The most frequently assessed outcome was the number or rates of tests (*n* = 43), with 41 showing a positive effect. A large proportion of these had effects of ≥ 20% (*n* = 30). Significant changes were reported in 22 of the 43 interventions, while 17 did not report significance. Expenditure outcomes were less common (*n* = 9), but all showed positive changes, with significant changes reported in two interventions (six not reported). Relative improvements ≥ 20% were shown by five interventions assessing the expenditure. A total of 49 interventions assessed volume-related outcomes (test numbers/rates, expenditure; Table 4).

**Table 4:** Results of the interventions

Study and country	Type of Study	Intervention	Direction of effect	Relative change $$\ge 20\boldsymbol{\%}$$	Significant effect	Notes on outcome measure
**Appropriateness**						
Daucourt et al., France MPC [25]	RCT	Reminder + Decision tool	** + **	−*	**−**	Proportion of TFTs ordered in accordance with guidelines
Daucourt et al., France TRF [25]	RCT	Reminder + Decision tool	** + **	+ *	** + **	See above
Daucourt et al., France Both [25]	RCT	Reminder + Decision tool	** + **	+ *	**NR**	See above
Delvaux et al., Belgium [58]	RCT	Decision tool	** + **	**−**	** + **	Appropriate tests/total orders
Schectman et al., USA Reminder + Feedback [82]	Controlled	Reminder + Feedback	** + **	+ *	**NR**	Compliance rate with TFT protocol
Schectman et al., USA Reminder [82]	Controlled	Reminder	** + **	+ *	** + **	See above
Caldarelli et al., Italy [56]	Uncontrolled	Decision tool	** + **	−*	**NR**	Prescriptive appropriateness through the ratios TSH/FT4, TSH/FT3 and the ratio “TSH Reflex”/TSH
Dowling et al., USA [74]	Uncontrolled	Education + Feedback	** + **	+ *	**−**	Indicated TSH/visit
Elrewini et al., Saudi Arabia [59]	Uncontrolled	Education + Guidelines	** + **	**−**	** + **	Unnecessary requests/total TSH requests
Feldkamp and Carey, USA [75]	Uncontrolled	Decision tool	** + **	+ *	**NR**	Shift towards TSH
Leis et al., Canada [61]	Uncontrolled	Decision tool	** + **	+ *	** + **	Proportion of not indicated physician TSH orders
Nightingale et al., UK [80]	Uncontrolled	Guidelines + Decision tool + Feedback	** + **	+ *	**NR**	Patients requiring an investigation/patients tested
Rhyne and Gehlbach, USA [81]	Uncontrolled	Education + Guidelines	** + **	+ *	**−**	High/low indication test proportion
Toubert et al., France [85]	Uncontrolled	Guidelines + Reminders	** + **	+ *	** + **	Frequency of appropriate use of thyroid function tests
**Coefficient of variation (CoV)**						
Berwick and Coltin, USA PCF$ [23]	Controlled	Feedback	** + **	** + **	**NR**	CoV of rate of test use among physicians within centres
Berwick and Coltin, USA PCFY [23]	Controlled	Feedback	** + **	** + **	**NR**	See above
Berwick and Coltin, USA TSE [23]	Controlled	Education	** + **	**−**	**NR**	See above
**Expenditure**						
Tierney et al., USA [27]	RCT	Decision tool	** + **	**−**	**−**	Charges per visit
Tomlin et al., New Zealand [84]	Controlled	Education + Guidelines + Feedback	** + **	**−**	**NR**	
Bejjanki et al., USA [53]	Uncontrolled	Decision tool	** + **	+ *	**NR**	Cost savings from reducing duplicates
Caldarelli et al., Italy [56]	Uncontrolled	Decision tool	** + **	−*	**NR**	
Elrewini et al., Saudi Arabia [59]	Uncontrolled	Education + Guidelines	** + **	+ *	**NR**	Cost spent on the unnecessary requests of TSH tests
Hardwick et al., Canada [29]	Uncontrolled	Guidelines + Change in Funding	** + **	** + **	**NR**	Change in total expected costs
Janssens et al., Netherlands [44]	Uncontrolled	Guidelines	** + **	+ *	**NR**	
Leung et al., USA [62]	Uncontrolled	Education + Reminder	** + **	**NR**	** + **	Change of laboratory costs
Stuart et al., Australia [83]	Uncontrolled	Education + Guidelines + Feedback	** + **	** + **	** + **	Mean costs per patient
**Pattern**						
Tomlin et al., New Zealand [84]	Controlled	Education + Guidelines + Feedback	** + **	** + **	** + **	Shift towards TSH
Wong et al., USA [88]	Controlled	Guidelines + Decision tool	** + **	+ *	** + **	Sought to reduce complete thyroid panels
Emerson and Emerson, USA [26]	Uncontrolled	Decision tool	** + **	+ *	** + **	Shift towards FT4 and thyroid cascade
Hardwick et al., Canada [29]	Uncontrolled	Guidelines + Change in Funding	** + **	+ *	**NR**	Sought to reduce proportion of T3 tests
Larsson et al., Sweden [22]	Uncontrolled	Education	** + **	−*	**TSH/TFTs: + ** **T3/TSH: + ** **T4/TSH: −**	Shift towards TSH (primary care centres)
Larsson et al., Sweden [22]	Uncontrolled	Education	** + **	+ *	**NR**	Shift towards TSH (individual physicians)
Mindemark and Larsson, Sweden (follow up) [79]	Uncontrolled	Education	** + **	T3/TSH + * **T4 + FT4/** TSH −*	**−**	Median of physicians; shift towards TSH
Toubert et al., France [85]	Uncontrolled	Guidelines + Reminders	** + **	+ *	**NR**	Shift towards TSH
Van Walraven et al., Canada [28]	Uncontrolled	Guidelines + Change in Funding + Decision tool	** + **	** + **	** + **	Shift towards TSH
Vidal-Trecan et al., France [86]	Uncontrolled	Education + Guidelines + Reminders + Decision tool	** + **	**−**	**NR**	Shift towards TSH
**Test numbers or rates**						
Baker et al., UK [71]	RCT	Guidelines + Feedback	** + **	−*	**−**	Tests per 1,000 patients
Thomas et al., UK Feedback [24]	RCT	Feedback	** + **	−*	** + **	Tests per 10,000 patients
Thomas et al., UK Reminder [24]	RCT	Reminder	** + **	−*	** + **	See above
Thomas et al., UK Both [24]	RCT	Reminder + Feedback	** + **	**NR**	**NR**	See above
Bellodi et al., Italy [54]	Controlled	Decision tool	** + **	**Delta: −** **Cento: + ** **Ferrara: −**	**NR**	Number of laboratory tests requested by wards
Berwick and Coltin, USA PCF$ [23]	Controlled	Feedback	** + **	**−**	**NR**	Tests per 1,000 encounters per physician
Berwick and Coltin, USA PCFY [23]	Controlled	Feedback	**−**	** + **	**NR**	See above
Berwick and Coltin, USA TSE [23]	Controlled	Education	** + **	**−**	**NR**	See above
Chami et al., Canada [45]	Controlled	Decision tool	** + **	**−**	**−**	Number of thyroid tests
Gama et al., UK [76]	Controlled	Feedback	** + **	** + **	**I: + ** **C: −**	Tests per outpatient visit
Horn et al., USA [78]	Controlled	Decision tool	** + **	**NA**	**−**	Monthly orders per 1,000 patients
Schectman et al., USA [82]	Controlled	Reminder + Feedback	** + **	−*	** + **	Number of TFTs per patient; Feedback and Non-Feedback group combined
Tomlin et al., New Zealand [84]	Controlled	Education + Guidelines + Feedback	** + **	**TSH: −** **FT3/FT4: + **	** + **	Tests per year per GP
Wintemute et al., Canada [69]	Controlled	Guidelines + Feedback	** + **	**−**	** + **	
Wong et al., USA [88]	Controlled	Guidelines + Decision tool	** + **	**TSH and T3RIA: + ** **T3RU and T4RIA: −**	**NR**	Tests per month
Adlan et al., UK [70]	Uncontrolled	Guidelines	** + **	+ *	** + **	Proportion of admitted patients offered TFTs
Bateman et al., Canada [52]	Uncontrolled	Education + Feedback	** + **	** + **	**NR**	Proportion of admitted patients offered TFTs
Bejjanki et al., USA [53]	Uncontrolled	Decision tool	** + **	**FT4: −** **TSH: + **	**FT4: −** **TSH: + **	Percentage change in the number of inpatient duplicate orders
Bejjanki et al., USA [53]	Uncontrolled	Decision tool	** + **	**FT4: −** **TSH: + **	**FT4: −** **TSH: + **	Odds of percentage duplicate
Bradshaw et al., USA [55]	Uncontrolled	Decision tool	** + **	** + **	**TSH: −** **FT4: + **	Number of inappropriate TSH tests ordered; FT3 excluded due to low baseline numbers
Caldarelli et al., Italy [56]	Uncontrolled	Decision tool	** + **	**TSH: −** **FT4: −** **FT3: + **	**NR**	Number of thyroid tests
Chu et al., Australia [72]	Uncontrolled	Decision tool	** + **	+ *	** + **	Number of tests ordered per 100 ED presentations
Cipullo and Mostoufizadeh, USA [73]	Uncontrolled	Guidelines	** + **	**−**	**NR**	Tests/discharge
Dalal et al., USA [57]	Uncontrolled	Decision tool	** + **	** + **	** + **	Number of tests of fT3 and fT4 orders per total TSH orders
Dowling et al., USA [74]	Uncontrolled	Education + Feedback	** + **	+ *	**−**	Rates of ordering TSH tests per visit
Emerson and Emerson, USA [26]	Uncontrolled	Decision tool	** + **	+ *	** + **	Test sets ordered (significance for total TFTs)
Feldkamp and Carey, USA [75]	Uncontrolled	Decision tool	** + **	TSH: −* T4: + * T3RU: + *	**NR**	Tests per 1,000 patients (T3 not reported)
Gilmour et al., Canada [30]	Uncontrolled	Education + Decision tool	** + **	** + **	** + **	Median number of tests performed (FT3 and FT4; TSH used for initial appropriateness)
Grivell et al., Australia [77]	Uncontrolled	Feedback	**−**	+ *	**NR**	Tests per 1,000 patients
Hardwick et al., Canada [29]	Uncontrolled	Guidelines + Change in Funding	** + **	+ *	**NR**	
Janssens et al., Netherlands [44]	Uncontrolled	Guidelines	** + **	+ *	**NR**	
Krouss et al., USA [60]	Uncontrolled	Decision tool	** + **	+ *	** + **	Orders per 1,000 patient days (inpatient)/per 1,000 encounters (outpatient)
Leis et al., Canada [61]	Uncontrolled	Decision tool	** + **	+ *	** + **	Patients with any TSH assay request/patients with physician-signed order
MacPherson et al., Australia [63]	Uncontrolled	Guidelines + Decision tool	** + **	+ *	** + **	
Muris et al., Netherlands [64]	Uncontrolled	Decision tool	** + **	+ *	** + **	Mean test ordering rate per 1,000 patients per month per general practice
Notas et al., Greece [65]	Uncontrolled	Decision tool	** + **	** + **	** + **	Number of TFTs per TSH ordered (FT4 and FT3) and per cent patients with TFT order, inpatients
Notas et al., Greece [65]	Uncontrolled	Decision tool	** + **	+ *	**NR**	Number of TFTs per TSH ordered (FT4 and FT3), outpatients
Rhyne and Gehlbach, USA [81]	Uncontrolled	Education + Guidelines	** + **	−*	** + **	TFTs per 100 patients
Salinas et al., Spain [66]	Uncontrolled	Decision tool	** + **	+ *	**NR**	Ratio of FT4/TSH
Sue et al., USA [67]	Uncontrolled	Decision tool	** + **	** + **	** + **	T3 laboratory tests/10,000 patients per week
Taher et al., Canada [68]	Uncontrolled	Decision tool	** + **	** + **	**NR**	Total number of fT4 and fT3 tests per month
Toubert et al., France [85]	Uncontrolled	Guidelines + Reminders	** + **	+ *	**NR**	
Van Walraven et al., Canada [28]	Uncontrolled	Guidelines + Change in Funding + Decision tool	** + **	**TSH: −** **T4: + **	** + **	Tests per 100,000 patients per month; comparison with expected values (T3RU not reported)
Vidal-Trecan et al., France [86]	Uncontrolled	Education + Guidelines + Reminders + Decision tool	** + **	**−**	**NR**	
Willis and Datta, UK [87]	Uncontrolled	Education + Guidelines	** + **	+ *	** + **	Tests per admission

Interventions sorted by outcome and type of study. Effects based on numerical results that can be found in Additional file 11. Deviations from standard outcome measure listed in last column

^*^Based on authors’ calculations (for values pre/postintervention see Additional file 11)

*ED* emergency department, *FT4* free thyroxine, *FT3* free triiodothyronine, *GP* general practitioner, *MPC* memorandum pocket card, *NR* not reported, *TFTs* thyroid function tests, *TRF* test request form, *TSE* test-specific education, *TSH* thyroid stimulating hormone (thyrotropin), *T3* triiodothyronine, *T3RU* triiodothyronine resin uptake, *PCFY* peer comparison feedback on yield of tests, *PCF$* peer comparison feedback on cost of test use, *RCT* randomised controlled trial, *RIA* radioimmunoassay

Improvement-related outcomes (appropriateness, pattern, and CoV) were assessed in 24 interventions. Appropriateness was frequently studied (*n* = 14), with all showing positive direction, many with effects ≥ 20% (*n* = 10), and significant changes in six studies (five not reported). Pattern changes were less common (*n* = 8), but all showed positive effects, with significant changes in some (*n* = 4, with three not reported). Effects of ≥ 20% were shown in three interventions. CoV outcomes were least assessed (*n* = 3), with no significant changes reported (two interventions showed relative improvements ≥ 20%). Over all outcomes and interventions, the results of structural interventions were slightly more positive, with 100% showing positive effects (61% significant) and 74% with large effect sizes, compared to combined and soft interventions (combined 100% positive (40% significant), 60% large effect; soft, 94% positive (44% significant), 47% large effects; Additional file 11).

To contextualise these findings, we next evaluated the certainty of evidence using GRADE. For structural interventions (CDSS, changes in funding), we found a significantly positive effect on the outcomes that measure *improvement of care* based on two cluster RCTs (*n* = 1 effect ≥ 20%, high certainty of evidence (CoE)). Four uncontrolled studies supported these findings (positive direction, *n* = 3 effects ≥ 20%, two significant). For *volume reduction*, one RCT indicated a trend towards reducing test rates (positive, not significant (NS), low CoE), with 16 non-randomised studies pointing in the same direction (positive direction, *n* = 12 effects ≥ 20%, *n* = 9 significant). For soft interventions, one cluster RCT indicated positive *improvement of care* (NS, moderate CoE), as well as ten non-randomised interventions (*n* = 9 effects ≥ 20%, four significant). Two cluster RCTs featuring four soft interventions indicated they could achieve *volume reduction* (two significant, moderate CoE). Of 19 non-randomised interventions, 17 showed a positive direction as well (*n* = 9 effects ≥ 20%, nine significant). Similarly, combined interventions showed positive effects on the *improvement of care* based on one cluster RCT* (*positive, effect ≥ 20%, NS, moderate CoE) and six non-randomised interventions (*n* = 3 effects ≥ 20%, *n* = 3 significant). No RCT assessed *volume reduction*, but eight non-randomised interventions showed a positive trend (*n* = 5 effects ≥ 20%, *n* = 3 significant). The full GRADE evidence profiles can be found in Additional file 7, organised by intervention type (Table 7.1) and outcome category (Table 7.2).

Most of the studies had unreported funding (*n* = 21), some were non-profit (*n* = 14), lacked a specific grant (*n* = 12), or had unclear funding (*n* = 1). Twenty-two studies reported ethics committee approval (*n* = 18) or stated that approval was not required (*n* = 4). Twenty-five studies did not report on ethics approval (Additional file 12 includes information on ethics approval, funding, and conflict of interest). Although we cannot entirely rule out an overestimation of the predominantly positive findings, there is no indication of significant publication bias. No relevant trials were found in the extended registry search. All evidence of non-randomised trials was rated to be of very low certainty (Additional file 7).

### Theoretical foundations and contextual factors

Information on theoretical foundations and contextual factors provided by the included studies was sparse (RQ2). Four interventions reported on the theoretical foundations of their interventions, going further than conventional references to systematic reviews or guidelines (relevant text passages included in Additional file 9). We did not find additional literature reporting on theoretical foundations or contextual factors. Consideration of contextual factors beyond theoretical models was not explicitly mentioned in any included study.

Elrewini et al. (education + guidelines + retest alert) performed a root cause analysis to develop a corresponding action plan that implements the identified root causes [59]. Leis et al. (change of order form) used a simulated setting to assess unnecessary test ordering through a hypothetical patient scenario with a quasi-randomised sample of participants. They concluded that the presence of a checkbox influences ordering behaviour [61, 90]. Stuart et al. (feedback + education + guidelines) based the components of their intervention on the core elements of the PRECEDE framework (Predisposing, Reinforcing, and Enabling Causes in Educational Diagnosis and Evaluation, [41, 83]). Wintemute et al. (guidelines + feedback + reminder) based their choice of intervention on Rogers’ theory of diffusion of innovations, supplementing evidence-based recommendations with active reminders and local feedback ([69, 91], Additional file 9).

Additionally, three interventions performed Plan-Do-Study-Act (PDSA) cycles based on different approaches. Bateman et al. (feedback + education) applied a systematic approach using process measures and evaluation based on quality improvement literature [52, 92, 93]. Gilmour et al. (education + reflex testing) and Taher et al. (reflex testing) developed their interventions based on PDSA cycles using the model for improvement framework for continuous quality improvement by Provost et al. ( [30, 68, 94, 95], Additional file 9).

## Discussion

Our review sought to evaluate interventions aimed at reducing unnecessary TFTs by reviewing and synthesising recent studies, building on the review by Zhelev et al. from 2016 [34]. We identified 21 new studies, contributing to a total of 47 unique studies included in our review. The interventions comprised soft (education, guidelines/protocols, reminders, and audit/feedback) and structural (CDSS and changes in funding) interventions. The synthesis of 54 interventions across the included studies revealed predominantly positive outcomes, with 52 interventions associated with reductions or improvements in at least one outcome. Most studies reported relative reductions of at least 20%. Restricting the evidence to the five included (cluster) RCTs reaffirmed this pattern, as all five trials reported some degree of beneficial impact. In this review, we applied the Cochrane methodology and the GRADE approach, enhancing the methodological rigour [38, 50]. Nevertheless, the overall certainty of evidence was rated as low, indicating that the observed effects should be interpreted with caution and primarily viewed as indicative of promising trends rather than definitive evidence of effectiveness.

We observed a shift towards structural interventions, particularly CDSS. Of the 21 newly identified studies, 17 employed some form of CDSS, with alerts being the most commonly reported. The clustered GRADE assessment suggested potential effectiveness of de-implementation interventions, where structural interventions showed slightly more compelling results (RQ1). RCT evidence for structural interventions indicated improvement of care (moderate CoE, *n* = 2 RCTs) and volume reduction (low CoE, *n* = 1 RCT). Though RCT evidence for soft interventions shows similar results (volume reduction *n* = 2 RCTs, improvement of care *n* = 1 RCT, both moderate CoE), observational evidence suggests a higher success rate for structural interventions (structural interventions: 100% positive, 61% significant; soft interventions: 94% positive, 44% significant). The increased use of structural interventions aligns with the expectation that direct approaches at the point of care may be more likely to yield a reduction in test orders compared to soft interventions [45]. A systematic review performed by Cliff et al. on the effectiveness of CW interventions concluded that structural interventions are more effective than soft approaches [21]. Similarly, a CDSS was found to be the most effective intervention in de-implementing low-value cancer care [96]. Further research is needed to evaluate the effectiveness of structural interventions, under various conditions such as system usability, integration with existing practices, and user engagement, which are often underreported [97]. In particular, it remains unclear how differences in CDSS design, implementation context, and integration into clinical workflows shape both the magnitude and durability of observed effects.

Next to potentially promising structural interventions, our study also identified soft interventions that, while less impactful, showed compelling effects in some settings. These findings suggest that both structural and soft interventions may be suitable options for reducing TFTs. Similarly, research by Kobewka et al., which examined interventions aimed at reducing all sorts of laboratory test utilisation, found positive results across all types of interventions [98]. In particular, soft interventions can be considered in settings where profound structural interventions are not feasible. Regardless of the specific intervention type, a recent overview of reviews by Kien et al. indicates that de-implementation strategies are effective across various low-value services [6]. This overview complements our review and can inform decision-makers about the range of interventions available for reducing low-value care, as well as the potential for de-implementation strategies beyond individual indications.

Further, our review sought to identify theoretical foundations considered during implementation and contextual factors that are associated with the effectiveness of the interventions (RQ2). However, reporting on these aspects was sparse in the identified studies and grey literature. Only a few studies reported using frameworks to guide their interventions. This limits our understanding of the mechanisms driving the observed effects and reduces the ability to replicate successful interventions in different settings, as the lack of theory-driven design makes it difficult to explain how contextual and behavioural factors influence effectiveness. For example, Stuart et al. based the components of their intervention on the core elements of the PRECEDE framework thereby aligning components with identified determinants of behaviour ([83], significant positive effect with RD ≥ 20%). Similarly, Wintemute et al. drew on Rogers’ theory of diffusion of innovations to supplement evidence-based recommendations with active reminders and local feedback ([69], significant positive effect). These examples illustrate how theoretical models can strengthen intervention design by making explicit the mechanisms through which behaviour change is expected to occur. However, despite the availability of several de-implementation frameworks, they appear to be rarely applied in practice [99]. A more consistent use of such frameworks across interventions would not only facilitate comparability but also strengthen the theoretical grounding of de-implementation strategies. Integrating examples such as digital readiness or organisational culture within such frameworks could help clarify how contextual factors interact with intervention mechanisms. Policymakers should support evidence-based interventions built on robust theoretical foundations and evaluation frameworks to ensure effectiveness and lasting impact by avoiding inefficient components. Once solid evidence has been generated through primary studies, a realist review may help to fully understand how and why different components of the intervention(s) work in what contexts and for whom [100]. The success of such system-level changes depends heavily on contextual conditions, such as the availability and interoperability of local infrastructure, prevailing funding mechanisms, and regulatory frameworks. These factors determine whether an intervention can be feasibly implemented and sustained, and they should be carefully considered when transferring findings to other settings. Although some studies reported larger effects for changes in the clinical ordering system compared to soft interventions, it cannot be assumed that such approaches are simultaneously less expensive, despite the absence of recurring training sessions and evaluations [98]. While CDSS may prove cost-efficient over time through automation and scalability, they often demand substantial initial investments in digital infrastructure. In contrast, soft interventions are typically less costly to implement initially but may require ongoing efforts to sustain their effects. This trade-off should be carefully considered when designing de-implementation strategies. Cost-effectiveness analysis is necessary to evaluate the costs of interventions relative to their savings, as interventions aimed at reducing low-value care can themselves be resource-intensive.

### Limitations

There are several limitations to this review that should be acknowledged. First, we included observational studies due to the complexity of the interventions. Observational studies are generally more prone to internal validity concerns compared to RCTs [38]. Second, we did not pool the effects of the interventions quantitatively because of the heterogeneity of the interventions and outcome measures. Instead, we focused on the evaluation of clustered results in order to give a concise overview. While the classification into structural or soft interventions is partly based on literature, the distinction is not always clear-cut, as some interventions span both categories or include borderline elements, and several interventions in this review explicitly combined soft and structural components. Alternative ways of grouping interventions and outcomes may therefore lead to different interpretations of the findings. In addition, conducting an extended mixed-methods synthesis integrating qualitative and quantitative evidence was beyond the scope of this review, given the sparse and inconsistent reporting of theoretical foundations and contextual factors. Third, we frequently observed small effects, which may limit the strength of the conclusions, though the overall trends were generally positive. Still, only 12 of the included interventions reported confidence intervals, limiting the ability to assess the reliability of effect estimates. The lack of confidence intervals makes it more difficult to determine the statistical robustness of reported changes, and increases the uncertainty surrounding the true effectiveness of the interventions. Furthermore, the majority of studies reported outcomes over relatively short timeframes, with few studies providing extensive follow-up. Consequently, it remains unclear whether reductions in TFT ordering were sustained once interventions ended or whether rebound effects occurred, for example due to alert fatigue, CDSS-related workflow integration issues, or system changes. Future research should address the long-term sustainability of de-implementation efforts. Fourth, most of the included studies were conducted in North America and Europe, particularly the USA, Canada, and the UK. This may limit the generalisability to health systems in other regions, especially those with low resources or limited digital infrastructure. In such settings, soft interventions may be more readily feasible than CDSS-based approaches that require specific digital and regulatory prerequisites. In addition, the interventions under study may not be readily transferable to other settings in the countries of interest. For example, in the German healthcare system, there are various electronic health data management systems and providers of practice management software, each with varying levels of interoperability. The context in which interventions are deployed could produce different outcomes based on the technical and regulatory landscape. Regulatory efforts are necessary in order to incorporate customised solutions across multiple institutions simultaneously. Fifth, the literature search was restricted to English and German publications. While no German-language studies were identified and several included studies originated from non-English-speaking countries, the exclusion of other languages may have resulted in the omission of a small number of relevant studies.

Regarding publication bias, research on TFT reduction is predominantly publicly funded, with fewer incentives for researchers to withhold information in comparison to pharmaceutical trials and related reviews. Any intervention aiming to reduce low-value TFT tests is likely to lead to positive results compared to usual care. Meanwhile, significant outcomes (31 interventions, 57%) were not reported in excessive frequency in relation to non-significant outcomes or outcomes with no reported level of significance. Thus, we concluded that publication bias is not a major concern. However, the predominance of non-RCTs led to a generally low CoE, limiting generalisability. Most controlled studies had a serious or critical RoB due to potential confounding, while observational studies were categorically classified as having a critical RoB. Last, we cannot rule out the possibility that our findings are influenced by selective reporting of outcomes. However, similar to the issue of potential publication bias, selective reporting is unlikely to pose a significant problem, as the body of evidence includes enough compelling and consistently positive results. The identified limitations coincide with those found in similar reviews and research on related low-value care topics [21, 96, 101]. Thus, the implications for policy should be interpreted with the appropriate caution, taking into account the GRADE results (Additional file 7).

## Conclusion

The evidence on de-implementation strategies for TFT ordering suggests that behaviour change interventions have the potential to significantly reduce excessive thyroid function testing. Particularly CDSS appear to be associated with promising results, though most studies are of high or critical risk of bias. If these findings hold in more rigorous trials, this would strengthen the evidence base for feasible workflow modifications to improve care. Policy and practice could then consider implementing controlled TFT reduction as a means to enhance appropriateness and possibly reduce costs. Continued research on cost-effectiveness will be essential to inform large-scale implementation.

Future research should focus on developing well-designed interventions based on a solid theoretical foundation and higher methodological rigour. In particular, RCTs and the use of hybrid effectiveness-implementation designs would allow more reliable evaluation and applicability. By systematically reporting contextual factors and mechanisms of change, future studies can strengthen the evidence base and support the replication of potentially effective de-implementation strategies across settings. In the short term, standardised cluster RCTs across diverse contexts could test the effectiveness of CDSS, while medium-term studies should assess the sustainability of their effects. In the longer term, theory-based realist syntheses may help clarify what works, for whom, and why. This review can help inform the design of such interventions by identifying which specific interventions or components may be associated with greater effectiveness. Ongoing evaluation of these studies can identify the mechanisms of change and facilitate the replication of successful de-implementation interventions across various settings.

## Supplementary Information

Additional file 1. Additional file 1 includes the AMSTAR assessment of the review by Zhelev et al [34].

Additional file 2. Additional file 2 includes the ROBIS assessment of the review by Zhelev et al. [34].

Additional file 3. Additional file 3 includes the PRISMA 2020 Checklist.

Additional file 4. Additional file 4 includes changes made to the information provided at registration.

Additional file 5. Additional file 5 includes the search strategies in Embase, Medline, Scopus, Cochrane, and Google Scholar.

Additional file 6. Additional file 6 includes the list of data items extracted in the review.

Additional file 7. Additional file 7 includes the full GRADE assessment of the interventions.

Additional file 8. Additional file 8 includes the information on all articles excluded after full-text screening, including reason for exclusion.

Additional file 9. Additional file 9 includes additional information on study characteristics, i.e. reported outcomes, reporting on theoretical foundations, and study period.

Additional file 10. Additional file 10 includes the visualisation of the Risk of Bias assessment for the (cluster) RCTs and controlled studies.

Additional file 11. Additional file 11 includes additional information on study results, i.e. the outcome values (pre/postintervention), notes on outcome measures and statistical indicators (confidence interval, p-value, relative reduction, difference in means).

Additional file 12. Additional file 12 includes additional information on study characteristics, i.e. funding and reported conflict of interest.

## Data Availability

All data generated or analysed during this study are included in this published article and its supplementary information files.

## Abbreviations

CDSS: Clinical decision support systems
CoE: Certainty of evidence
CoV: Coefficient of variation
CW: Choosing Wisely
GRADE: Grading of Recommendations, Assessment, Development, and Evaluations
NRSI: Non-randomised studies of interventions
NS: Not significant
PDSA: Plan-Do-Study-Act
PRISMA: Preferred Reporting Items for Systematic Reviews and Meta-Analyses
RCT: Randomised controlled trial
RQ: Research question
RoB: Risk of bias
RoB 2: RoB 2.0 tool
ROBINS-I: RoB In Non-randomised Studies—of Interventions
ROBIS: RoB In Systematic reviews tool
T3: Triiodothyronine
T4: Thyroxine
TDF: Theoretical Domains Framework
TFT: Thyroid function test
TSH: Thyroid-stimulating hormone
