# Supplementary material for: Effectiveness and mechanisms of interventions to reduce low-value thyroid function tests: a systematic review
Source: Syst Rev. 2026 Feb 25;15:111. doi: 10.1186/s13643-026-03119-8 (PMC13040701; doi:10.1186/s13643-026-03119-8)
Supplement: Supplementary file 1 — Additional file 1. Additional file 1 includes the AMSTAR assessment of the review by Zhelev et al [34]. [file 13643_2026_3119_MOESM1_ESM.docx]

# **AMSTAR Assessment**

| **Domains*   Paper** | **1** | **2** | **3** | **4** | **5** | **6** | **7** | **8** | **9 (RCTs)** | **9**  **(NRSI)** | **10** | **11**  **(RCTs)** | **11**  **(NRSI)** | **12** | **13** | **14** | **15** | **16** | **AMSTAR 5-item**  **(-2 & -7)** | **AMSTAR 7-item** | **Pieper et al. 5-item** |
| --- | --- | --- | --- | --- | --- | --- | --- | --- | --- | --- | --- | --- | --- | --- | --- | --- | --- | --- | --- | --- | --- |
| Zhelev et al. 2016 (1) | Y | Y | N | PY | Y | Y | N | Y | PY | PY | N | NA | NA | NA | Y | Y | NA | Y | Moderate | Low | Moderate |

**Abbreviations:** N = No, NA = Not Applicable, NRSI = Non-randomised Studies of Interventions, PY = Probably Yes, RCTs = Randomised-controlled trials, Y = Yes.

Grey-coloured cells: Selected critical domains of the modified AMSTAR 5-item assessment. Orange: Assessment chosen for publication.

**Domains:**

**1.** Did the research questions and inclusion criteria for the review include the components of PICO?
**2.** Did the report of the review contain an explicit statement that the review methods were established prior to the conduct of the review and did the report justify any significant deviations from the protocol
**3.** Did the review authors explain their selection of the study designs for inclusion in the review?
**4.** Did the review authors use a comprehensive literature search strategy?
**5.** Did the review authors perform study selection in duplicate?
**6.** Did the review authors perform data extraction in duplicate?
**7.** Did the review authors provide a list of excluded studies and justify the exclusions?
**8.** Did the review authors describe the included studies in adequate detail?
**9.** Did the review authors use a satisfactory technique for assessing the risk of bias (RoB) in individual studies that were included in the review?
**10.** Did the review authors report on the sources of funding for the studies included in the review?
**11.** If meta-analysis was performed did the review authors use appropriate methods for statistical combination of results?
**12.** If meta-analysis was performed, did the review authors assess the potential impact of RoB in individual studies on the results of the meta-analysis or other evidence synthesis?
**13.** Did the review authors account for RoB in individual studies when interpreting/discussing the results of the review?
**14.** Did the review authors provide a satisfactory explanation for, and discussion of, any heterogeneity observed in the results of the review?
**15.** If they performed quantitative synthesis did the review authors carry out an adequate investigation of publication bias (small study bias) and discuss its likely impact on the results of the review?
**16.** Did the review authors report any potential sources of conflict of interest, including any funding they received for conducting the review?

The methodological quality of the systematic review by Zhelev et al. (1) was independently evaluated by two authors (CP and MH) using the AMSTAR (A MeaSurement Tool to Assess systematic Reviews) 2 tool. The review was assessed across 16 domains and rated as ‘high’, ‘moderate’, ‘low’, or ‘very low’ based on critical domains (2). Following the approach by Wittich et al., two (items 2 and 7) of the seven critical domains (items 2, 4, 7, 9, 11, 13, 15) reported by Shea et al. were excluded, as they were considered non-essential for this update. Consequently, a modified system with five critical domains (items 4, 9, 11, 13, 15) was used for the evaluation (3). Additionally, an evaluation was conducted using the AMSTAR rating scheme by Pieper et al. to ensure that excluding items 2 and 7 did not significantly distort the results (4). Using the modified system with five critical domains, the review by Zhelev et al. (1) was found to be of moderate quality. The evaluation with Pieper et al.'s (2021) AMSTAR rating scheme also led to a moderate quality assessment.

**Literature Cited**

1. Zhelev Z, Abbott R, Rogers M, Fleming S, Patterson A, Hamilton WT et al. Effectiveness of interventions to reduce ordering of thyroid function tests: a systematic review. BMJ open 2016; 6(6):e010065.

2. Shea BJ, Reeves BC, Wells G, Thuku M, Hamel C, Moran J et al. AMSTAR 2: a critical appraisal tool for systematic reviews that include randomised or non-randomised studies of healthcare interventions, or both. BMJ 2017; 358:j4008.

3. Wittich L, Tsatsaronis C, Kuklinski D, Schöner L, Steinbeck V, Busse R et al. Patient-Reported Outcome Measures as an Intervention: A Comprehensive Overview of Systematic Reviews on the Effects of Feedback. Value Health 2024; 27(10):1436-1453.

4. Pieper D, Lorenz RC, Rombey T, Jacobs A, Rissling O, Freitag S et al. Authors should clearly report how they derived the overall rating when applying AMSTAR 2-a cross-sectional study. Journal of clinical epidemiology 2021; 129:97–103.
