# Supplementary material for: Effectiveness and mechanisms of interventions to reduce low-value thyroid function tests: a systematic review
Source: Syst Rev. 2026 Feb 25;15:111. doi: 10.1186/s13643-026-03119-8 (PMC13040701; doi:10.1186/s13643-026-03119-8)
Supplement: Supplementary file 2 — Additional file 2. Additional file 2 includes the ROBIS assessment of the review by Zhelev et al. [34]. [file 13643_2026_3119_MOESM2_ESM.docx]

# ROBIS Assessment

ROBIS (Tool to Assess Risk of Bias in Systematic Reviews) Assessment of the Systematic Review by Zhelev et al. (2016, (1)).

## Phase 1: Assessing Relevance

## Intervention Reviews:

| **Category** | **Target Question (e.g. overview or guideline)** | **Review Being Assessed** |
| --- | --- | --- |
| **Patients/Population(s):** | Adults and children having thyroid function tests | Zhelev et al. 2016 (1) |
| **Intervention(s):** | Structured education programme; Audit and feedback; Dissemination of evidence-based guidelines or memorandum; Incentive approaches; Administrative interventions |  |
| **Comparator(s):** | Usual care |  |
| **Outcome(s):** | Test rate, Appropriateness, Expenditure, Pattern, Coefficient of Variation, User Satisfaction |  |

**Does the question addressed by the review match the target question?** YES/NO/UNCLEAR

## Phase 2: Identifying Concerns with the Review Process

### DOMAIN 1: STUDY ELIGIBILITY CRITERIA

| **Signaling Question** | **Rating (Y/PY/PN/N/NI)** | **Reasoning** |
| --- | --- | --- |
| 1.1 Did the review adhere to pre-defined objectives and eligibility criteria? | Y | See protocol 🡪 predefined search strategy. |
| 1.2 Were the eligibility criteria appropriate for the review question? | Y | Criteria designed to identify relevant studies. |
| 1.3 Were eligibility criteria unambiguous? | PY |  |
| 1.4 Were any restrictions in eligibility criteria based on study characteristics appropriate (e.g. date, sample size, study quality, outcomes measured)? | PY | No restrictions besides inclusion criteria + scientific literature. |
| 1.5 Were any restrictions in eligibility criteria based on sources of information appropriate (e.g. publication status or format, language, availability of data)? | PY | See above. |

**Concerns regarding specification of study eligibility criteria:** LOW/HIGH/UNCLEAR

**Rationale for concern:**

### DOMAIN 2: IDENTIFICATION AND SELECTION OF STUDIES

| **Signaling Question** | **Rating (Y/PY/PN/N/NI)** | **Reasoning** |
| --- | --- | --- |
| 2.1 Did the search include an appropriate range of databases/electronic sources for published and unpublished reports? | Y | From protocol: The search included MEDLINE, EMBASE, and Cochrane Database, along with other methods such as hand searching and contacting experts. |
| 2.2 Were methods additional to database searching used to identify relevant reports? | PY | See above. |
| 2.3 Were the terms and structure of the search strategy likely to retrieve as many eligible studies as possible? | PY | Comprehensive search strategy. |
| 2.4 Were restrictions based on date, publication format, or language appropriate? | Y | See domain 1.4. |
| 2.5 Were efforts made to minimise error in selection of studies? | Y | Two reviewers independently screened and assessed studies, with a third reviewer resolving discrepancies. |

**Concerns regarding methods used to identify and/or select studies:** LOW/HIGH/UNCLEAR

**Rationale for concern:**

### DOMAIN 3: DATA COLLECTION AND STUDY APPRAISAL

| **Signaling Question** | **Rating (Y/PY/PN/N/NI)** | **Reasoning** |
| --- | --- | --- |
| 3.1 Were efforts made to minimise error in data collection? | PY | From protocol: Data will be extracted from included studies by one reviewer into a bespoke database and checked by another reviewer. Discrepancies will be resolved by discussion, with the involvement of a third reviewer if necessary. |
| 3.2 Were sufficient study characteristics available for both review authors and readers to be able to interpret the results? | Y |  |
| 3.3 Were all relevant study results collected for use in the synthesis? | PN | Calculation of large effect (effect size) not comprehensible/ raw data not available. |
| 3.4 Was risk of bias (or methodological quality) formally assessed using appropriate criteria? | PN | EPHPP Tool; allocation concealment was not assessed. |
| 3.5 Were efforts made to minimise error in risk of bias assessment? | Y | Independently by ZZ and RA. |

**Concerns regarding methods used to collect data and appraise studies:** LOW/HIGH/UNCLEAR

**Rationale for concern:**

### DOMAIN 4: SYNTHESIS AND FINDINGS

| **Signaling Question** | **Rating (Y/PY/PN/N/NI)** | **Reasoning** |
| --- | --- | --- |
| 4.1 Did the synthesis include all studies that it should? | Y |  |
| 4.2 Were all pre-defined analyses reported or departures explained? | PY | No significant changes in protocol. |
| 4.3 Was the synthesis appropriate given the nature and similarity in the research questions, study designs and outcomes across included studies? | PY | Due to nature of studies no meta-analysis. Thorough descriptive reporting. |
| 4.4 Was between-study variation (heterogeneity) minimal or addressed in the synthesis? | PY | We deemed pooling the results inappropriate and were unable to use statistical methods to investigate the impact of various study and intervention characteristics on the reported outcomes. |
| 4.5 Were the findings robust, e.g. as demonstrated through funnel plot or sensitivity analyses? | PY | Visual inspection of the data suggests, however, that differences such as intervention type, study design, setting and year of publication have little or no impact on the reported effectiveness. |
| 4.6 Were biases in primary studies minimal or addressed in the synthesis? | Y | Biases were mostly high but addressed due to nature of review. |

**Concerns regarding the synthesis and findings:** LOW/HIGH/UNCLEAR

**Rationale for concern: Biases and difficult aggregated reporting due to nature of heterogenic results of intervention studies were addressed.**

**Phase 3: Judging Risk of Bias**

**Summarize the concerns identified during the Phase 2 assessment:**

| **Domain** | **Concern** | **Rationale for Concern** |
| --- | --- | --- |
| 1. Concerns regarding specification of study eligibility criteria | LOW | - |
| 2. Concerns regarding methods used to identify and/or select studies | LOW | Excluded studies were not listed; no indication for influencing results. |
| 3. Concerns regarding methods used to collect data and appraise studies | HIGH | EPHPP Tool + Effect size raw data not available. |
| 4. Concerns regarding the synthesis and findings | LOW | - |

## RISK OF BIAS IN THE REVIEW

| **Question** | **Rating (Y/PY/PN/N/NI)** | **Reasoning** |
| --- | --- | --- |
| A. Did the interpretation of findings address all of the concerns identified in Domains 1 to 4? | PY | The main limitation is that the quality of evidence did not allow strong conclusions and more specific recommendations to be made. Furthermore, the disparate methods, populations of study, interventions and outcome measures made pooled synthesis of results impossible. Thus, we have chosen to present the results as a narrative synthesis. Similarly, although we strongly suspect that publication bias and selective reporting of outcomes may be operating, particularly for the non-randomised study designs, we could neither investigate nor attempt to quantify the potential impact. |
| B. Was the relevance of identified studies to the review's research question appropriately considered? | PY | The identified evidence is directly relevant to this particular test ordering behaviour and could be used to guide the design and implementation of future intervention programmes as well as the development of research projects that could address the identified gaps in knowledge. |
| C. Did the reviewers avoid emphasizing results on the basis of their statistical significance? | Y | Neutral reporting |

**Risk of bias in the review:** RISK: LOW/HIGH/UNCLEAR

**Rationale for risk: Though there are some concerns, none indicates the study to be of high risk / the concerns were addressed in the conclusion section respectively**

**Abbreviations**: N = No, NI = No Information, PN = Probably No, PY = Probably Yes, Y = Yes.

Literature Cited

1. Zhelev Z, Abbott R, Rogers M, Fleming S, Patterson A, Hamilton WT et al. Effectiveness of interventions to reduce ordering of thyroid function tests: a systematic review. BMJ open 2016; 6(6):e010065.
