# Supplementary material for: Effectiveness and mechanisms of interventions to reduce low-value thyroid function tests: a systematic review
Source: Syst Rev. 2026 Feb 25;15:111. doi: 10.1186/s13643-026-03119-8 (PMC13040701; doi:10.1186/s13643-026-03119-8)
Supplement: Supplementary file 4 — Additional file 4. Additional file 4 includes changes made to the information provided at registration. [file 13643_2026_3119_MOESM4_ESM.docx]

# Changes made to the information provided at registration

| **Domain** | **Change** | **Explanation** |
| --- | --- | --- |
| **Title** | “inappropriate” was changed to “low-value”  “an update of” was removed  “Effectiveness” was changed to “Effectiveness and mechanisms” | Low-value corresponds to the wording in current research.  Framing as new systematic review and inclusion of mechanisms due to reviewer suggestions to clarify the method and aim |
| **Research team** | The research team was extended to involve a researcher with expert knowledge in evidence synthesis of reviews (GG). | We made additions to the team to increase the team’s methodological expertise. |
| **Dates** | The completion date has been adjusted from 31 March 2024 to 30 September 2024. | The comprehensive data extraction, synthesis, and assessment of all studies, including both newly identified and previously included studies, required more time than initially anticipated. |
| **Data extraction** | We did not restrict data extraction to a specific number of studies per reviewer. Instead, both reviewers (CP, MH) independently extracted data from all included studies. Consequently, we did not calculate agreement between reviewers following calibration as originally planned. | We wanted to ensure a thorough and consistent data extraction process across all studies. |
| **Data items** | Instead of classifying the setting as primary care or hospital, we used the following categories: inpatient care, outpatient care, emergency department. | This change was made to provide a more detailed and precise categorisation of the settings. |
| **Risk of bias assessment** | We did not restrict risk of bias assessment to a specific number of studies per reviewer. Instead, both reviewers (CP, MH) independently assessed the risk of bias for all included studies. Consequently, we did not calculate agreement between reviewers as originally planned. | We wanted to ensure a more comprehensive and consistent assessment of risk of bias across all studies. |
| **Data synthesis** | We categorised the interventions according to the existing typology of behaviour change intervention types described in the protocol. Additionally, we differentiated between structural interventions (changes in funding, decision tools) and nudging interventions (education, guidelines, feedback, reminders). Outcomes were divided into two categories: volume reduction (test rates, expenditure) and improvement of care (appropriateness, pattern, coefficient of variation). | This refined categorisation enhances the clarity and precision of our analysis, allowing for a more nuanced understanding of the different types of interventions and their respective impacts on healthcare outcomes. |
| **Reporting of amendments** | We did not report important protocol amendments in an updated version of the PROSPERO record. | We did not change the anticipated completion date of our review in PROSPERO in time and were consequently unable to amend the PROSPERO record after that date. Thus, we report all protocol amendments here. |
