# Supplementary material for: Effectiveness and mechanisms of interventions to reduce low-value thyroid function tests: a systematic review
Source: Syst Rev. 2026 Feb 25;15:111. doi: 10.1186/s13643-026-03119-8 (PMC13040701; doi:10.1186/s13643-026-03119-8)
Supplement: Supplementary file 5 — Additional file 5. Additional file 5 includes the search strategies in Embase, Medline, Scopus, Cochrane, and Google Scholar. [file 13643_2026_3119_MOESM5_ESM.docx]

# **Search strategy**

## Embase

| **Number of Records** | **Date #1** | **Update #2** |
| --- | --- | --- |
| #1: 525  #2: 577 | 21st November 2023 | 9th July 2024 |

| **#** | **Query** | **Hits #1** | **Hits #2** |
| --- | --- | --- | --- |
| 1 | Thyroid Function Tests/ | 14,543 | 15,373 |
| 2 | (thyroid function adj (test or tests or testing)).ti,ab. | 8,068 | 8,469 |
| 3 | (thyroid adj2 screening).ti,ab. | 1,787 | 1,869 |
| 4 | (test* adj5 thyroid).ti,ab. | 15,977 | 16,696 |
| 5 | (TSH adj5 test*).ti,ab. | 3,942 | 4,110 |
| 6 | (T3 adj5 test*).ti,ab. | 2,191 | 2,276 |
| 7 | (FT3 adj5 test*).ti,ab. | 355 | 366 |
| 8 | (TT3 adj5 test*).ti,ab. | 57 | 60 |
| 9 | (T4 adj5 test*).ti,ab. | 1,840 | 1,903 |
| 10 | (FT4 adj5 test*).ti,ab. | 779 | 819 |
| 11 | (TT4 adj5 test*).ti,ab. | 92 | 96 |
| 12 | thyroid stimulating hormone test*.ti,ab. | 62 | 64 |
| 13 | (triiodothyronine adj5 test*).ti,ab. | 492 | 505 |
| 14 | (thyrotropin adj5 test*).ti,ab. | 886 | 902 |
| 15 | TSH assay*.ti,ab. | 569 | 581 |
| 16 | thyrotropin assay*.ti,ab. | 106 | 106 |
| 17 | or/1-16 | 31,260 | 32,695 |
| 18 | (test* adj5 request*).ti,ab. | 7,323 | 7,763 |
| 19 | (test* adj2 practic*).ti,ab. | 7,840 | 8,182 |
| 20 | (test* adj5 order*).ti,ab. | 44,100 | 45,337 |
| 21 | (unnecessary adj5 test*).ti,ab. | 5,458 | 6,800 |
| 22 | (inappropriate* adj5 test*).ti,ab. | 2,641 | 2,771 |
| 23 | (test* adj5 strateg*).ti,ab. | 29,571 | 31,003 |
| 24 | (test* adj5 guidelines).ti,ab. | 14,020 | 14,714 |
| 25 | (appropriate* adj5 test*).ti,ab. | 37,550 | 39,162 |
| 26 | (misuse adj4 test*).ti,ab. | 360 | 369 |
| 27 | or/18-26 | 140,767 | 146,499 |
| 28 | 17 and 27 | 896 | 948 |
|  | Filter in Endnote: Published since 01/01/2014 | 525 | 577 |

## Medline

| **Number of Records** | **Date #1** | **Update #2** |
| --- | --- | --- |
| #1: 1,524  #2: 1,572 | 21st November 2023 | 9th July 2024 |

| **#** | **Query** | **Hits #1** | **Hits #2** |
| --- | --- | --- | --- |
| 1 | Thyroid Function Tests/ | 15,393 | 15,478 |
| 2 | Laboratories, Hospital/ | 4,868 | 4,881 |
| 3 | Clinical Laboratory Techniques/ | 23,932 | 24,016 |
| 4 | Clinical Chemistry Tests/ | 1,586 | 1,588 |
| 5 | Diagnostic Tests, Routine/ | 15,205 | 15,334 |
| 6 | (thyroid function adj (test or tests or testing)).ti,ab. | 4,456 | 4,583 |
| 7 | (test* adj5 thyroid).ti,ab. | 10,100 | 10,342 |
| 8 | (TSH adj5 test*).ti,ab. | 1,943 | 1,977 |
| 9 | (T3 adj5 test*).ti,ab. | 1,495 | 1,550 |
| 10 | (FT3 adj5 test*).ti,ab. | 159 | 162 |
| 11 | (TT3 adj5 test*).ti,ab. | 32 | 32 |
| 12 | (T4 adj5 test*).ti,ab. | 1,104 | 1,128 |
| 13 | (FT4 adj5 test*).ti,ab. | 320 | 329 |
| 14 | (TT4 adj5 test*).ti,ab. | 58 | 59 |
| 15 | thyroid stimulating hormone test*.ti,ab. | 48 | 50 |
| 16 | (triiodothyronine adj5 test*).ti,ab. | 537 | 544 |
| 17 | (thyrotropin adj5 test*).ti,ab. | 790 | 802 |
| 18 | TSH assay*.ti,ab. | 382 | 386 |
| 19 | thyrotropin assay*.ti,ab. | 84 | 84 |
| 20 | or/1-19 | 70,501 | 71,118 |
| 21 | (test* adj5 request*).ti,ab. | 3,718 | 3,835 |
| 22 | (test* adj2 practic*).ti,ab. | 5,832 | 6,080 |
| 23 | (test* adj5 order*).ti,ab. | 30,077 | 30,635 |
| 24 | (unnecessary adj5 test*).ti,ab. | 3,638 | 3,820 |
| 25 | (inappropriate* adj5 test*).ti,ab. | 1,549 | 1,610 |
| 26 | (test* adj5 strateg*).ti,ab. | 22,017 | 23,025 |
| 27 | (test* adj5 guidelines).ti,ab. | 8,061 | 8,410 |
| 28 | (appropriate* adj5 test*).ti,ab. | 20,540 | 21,288 |
| 29 | (misuse adj4 test*).ti,ab. | 280 | 287 |
| 30 | or/21-29 | 90,932 | 94,019 |
| 31 | Unnecessary Procedures/ | 6,202 | 6,226 |
| 32 | 30 and 31 | 532 | 539 |
| 33 | 30 and 20 | 3,103 | 3,146 |
| 34 | 32 or 33 | 3,469 | 3,517 |
|  | Filter in Endnote: Published since 01/01/2014 | 1,524 | 1,572 |

## Scopus

| **Number of Records** | **Date #1** | **Update #2** |
| --- | --- | --- |
| #1: 714  #2: 738 | 21st November 2023 | 9th July 2024 |

| **#** | **Query** | **Hits #1** | **Hits #2** |
| --- | --- | --- | --- |
| 1 | TITLE-ABS ( "thyroid function" W/1 ( test OR tests OR testing ) ) | 5,779 | 5,911 |
| 2 | TITLE-ABS ( thyroid W/2 screening ) | 1,811 | 1,866 |
| 3 | TITLE-ABS ( test* W/5 thyroid ) | 13,258 | 13,588 |
| 4 | TITLE-ABS ( tsh W/5 test* ) | 2,872 | 2,936 |
| 5 | TITLE-ABS ( t3 W/5 test* ) | 2,762 | 2,872 |
| 6 | TITLE-ABS ( ft3 W/5 test* ) | 283 | 288 |
| 7 | TITLE-ABS ( tt3 W/5 test* ) | 57 | 59 |
| 8 | TITLE-ABS ( t4 W/5 test* ) | 2,080 | 2,149 |
| 9 | TITLE-ABS ( ft4 W/5 test* ) | 529 | 546 |
| 10 | TITLE-ABS ( tt4 W/5 test* ) | 88 | 93 |
| 11 | TITLE-ABS ( triiodothyronine W/5 test* ) | 756 | 775 |
| 12 | TITLE-ABS ( thyrotropin W/5 test* ) | 974 | 983 |
| 13 | TITLE-ABS ( "tsh assay*" ) | 486 | 489 |
| 14 | TITLE-ABS ( "thyrotropin assay*" ) | 100 | 100 |
| 15 | #1 OR #2 OR #3 OR #4 OR #5 OR #6 OR #7 OR #8 OR #9 OR #10 OR #11 OR #12 OR #13 OR #14 | 21,056 | 21,635 |
| 16 | TITLE-ABS ( test* W/5 request* ) | 6,536 | 6,793 |
| 17 | TITLE-ABS ( test* W/2 practic* ) | 38,935 | 40,676 |
| 18 | TITLE-ABS ( test* W/5 order* ) | 115,657 | 118,371 |
| 19 | TITLE-ABS ( unnecessary W/5 test* ) | 5,119 | 5,360 |
| 20 | TITLE-ABS ( inappropriate* W/5 test* ) | 2,654 | 2,741 |
| 21 | TITLE-ABS ( test* W/5 strateg* ) | 60,495 | 63,490 |
| 22 | TITLE-ABS ( test* W/5 guidelines ) | 18,495 | 19,357 |
| 23 | TITLE-ABS ( appropriate* W/5 test* ) | 42,484 | 43,948 |
| 24 | TITLE-ABS ( misuse W/4 test* ) | 639 | 653 |
| 25 | #16 OR #17 OR #18 OR #19 OR #20 OR #21 OR #22 OR #23 OR #24 | 280,132 | 290,098 |
| 26 | #15 AND #25 | 714 | 738 |

## Cochrane

| **Number of Records** | **Date #1** | **Update #2** |
| --- | --- | --- |
| #1: 84 Trials, 7 Cochrane Reviews  #2: 89 Trials, 7 Cochrane Reviews | 21st November 2023 | 9th July 2024 |

| **#** | **Query** | **Hits #1** | **Hits #2** |
| --- | --- | --- | --- |
| 1 | MeSH descriptor: [Thyroid Function Tests] explode all trees | 280 | 310 |
| 2 | MeSH descriptor: [Clinical Chemistry Tests] explode all trees | 10160 | 10,608 |
| 3 | MeSH descriptor: [Diagnostic Tests, Routine] explode all trees | 337 | 382 |
| 4 | ("thyroid function" next (test*)):ti,ab | 324 | 356 |
| 5 | (test* near/5 thyroid):ti,ab | 653 | 698 |
| 6 | (TSH near/5 test*):ti,ab | 219 | 235 |
| 7 | (T3 near/5 test*):ti,ab | 267 | 297 |
| 8 | (FT3 near/5 test*):ti,ab | 22 | 23 |
| 9 | (TT3 near/5 test*):ti,ab | 5 | 4 |
| 10 | (T4 near/5 test*):ti,ab | 150 | 159 |
| 11 | (FT4 near/5 test*):ti,ab | 27 | 28 |
| 12 | (TT4 near/5 test*):ti,ab | 3 | 3 |
| 13 | (triiodothyronine near/5 test*):ti,ab | 25 | 25 |
| 14 | (thyrotropin near/5 test*):ti,ab | 25 | 26 |
| 15 | (tsh next assay*):ti,ab | 12 | 12 |
| 16 | (thyrotropin next assay*):ti,ab | 1 | 1 |
| 17 | #1 OR #2 OR #3 OR #4 OR #5 OR #6 OR #7 OR #8 OR #9 OR #10 OR #11 OR #12 #13 OR #14 OR #15 OR #16 | 11802 | 12,405 |
| 18 | (test* near/5 request*):ti,ab | 359 | 377 |
| 19 | (test* near/5 practic*):ti,ab | 2230 | 2,356 |
| 20 | (test* near/5 order*):ti,ab | 4096 | 4,285 |
| 21 | (unnecessary near/5 test*):ti,ab | 201 | 216 |
| 22 | (inappropriate* near/5 test*):ti,ab | 120 | 128 |
| 23 | (test* near/5 strateg*):ti,ab | 2728 | 2,884 |
| 24 | (test* near/5 guidelines):ti,ab | 657 | 722 |
| 25 | (appropriate* near/5 test*):ti,ab | 3071 | 3,256 |
| 26 | (misuse near/4 test*):ti,ab | 30 | 31 |
| 27 | #18 OR #19 OR #20 OR #21 OR #22 OR #23 OR #24 OR #25 OR #26 | 12834 | 13,564 |
| 28 | #17 AND #27 | 200 | 208 |
|  | Filter in Endnote: Published since 01/01/2014 | 91 | 96 |
|  | Trials | 84 | 89 |
|  | Cochrane Reviews | 7 | 7 |

## Google Scholar

| **Number of Records** | **Date #1** | **Update #2** |
| --- | --- | --- |
| #1 First 300  #2 First 300 (129 new hits) | 21st November 2023 | 9th July 2024 |

| **#** | **Query** | **Hits #1** | **Hits #2** |
| --- | --- | --- | --- |
| 1 | thyroid AND test AND (inappropriate OR unnecessary) | ca. 188,000 | ca. 189,000 |
