# Supplementary material for: Effectiveness and mechanisms of interventions to reduce low-value thyroid function tests: a systematic review
Source: Syst Rev. 2026 Feb 25;15:111. doi: 10.1186/s13643-026-03119-8 (PMC13040701; doi:10.1186/s13643-026-03119-8)
Supplement: Supplementary file 6 — Additional file 6. Additional file 6 includes the list of data items extracted in the review. [file 13643_2026_3119_MOESM6_ESM.docx]

**Data items**

| **Data item** | **Description** |
| --- | --- |
| **General study characteristics** | |
| Publication details | Author(s) |
|  | Publication year |
|  | Study design ((cluster) RCT, controlled, before and after, time series analysis) |
| Funding and conflict of interest | Funding category (non-profit, for-profit, mixed, no funding, not reported) |
|  | Name of funder (sponsor for registration record) |
|  | Conflict of interest |
|  | Ethics approval (approved, not required, not reported) |
| Location | Country |
| Target tests | Type(s) of TFTs |
|  | Number of non-TFTs related tests |
| Intervention | Setting (inpatient, outpatient, ED) |
|  | Timeframe and duration (months): preintervention, intervention + postintervention |
|  | Intervention type based on Solomon et al. (1) and Oxman et al. (2)   \| **Type** \| **Description** \| \| --- \| --- \| \| Educational programmes \| Educational interventions \| \| Guidelines and protocols \| Guideline and protocol development and implementation \| \| Changes to funding \| Changes to funding policy \| \| Reminders \| Reminders of existing guidelines and protocols \| \| Decision tools* \| Clinical decision support systems (CDSS), including test request forms and computer-based decision support \| \| Audit and Feedback \| Audit and feedback \|   * Divided into the following sub-types: reflex/automatic discharge, change of order form, alert, and cost display  Further, we grouped the interventions into structural (changes in funding, CDSS) and soft (education, guidelines, reminders, feedback) interventions. |
|  | Theoretical foundations and contextual factors that have been considered in the development of the intervention. |
| **Results** | |
| Preintervention characteristics | Level preintervention depending on outcome measure, per intervention/control group |
| Postintervention characteristics | Level postintervention depending on outcome measure, per intervention/control group |
| Outcome measure | Change in the total number of thyroid function tests, number of inappropriately ordered TFTs, test-related expenditure, health benefits to individual patients   \| **Outcome** \| **Description** \| \| --- \| --- \| \| Test numbers or rates \| Volume of ordered TFTs \| \| Expenditure \| Costs associated with TFTs \| \| Pattern \| Combination of different tests requested together \| \| Appropriateness \| e.g., adherence to guidelines, non-repeated testing (different definitions used) \| \| Coefficient of Variation \| Variability in test ordering rates among physicians relative to the average rate \|   Further, we grouped the outcome measures into two outcome categories: volume reduction (test rates, expenditure) and improvement of care (pattern, appropriateness, coefficient of variation). |
| Effect | Direction (positive/negative) |
|  | Relative change (improvement/deterioration) |
|  | Difference in means |
|  | Confidence interval |
|  | Significance (p-value) |

If data were not reported in the articles resulting in incomplete data-extraction, this was recorded. We did not contact the corresponding authors in case of missing or unclear data.

**Abbreviations:** CDSS = Clinical Decision Support System; ED = Emergency Department; RCT = Randomised Controlled Trial; TFT = Thyroid Function Test.

**References**

1. Solomon DH, Hashimoto H, Daltroy L, et al. Techniques to improve physicians’ use of diagnostic tests: a new conceptual framework. JAMA 1998;280:2020–7.

2. Oxman AD, Thomson MA, Davis DA, et al. No magic bullets: a systematic review of 102 trials of interventions to improve professional practice. CMAJ 1995;153:1423–31.
