# Supplementary material for: Effectiveness and mechanisms of interventions to reduce low-value thyroid function tests: a systematic review
Source: Syst Rev. 2026 Feb 25;15:111. doi: 10.1186/s13643-026-03119-8 (PMC13040701; doi:10.1186/s13643-026-03119-8)
Supplement: Supplementary file 7 — Additional file 7. Additional file 7 includes the full GRADE assessment of the interventions. [file 13643_2026_3119_MOESM7_ESM.docx]

# **Grading of Interventions**

The interventions in our review were evaluated using the Grading of Recommendations, Assessment, Development, and Evaluations (GRADE) approach, following the guide for complex interventions (1, 2). We graded the evidence both by grouped intervention and outcomes (Table 1; grouping in Figure 1) and by individual outcome (Table 2).


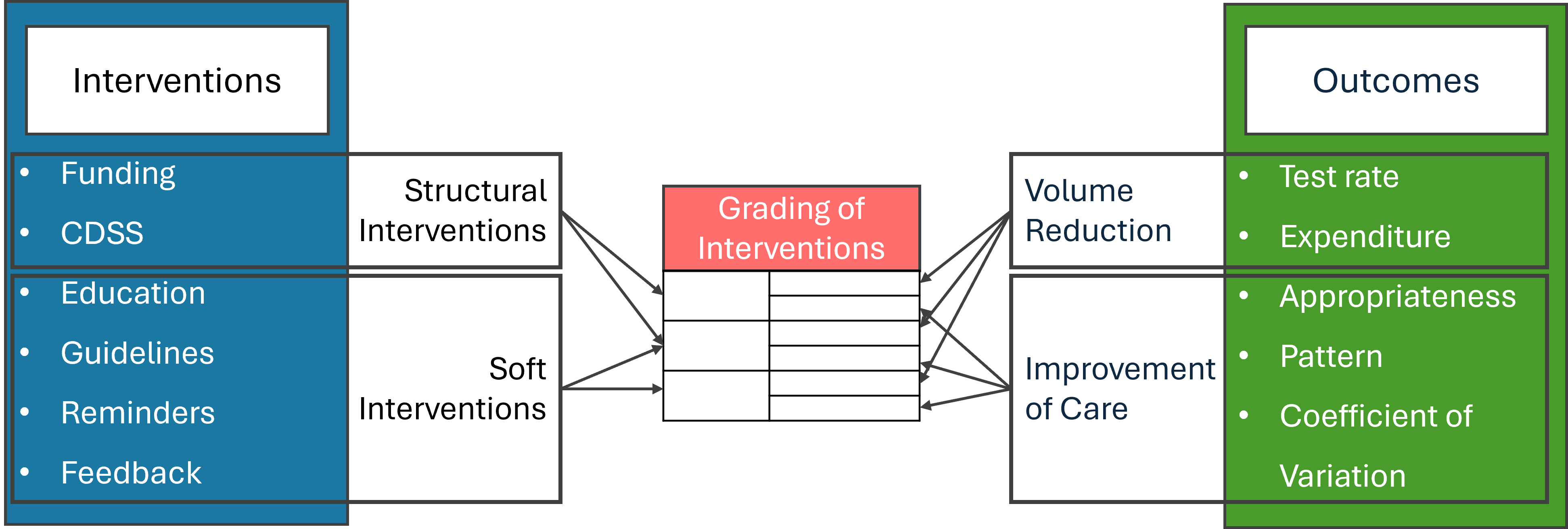


Figure 1: Visualisation of intervention and outcome grouping. CDSS = Clinical Decision Support System.

Table 1: Summary of GRADE results. Interventions grouped by structural interventions (change in funding, decision tools) and soft interventions (education, guidelines, feedback, reminder). Combined interventions reported separately. Outcomes grouped by volume reduction (test rates, expenditure) and improvement of care (appropriateness, pattern, coefficient of variation). All outcomes were considered to be of critical importance. For visualisation of grouping see Figure 1.

| **Certainty assessment** | | | | | | | **Effect ^c^** | **Certainty** | **References** |
| --- | --- | --- | --- | --- | --- | --- | --- | --- | --- |
| **No. of interventions (studies) ^a^** | **Study design** | **Risk of bias** | **Inconsistency** | **Indirectness** | **Imprecision** | **Other considerations** |  |  |  |
| **Structural interventions: 19 (19)** | | | | | | | | |  |
| **Volume reduction** | | | | | | | | |  |
| 1 (1) | RCT | Serious ^b^ | Not serious | Not serious | Serious ^d^ | None | Positive direction. NS. RR 10%. | ⨁⨁◯◯  Low | (3) |
| 16 (16) | 3 controlled studies,  13 uncontrolled studies | Serious ^b^ | Not serious | Not serious | Not serious | None | Positive direction in all 16 interventions. Significant difference in nine, six NR. RR >= 20% in 12 interventions (eight significant). RR range 2% - 81% | ⨁◯◯◯  Very Low | (4–19) |
| **Improvement of care** | | | | | | | | |  |
| 2 (2) | 2 cluster RCTs | Not Serious | Not serious | Not serious | Serious ^d^ | None | Positive direction and significant difference in both interventions. RR range 11% – 30% | ⨁⨁⨁⨁  Moderate | (20, 21) |
| 4 (4) | 4 uncontrolled studies | Not Serious | Not serious | Not serious | Not serious | None | Positive direction in all four interventions. Significant difference in two, two NR. RR >= 20% in three interventions (two significant). RR range 8% - 93%. | ⨁◯◯◯  Very Low | (15, 16, 11, 9) |
| **Combined interventions: 10 (10)** | | | | | | | | |  |
| **Volume reduction** | | | | | | | | |  |
| 8 (8) | 1 controlled study,  7 uncontrolled studies | Serious ^b^ | Not serious | Not serious | Not serious | None | Positive direction in all eight interventions. Significant difference in three, four NR. RR >= 20% in five interventions (two significant). RR range 12% - 96%. | ⨁◯◯◯  Very Low | (22–29) |
| **Improvement of care** | | | | | | | | |  |
| 1 (1) | 1 cluster RCT | Not Serious | Not serious | Not serious | Serious ^d^ | None | Positive direction. Significance NR. RR 26%. | ⨁⨁⨁◯  Moderate | (20) |
| 6 (6) | 1 controlled study,  5 uncontrolled studies | Serious ^b^ | Not serious | Not serious | Not serious | None | Positive direction in all six interventions. Significant difference in three, three NR. RR >= 20% in three interventions (three significant). RR range 12% - 96%. | ⨁◯◯◯  Very Low | (22, 26, 27, 30, 29, 28) |
| **Soft interventions: 25 (20)** | | | | | | | | |  |
| **Volume reduction** | | | | | | | | |  |
| 4 (2) | 2 cluster RCTs | Serious ^b^ | Not serious | Not serious | Not serious | None | Positive direction in all four interventions. Significant difference in two, one NR. RR range 10% - 18% | ⨁⨁⨁◯  Moderate | (31, 32) |
| 19 (16) | 5 controlled study,  11 uncontrolled studies | Serious ^b^ | Not serious | Not serious | Not serious | None | Positive direction in 17 interventions. Significant difference in nine, nine NR. RR >= 20% in nine interventions (four significant). RR <= -20% in both negative direction interventions (significance NR). RR range -32% - 89%. | ⨁◯◯◯  Very Low | (33–48) |
| **Improvement of care** | | | | | | | | |  |
| 1 (1) | 1 cluster RCT | Not Serious | Not serious | Not serious | Serious ^d^ | None | Positive direction. NS. RR 18%. | ⨁⨁⨁◯  Moderate | (20) |
| 10 (8) | 3 controlled study,  5 uncontrolled studies | Serious ^b^ | Not serious | Not serious | Not serious | None | Positive direction in all ten interventions. Significant difference in four, four NR. RR >= 20% in nine interventions (three significant). RR range 13% - 172%. | ⨁◯◯◯  Very Low | (36, 33, 35, 41, 44, 48–50) |

^a^ Multiple distinct interventions per study in two- and three-armed studies.

^b^ RCTs with potential selective outcome reporting bias. Observational studies with failure to adequately control confounding.(51)

^c^ Summary of effects based on results in data extraction table (Table 4 in manuscript). Positive percentage equals effect in positive direction.

^d^ Results based on single study or two studies, NS or significance NR.

**Abbreviations:** NS = Not significant, NR = Not Reported, RCT = Randomised Control Trial, RR = Relative Reduction (absolute value in cases of increasing aim).

Table 2: Summary of GRADE results by outcome. For explanations and abbreviations see table 1. All outcomes were considered to be of critical importance.

| **Certainty assessment** | | | | | | | **Effect ^c^** | **Certainty** | **References** |
| --- | --- | --- | --- | --- | --- | --- | --- | --- | --- |
| **No. of interventions (studies) ^a^** | **Study design** | **Risk of bias** | **Inconsistency** | **Indirectness** | **Imprecision** | **Other considerations** |  |  |  |
| **Improvement of care** | | | | | | | | |  |
| **Appropriateness** | | | | | | | | |  |
| 4 (2) | cluster RCT | Not serious | Not serious | Not serious | Serious ^d^ | None | Positive direction in all four interventions. Significant difference in two, one NR. RR >= 20% in two interventions (one significant). RR range 11% - 30%. | ⨁⨁⨁◯  Moderate | (21, 20) |
| **Volume reduction** | | | | | | | | |  |
| **Expenditure** | | | | | | | | |  |
| 1 (1) | RCT | Serious ^b^ | Not serious | Not serious | Serious ^d^ | None | Positive direction. NS. RR 10%. | ⨁⨁◯◯  Low | (3) |
| **Test numbers/rates** | | | | | | | | |  |
| 4 (2) | cluster RCT | Serious ^b^ | Not serious | Not serious | Not serious | None | Positive direction in all four interventions. Significant difference in two, one NR. RR range 10% -18%. | ⨁⨁⨁◯  Moderate | (32, 31) |
| **Improvement of care** | | | | | | | | |  |
| **Appropriateness** | | | | | | | | |  |
| 2 (1) | Controlled study | Serious ^b^ | Not serious | Not serious | Not serious | None | Positive direction in both interventions. Significant difference in one, one NR. RR range 52% - 172%. | ⨁◯◯◯  Very Low | (36) |
| **Coefficient of Variation** | | | | | | | | |  |
| 3 (1) | Controlled study | Serious ^b^ | Not serious | Not serious | Serious ^d^ | None | Positive direction in all three interventions. Significance NR. RR >= 20% in two interventions, RR range 13% - 38%. | ⨁◯◯◯  Very Low | (33) |
| **Pattern** | | | | | | | | |  |
| 2 (2) | Controlled study | Serious ^b^ | Not serious | Not serious | Not serious | None | Positive direction, significant difference and RR >= 20% in both interventions. RR range 4% - 86%. | ⨁◯◯◯  Very Low | (26, 35) |
| **Volume reduction** | | | | | | | | |  |
| **Test numbers/rates** | | | | | | | | |  |
| 12 (9) | Controlled study | Serious ^b^ | Not serious | Not serious | Not serious | None | Positive direction in 11 interventions. Significant difference in five, five NR. RR >= 20% in two interventions (one significant). Negative direction in one intervention with  RR < -20%, significance NR. RR range -31% - 44%. | ⨁◯◯◯  Very Low | (4, 7, 34, 26, 5, 36, 33, 35, 39) |
| Expenditure |  |  |  |  |  |  |  |  |  |
| 1 (1) | Controlled study | Serious ^b^ | Not serious | Not serious | Serious ^d^ | None | Positive direction, RR 20%, significance NR | ⨁◯◯◯  Very Low | (35) |
| **Improvement of care** | | | | | | | | |  |
| **Appropriateness** | | | | | | | | |  |
| 8 (8) | Uncontrolled study | Serious ^b^ | Not serious | Not serious | Not serious | None | Positive direction in all eight interventions. Significant difference in three, three NR. RR >= 20% in six interventions (one significant). RR range 7% - 96%. | ⨁◯◯◯  Very Low | (22, 11, 15, 30, 16, 48, 44, 41) |
| **Pattern** | | | | | | | | |  |
| 6 (6) | Uncontrolled study | Serious ^b^ | Not serious | Not serious | Not serious | None | Positive direction in all six interventions. Significant difference in three, three NR. RR >= 20% in five interventions (two significant). RR range 9% - 96%. | ⨁◯◯◯  Very Low | (27–29, 50, 49, 9, 48) |
| **Volume reduction** | | | | | | | | |  |
| **Expenditure** | | | | | | | | |  |
| 7 (7) | Serious ^b^ | Serious ^b^ | Not serious | Not serious | Not serious | None | Positive direction in all seven interventions. Significant difference in two, five NR. RR >= 20% in five interventions (one significant). RR range 14% - 89%. | ⨁◯◯◯  Very Low | (22, 38, 37, 19, 15, 27, 42) |
| **Test numbers/rates** | | | | | | | | |  |
| 28 (28) | Uncontrolled study | Serious ^b^ | Not serious | Not serious | Not serious | None | Positive direction in 27 interventions. Significant difference in 15, 11 NR. RR >= 20% in 23 interventions (15 significant). Negative direction in one intervention with RR < -20%, significant. RR range -20% - 96%. | ⨁◯◯◯  Very Low | (23, 24, 8, 12, 11, 10, 14, 13, 17, 18, 47, 37, 25, 19, 15, 28, 29, 27, 9, 6, 16, 41, 40, 45, 43, 44, 46, 48) |

**Abbreviations:** NS = Not significant, NR = Not Reported, RCT = Randomised Control Trial, RR = Relative Reduction (absolute value in cases of increasing aim).

**Literature Cited**

1. Murad MH, Almasri J, Alsawas M, Farah W. Grading the quality of evidence in complex interventions: a guide for evidence-based practitioners. Evid Based Med 2017; 22(1):20–2.

2. Guyatt G, Oxman AD, Akl EA, Kunz R, Vist G, Brozek J et al. GRADE guidelines: 1. Introduction-GRADE evidence profiles and summary of findings tables. Journal of clinical epidemiology 2011; 64(4):383–94.

3. Tierney WM, McDonald CJ, Hui SL, Martin DK. Computer predictions of abnormal test results. Effects on outpatient testing. J. Am. Med. Assoc. 1988; 259(8):1194–8.

4. Chami N, Li Y, Weir S, Wright JG, Kantarevic J. Effect of Strict and Soft Policy Interventions on Laboratory Diagnostic Testing in Ontario, Canada: A Bayesian Structural Time Series Analysis. Health policy 2021; 125(2):254–60.

5. Horn DM, Koplan KE, Senese MD, Orav EJ, Sequist TD. The impact of cost displays on primary care physician laboratory test ordering. Journal of general internal medicine 2014; 29(5):708–14.

6. Chu KH, Wagholikar AS, Greenslade JH, O'Dwyer JA, Brown AF. Sustained reductions in emergency department laboratory test orders: Impact of a simple intervention. Postgrad. Med. J. 2013; 89(1056):566–71.

7. Bellodi E, Vagnoni E, Bonvento B, Lamma E. Economic and organizational impact of a clinical decision support system on laboratory test ordering. BMC medical informatics and decision making 2017; 17(1):179.

8. Dalal S, Bhesania S, Silber S, Mehta P. Use of electronic clinical decision support and hard stops to decrease unnecessary thyroid function testing. BMJ Open Qual. 2017; 6(1):u223041. w8346.

9. Emerson JF, Emerson SS. The impact of requisition design on laboratory utilization. AM. J. CLIN. PATHOL. 2001; 116(6):879–84.

10. Krouss M, Israilov S, Alaiev D, Hupart K, Da Shin W, Mestari N et al. Free the T3: implementation of best practice advisory to reduce unnecessary orders. The American journal of medicine 2022; 135(12):1437–42.

11. Leis B, Frost A, Bryce R, Lyon AW, Coverett K. Altering standard admission order sets to promote clinical laboratory stewardship: A cohort quality improvement study. BMJ Qual. Saf. 2019; 28(10):846–52.

12. Muris DMJ, Molenaers M, Nguyen T, Bergmans, P. W. M. P., van Acker BAC, Krekels MME et al. Effect of a price display intervention on laboratory test ordering behavior of general practitioners. BMC Fam. Pract. 2021; 22(1).

13. Notas G, Kampa M, Malliaraki N, Petrodaskalaki M, Papavasileiou S, Castanas E. Implementation of thyroid function tests algorithms by clinical laboratories: A four-year experience of good clinical and diagnostic practice in a tertiary hospital in Greece. Eur. J. Intern. Med. 2018; 54:81–6.

14. Sue LY, Kim JE, Oza H, Chong T, Woo HE, Cheng EM et al. Reducing Inappropriate Serum T3 Laboratory Test Ordering in Patients with Treated Hypothyroidism. Endocr. Pract. 2019; 25(12):1312–6.

15. Caldarelli G, Troiano G, Rosadini D, Nante N. Adoption of TSH Reflex algorithm in an Italian clinical laboratory. Annali di igiene : medicina preventiva e di comunita 2017; 29(4):317–22.

16. Feldkamp CS, Carey JL. An algorithmic approach to thyroid function testing in a managed care setting: 3-Year experience. AM. J. CLIN. PATHOL. 1996; 105(1):11–6.

17. Salinas M, López-Garrigós M, Flores E, Leiva-Salinas M, Asencio A, Lugo J et al. Managing inappropriate requests of laboratory tests: From detection to monitoring. Am. J. Managed Care 2016; 22(9):e311-e316.

18. Taher J, Beriault DR, Yip D, Tahir S, Hicks LK, Gilmour JA. Reducing free thyroid hormone testing through multiple Plan-Do-Study-Act cycles. Clin. Biochem. 2020; 81:41–6.

19. Bejjanki H, Mramba LK, Beal SG, Radhakrishnan N, Bishnoi R, Shah C et al. The role of a best practice alert in the electronic medical record in reducing repetitive lab tests. ClinicoEconomics and outcomes research : CEOR 2018; 10:611–8.

20. Daucourt V, Saillour-Glénisson F, Michel P, Jutand MA, Abouelfath A. A multicenter cluster randomized controlled trial of strategies to improve thyroid function testing. Med. Care 2003; 41(3):432–41.

21. Delvaux N, Piessens V, Burghgraeve T de, Mamouris P, Vaes B, Stichele RV et al. Clinical decision support improves the appropriateness of laboratory test ordering in primary care without increasing diagnostic error: the ELMO cluster randomized trial. Implementation science : IS 2020; 15(1):100.

22. Elrewini AM, Zubair M, Afridi NK, Dildar MT, Javed H, Alwalah SM. To determine the effectiveness of different interventions to reduce unnecessary requests of serum thyroid stimulating hormone levels in a hospital. The Professional Medical Journal 2022; 29(05):686–92.

23. Gilmour JA, Weisman A, Orlov S, Goldberg RJ, Goldberg A, Baranek H et al. Promoting resource stewardship: Reducing inappropriate free thyroid hormone testing. J. Eval. Clin. Pract. 2017; 23(3):670–5.

24. Bradshaw AB, Bonnecaze AK, Burns CA, Beardsley JR. Impact of an Interprofessional Collaborative Quality Improvement Initiative to Decrease Inappropriate Thyroid Function Testing. Hosp. Pharm. 2021; 56(5):481–5.

25. MacPherson RD, Reeve SA, Stewart TV, Cunningham AES, Craven ML, Fox G et al. Effective strategy to guide pathology test ordering in surgical patients. ANZ journal of surgery 2005; 75(3):138–43.

26. Wong ET, McCarron MM, Shaw ST. Ordering of Laboratory Tests in a Teaching Hospital: Can It Be Improved? JAMA 1983; 249(22):3076–80.

27. Hardwick DF, Morrison JI, Tydeman J, Cassidy PA, Chase WH. Structuring complexity of testing: a process oriented approach to limiting unnecessary laboratory use. The American journal of medical technology 1982; 48 7:605–8.

28. van Walraven C, Goel V, Chan B. Effect of population-based interventions on laboratory utilization: A time-series analysis. J. Am. Med. Assoc. 1998; 280(23):2028–33.

29. Vidal-Trécan G, Toubert ME, Coste J, Paycha F, Durand-Zaleski I, Fulla Y et al. Reducing the number of T3 orders in the Paris hospital network: Towards better appropriatness of thyroid function test prescription. Ann. Endocrinol. 2003; 64(3):210–5.

30. Nightingale PG, Peters M, Mutimer D, Neuberger JM. Effects of a computerised protocol management system on ordering of clinical tests. Quality in health care : QHC 1994; 3(1):23–8.

31. Baker R, Smith JF, Lambert PC. Randomised controlled trial of the effectiveness of feedback in improving test ordering in general practice. Scand. J. Prim. Health Care 2003; 21(4):219–23.

32. Thomas RE, Croal BL, Ramsay C, Eccles M, Grimshaw J. Effect of enhanced feedback and brief educational reminder messages on laboratory test requesting in primary care: a cluster randomised trial. Lancet 2006; 367(9527):1990–6.

33. Berwick DM, Coltin KL. Feedback reduces test use in a health maintenance organization. J. Am. Med. Assoc. 1986; 255(11):1450–4.

34. Wintemute K, Greiver M, McIsaac W, Del Elisabeth Giudice M, Sullivan F, Aliarzadeh B et al. Choosing Wisely Canada campaign associated with less overuse of thyroid testing Retrospective parallel cohort study. Can. Fam. Phys. 2019; 65(11):E487-E496.

35. Tomlin A, Dovey S, Gauld R, Tilyard M. Better use of primary care laboratory services following interventions to 'market' clinical guidelines in New Zealand: A controlled before-and-after study. BMJ Qual. Saf. 2011; 20(3):282–90.

36. Schectman JM, Elinsky EG, Pawlson LG. Effect of Education and Feedback on Thyroid Function Testing Strategies of Primary Care Clinicians. Arch. Intern. Med. 1991; 151(11):2163–6.

37. Janssens PMW, Staring W, Winkelman K, Krist G. Active intervention in hospital test request panels pays. Clinical chemistry and laboratory medicine 2015; 53(5):731–42.

38. Leung E, Song S, Al-Abboud O, Shams S, English J, Naji W et al. An educational intervention to increase awareness reduces unnecessary laboratory testing in an internal medicine resident-run clinic. Journal of community hospital internal medicine perspectives 2017; 7(3):168–72.

39. Gama R, Nightingale PG, Broughton PM, Peters M, Bradby GV, Berg J et al. Feedback of laboratory usage and cost data to clinicians: does it alter requesting behaviour? Annals of clinical biochemistry 1991; 28 (Pt 2):143–9.

40. Grivell AR, Forgie HJ, Fraser CG, Berry MN. Effect of feedback to clinical staff of information on clinical biochemistry requesting patterns. Clinical chemistry 1981; 27(10):1717–20.

41. Dowling PT, Alfonsi G, Brown MI, Culpepper L. An education program to reduce unnecessary laboratory tests by residents. J. Med. Educ. 1989; 64(7):410–2.

42. Stuart PJ, Crooks S, Porton M. An interventional program for diagnostic testing in the emergency department. The Medical journal of Australia 2002; 177(3):131–4.

43. Adlan MA, Neel V, Lakra SS, Bondugulapati LNR, Premawardhana, L. D. K. E. Targeted thyroid testing in acute illness: Achieving success through audit. J. Endocrinol. Invest. 2011; 34(8 SUPPL.):e210-e213.

44. Rhyne RL, Gehlbach SH. Effects of an educational feedback strategy on physician utilization of thyroid function panels. The Journal of family practice 1979; 8(5):1003–7.

45. Willis EA, Datta BN. Effect of an educational intervention on requesting behaviour by a medical admission unit. Ann. Clin. Biochem. 2013; 50(2):166–8.

46. Cipullo JA, Mostoufizadeh M. Bringing order to test orders: one lab's story. CAP today 1996; 10(1):20–2.

47. Bateman EA, Gob A, Chin-Yee I, MacKenzie HM. Reducing waste: A guidelines-based approach to reducing inappropriate Vitamin D and TSH testing in the inpatient rehabilitation setting. BMJ Open Qual. 2019; 8(4).

48. Toubert ME, Chevret S, Cassinat B, Schlageter MH, Beressi JP, Rain JD. From guidelines to hospital practice: Reducing inappropriate ordering of thyroid hormone and antibody tests. Eur. J. Endocrinol. 2000; 142(6):605–10.

49. Larsson A, Biom S, Wernroth ML, Hultén G, Tryding N. Effects of an education programme to change clinical laboratory testing habits in primary care. Scandinavian journal of primary health care 1999; 17(4):238–43.

50. Mindemark M, Larsson A. Long-term effects of an education programme on the optimal use of clinical chemistry testing in primary health care. Scandinavian journal of clinical and laboratory investigation 2009; 69(4):481–6.

51. Guyatt GH, Oxman AD, Vist G, Kunz R, Brozek J, Alonso-Coello P et al. GRADE guidelines: 4. Rating the quality of evidence--study limitations (risk of bias). Journal of clinical epidemiology 2011; 64(4):407–15.
