# Supplementary material for: Effectiveness and mechanisms of interventions to reduce low-value thyroid function tests: a systematic review
Source: Syst Rev. 2026 Feb 25;15:111. doi: 10.1186/s13643-026-03119-8 (PMC13040701; doi:10.1186/s13643-026-03119-8)
Supplement: Supplementary file 8 — Additional file 8. Additional file 8 includes the information on all articles excluded after full-text screening, including reason for exclusion. [file 13643_2026_3119_MOESM8_ESM.docx]

**Excluded references**

| **No.** | **Reference** | **Reason for exclusion** |
| --- | --- | --- |
| **Previous Studies from Zhelev et al. (n = 1)** | | |
| 1 | J. M. van Gend; J. van Pelt; T. H. Cleef; T. M. Mangnus; J. W. Muris. "Kwaliteitsverbeteringsproject 'laboratoriumdiagnostiek door huisartsen' leidt tot aanzienlijke reductie van het aantal laboratoriumanalysen". 1996. Nederlands tijdschrift voor geneeskunde. | Language |
| **Identification through literature search: studies via databases and registers (n = 71 of which n = 2 not retrieved)** | | |
| 2 | E. Colussi-Pelaez; R. Fine; R. Fung; Z. Lysy. "Determining if the methods put in place to reduce unnecessary free thyroid testing at Michael Garron Hospital have been successful". 2020. BMC Proceedings. https://dx.doi.org/10.1186/s12919-020-00185-1. | Conference Abstract |
| 3 | M. Esen; H. B. Yavuz. "Restriction of the test requesting frequency to avoiding unnecessary testing". 2017. Turkish Journal of Biochemistry. | Conference Abstract |
| 4 | J. Gilmour; A. Weisman; S. Orlov; J. Vecchiarelli; A. Goldberg; R. Goldberg; G. Mukerji. "Reducing unnecessary free thyroid hormone testing at an academic ambulatory hospital: A quality improvement (QI) initiative". 2015. Thyroid. https://dx.doi.org/10.1089/thy.2015.29004.abstracts. | Conference Abstract |
| 5 | J. Gilmour; A. Weisman; J. Vecchiarelli; S. Orlov; R. J. Goldberg; A. Goldberg. "Reducing inappropriate thyroid function tests at an academic ambulatory hospital: Baseline assessment for a quality improvement initiative". 2015. Endocrine Reviews. | Conference Abstract |
| 6 | A. Pema; O. Kiabilua; T. Pillay. "How effective is electronic gate-keeping in influencing test requesting behaviour and cost saving: A re-evaluation". 2017. Clinical Chemistry and Laboratory Medicine. https://dx.doi.org/10.1515/cclm-2017-5025. | Conference Abstract |
| 7 | M. Salinas; M. Lopez-Garrigos; E. Flores; M. Gutierrez; J. Lugo; A. Asencio. "Managing laboratory tests over request: Five year evaluation through indicators". 2015. Clinical Chemistry and Laboratory Medicine. | Conference Abstract |
| 8 | J. Taher; J. Gilmour; L. K. Hicks; D. Brinc; D. Beriault. "Do I really need fT3 and fT4? An overutilization change strategy to reduce free thyroid hormone testing". 2019. Clinical Biochemistry. https://dx.doi.org/10.1016/j.clinbiochem.2019.11.008. | Conference Abstract |
| 9 | A. A. Venner; S. H. Sadrzadeh. "Potential impact of TSH algorithm testing on thyroid hormone ordering". 2019. Clinica Chimica Acta. https://dx.doi.org/10.1016/j.cca.2019.03.723. | Conference Abstract |
| 10 | W. Wan; J. Wardian; W. Forbes; J. Kluesner; J. Tate; D. Beckman; S. Graybill; M. Kravchenko. "Reducing unnecessary thyroid function testing through systemic changes in ordering practices". 2020. Endocrine Practice. | Conference Abstract |
| 11 | N. Chami; S. Mathew; S. Weir; J. G. Wright; J. Kantarevic. "Adoption of a laboratory EMR system and inappropriate laboratory testing in Ontario: a cross-sectional observational study". 2021. BMC Health Services Research. 10.1186/s12913-021-06296-5. | Different aim |
| 12 | A. F. Finn; P. N. Valenstein; M. D. Burke. "Alteration of Physicians’ Orders by Nonphysicians". 1988. JAMA: The Journal of the American Medical Association. 10.1001/jama.1988.03720170025027. | Different aim |
| 13 | L. K. Hicks; P. O'Brien; M. Sholzberg; N. Veloce; A. Trafford; D. Sinclair. "Tackling overutilization of hospital tests and treatments: Lessons learned from a grassroots approach". 2018. Healthcare management forum. https://dx.doi.org/10.1177/0840470418781172. | Different aim |
| 14 | S. Morgan; A. Morgan; R. Kerr; A. Tapley; S. P. Magin. "Test ordering by GP trainees Effects of an educational intervention on attitudes and intended practice". 2016. Canadian Family Physician. | Different aim |
| 15 | A. K. Pema; O. Kiabilua; T. S. Pillay. "Demand management by electronic gatekeeping of test requests does not influence requesting behaviour or save costs dramatically". 2018. Annals of Clinical Biochemistry. https://dx.doi.org/10.1177/0004563217707980. | Different aim |
| 16 | M. A. Adlan; V. Neel; S. S. Lakra; L. N. R. Bondugulapati; L. D. K. E. Premawardhana. "Targeted thyroid testing in acute illness: Achieving success through audit". 2011. Journal of Endocrinological Investigation. 10.3275/7480. | Included in previous review |
| 17 | R. Baker; J. F. Smith; P. C. Lambert. "Randomised controlled trial of the effectiveness of feedback in improving test ordering in general practice". 2003. Scandinavian Journal of Primary Health Care. 10.1080/02813430310002995. | Included in previous review |
| 18 | K. H. Chu; A. S. Wagholikar; J. H. Greenslade; J. A. O'Dwyer; A. F. Brown. "Sustained reductions in emergency department laboratory test orders: Impact of a simple intervention". 2013. Postgraduate Medical Journal. 10.1136/postgradmedj-2012-130833. | Included in previous review |
| 19 | V. Daucourt; F. Saillour-Glénisson; P. Michel; M. A. Jutand; A. Abouelfath. "A multicenter cluster randomized controlled trial of strategies to improve thyroid function testing". 2003. Medical Care. 10.1097/01.MLR.0000053216.33277.A4. | Included in previous review |
| 20 | P. T. Dowling; G. Alfonsi; M. I. Brown; L. Culpepper. "An education program to reduce unnecessary laboratory tests by residents". 1989. Journal of Medical Education. 10.1097/00001888-198907000-00017. | Included in previous review |
| 21 | J. F. Emerson; S. S. Emerson. "The impact of requisition design on laboratory utilization". 2001. American Journal of Clinical Pathology. 10.1309/WC83-ERLY-NEDF-471E. | Included in previous review |
| 22 | C. S. Feldkamp; J. L. Carey. "An algorithmic approach to thyroid function testing in a managed care setting: 3-Year experience". 1996. American Journal of Clinical Pathology. 10.1093/ajcp/105.1.11. | Included in previous review |
| 23 | D.F. Hardwick; J.I. Morrison; J. Tydema; P.A. Cassidy; W.H. Chase. “Structuring complexity of testing: a process oriented approach to limiting unnecessary laboratory use.” 1982. The American journal of medical technology. | Included in previous review |
| 24 | D. M. Horn; K. E. Koplan; M. D. Senese; E. J. Orav; T. D. Sequist. "The impact of cost displays on primary care physician laboratory test ordering". 2014. Journal of general internal medicine. https://dx.doi.org/10.1007/s11606-013-2672-1. | Included in previous review |
| 25 | J. M. Schectman; E. G. Elinsky; L. G. Pawlson. "Effect of Education and Feedback on Thyroid Function Testing Strategies of Primary Care Clinicians". 1991. Archives of Internal Medicine. 10.1001/archinte.1991.00400110027006. | Included in previous review |
| 26 | A. Tomlin; S. Dovey; R. Gauld; M. Tilyard. "Better use of primary care laboratory services following interventions to 'market' clinical guidelines in New Zealand: A controlled before-and-after study". 2011. BMJ Quality and Safety. 10.1136/bmjqs.2010.048124. | Included in previous review |
| 27 | M. E. Toubert; S. Chevret; B. Cassinat; M. H. Schlageter; J. P. Beressi; J. D. Rain. "From guidelines to hospital practice: Reducing inappropriate ordering of thyroid hormone and antibody tests". 2000. European Journal of Endocrinology. 10.1530/eje.0.1420605. | Included in previous review |
| 28 | C. Van Walraven; V. Goel; B. Chan. "Effect of population-based interventions on laboratory utilization: A time-series analysis". 1998. JAMA. 10.1001/jama.280.23.2028. | Included in previous review |
| 29 | G. Vidal-Trécan; M. E. Toubert; J. Coste; F. Paycha; I. Durand-Zaleski; Y. Fulla; A. Abella; R. Fior; P. Georges. "Reducing the number of T3 orders in the Paris hospital network: Towards better appropriatness of thyroid function test prescription". 2003. Annales d'Endocrinologie. | Included in previous review |
| 30 | E. A. Willis; B. N. Datta. "Effect of an educational intervention on requesting behaviour by a medical admission unit". 2013. Annals of Clinical Biochemistry. 10.1258/acb.2012.012100. | Included in previous review |
| 31 | E. T. Wong; M. M. McCarron; S. T. Shaw. "Ordering of Laboratory Tests in a Teaching Hospital: Can It Be Improved?". 1983. JAMA: The Journal of the American Medical Association. 10.1001/jama.1983.03330460058036. | Included in previous review |
| 32 | F. Saillour-Glénisson; P. Michel; V. Daucourt. "Medico-economic assessment of two methods for implementing thyroid testing guidelines". 2005. Revue d'Epidemiologie et de Sante Publique. | Language |
| 33 | A. Ambasta; I. W. Y. Ma; S. Woo; K. Lonergan; E. Mackay; T. Williamson. "Impact of an education and multilevel social comparison-based intervention bundle on use of routine blood tests in hospitalised patients at an academic tertiary care hospital: a controlled pre-intervention post-intervention study". 2020. BMJ quality & safety. 10.1136/bmjqs-2019-010118. | No (separate) TFT reporting |
| 34 | A. Ambasta; O. Omodon; A. Herring; L. Ferrie; S. Pokharel; A. Mehta; L. Liu; J. Hews-Girard; C. Tam; S. Taylor; K. Lonergan; P. Faris; D. Duncan; D. Woodhouse. "Repurposing the Ordering of Routine Laboratory Tests in Hospitalised Medical Patients (RePORT): results of a cluster randomised stepped-wedge quality improvement study". 2023. BMJ quality & safety. https://dx.doi.org/10.1136/bmjqs-2022-015611. | No (separate) TFT reporting |
| 35 | R. S. Bindraban; M. van Beneden; M. H. H. Kramer; W. W. van Solinge; P. M. van de Ven; C. A. Naaktgeboren; M. Al-Dulaimy; L. C. van der Wekken; Y. C. Bandt; F. Stam; S. I. M. Neppelenbroek; A. Griffioen-Keijzer; D. A. R. Castelijn; B. A. Wevers; A. W. Boerman; M. van Wijnen; M. J. Ten Berg; P. W. B. Nanayakkara. "Association of a Multifaceted Intervention With Ordering of Unnecessary Laboratory Tests Among Caregivers in Internal Medicine Departments". 2019. JAMA network open. https://dx.doi.org/10.1001/jamanetworkopen.2019.7577. | No (separate) TFT reporting |
| 36 | E. Chang; D. S. M. Buist; M. Handley; E. Johnson; S. Fuller; R. Pardee; G. Gundersen; R. J. Reid. "Primary care physician resource use changes associated with feedback reports". 2018. The American journal of managed care. | No (separate) TFT reporting |
| 37 | B. Clouzeau; M. Caujolle; A. San-Miguel; J. Pillot; N. Gazeau; C. Tacaille; V. Dousset; F. Bazin; F. Vargas; G. Hilbert; M. Molimard; D. Gruson; A. Boyer. "The sustainable impact of an educational approach to improve the appropriateness of laboratory test orders in the ICU". 2019. PloS one. https://dx.doi.org/10.1371/journal.pone.0214802. | No (separate) TFT reporting |
| 38 | K. Ekblom; A. Petersson. "Introduction of cost display reduces laboratory test utilization". 2018. The American journal of managed care. | No (separate) TFT reporting |
| 39 | M. O. Elnenaei; S. G. Campbell; A. J. Thoni; A. Lou; B. D. Crocker; B. A. Nassar. "An effective utilization management strategy by dual approach of influencing physician ordering and gate keeping". 2016. Clinical biochemistry. https://dx.doi.org/10.1016/j.clinbiochem.2015.11.005. | No (separate) TFT reporting |
| 40 | D. Giordano; M. Zasa; C. Iaccarino; V. Vincenti; I. Dascola; B. C. Brevi; T. Gherli; M. G. Raso; G. Campaniello; P. Bonelli; A. Vezzani. "Improving laboratory test ordering can reduce costs in surgical wards". 2015. Acta bio-medica : Atenei Parmensis. | No (separate) TFT reporting |
| 41 | M. B. Greenblatt; J. A. Nowak; C. C. Quade; M. Tanasijevic; N. Lindeman; P. Jarolim. "Impact of a prospective review program for reference laboratory testing requests". 2015. American journal of clinical pathology. https://dx.doi.org/10.1309/AJCPN1VCZDVD9ZVX. | No (separate) TFT reporting |
| 42 | Y. Hirota; S. Suzuki; Y. Ohira; K. Shikino; M. Ikusaka. "The Effectiveness of Cost Reduction with Charge Displays on Test Ordering under the Health Insurance System in Japan: a Study Using Paper-based Simulated Cases for Residents and Clinical Fellows". 2019. Internal medicine (Tokyo, Japan). 10.2169/internalmedicine.0738-17. | No (separate) TFT reporting |
| 43 | E. Iturrate; L. Jubelt; F. Volpicelli; K. Hochman. "Optimize Your Electronic Medical Record to Increase Value: Reducing Laboratory Overutilization". 2016. The American journal of medicine. https://dx.doi.org/10.1016/j.amjmed.2015.09.009. | No (separate) TFT reporting |
| 44 | N. Kotecha; J. M. Shapiro; J. Cardasis; G. Narayanswami. "Reducing Unnecessary Laboratory Testing in the Medical ICU". 2017. The American journal of medicine. https://dx.doi.org/10.1016/j.amjmed.2017.02.014. | No (separate) TFT reporting |
| 45 | D. E. Kurant; J. M. Baron; G. Strazimiri; K. B. Lewandrowski; J. W. Rudolf; A. S. Dighe. "Creation and Use of an Electronic Health Record Reporting Database to Improve a Laboratory Test Utilization Program". 2018. Applied clinical informatics. https://dx.doi.org/10.1055/s-0038-1666843. | No (separate) TFT reporting |
| 46 | E. Litton; H. Atkinson; J. Anstey; M. Anstey; L. T. Campbell; A. Forbes; R. Hahn; K. Hooper; J. Kasza; S. Knapp; F. McGain; N. Ngyuen; D. Pilcher; B. Reddi; C. Reid; S. Robinson; K. Thompson; S. Webb; P. Young. "Optimising a targeted test reduction intervention for patients admitted to the intensive care unit: The Targeted Intensive Care Test Ordering Cluster Trial intervention". 2021. Australian critical care : official journal of the Confederation of Australian Critical Care Nurses. https://dx.doi.org/10.1016/j.aucc.2020.11.003. | No (separate) TFT reporting |
| 47 | C. M. S. Martins; A. S. da Costa Teixeira; L. F. R. de Azevedo; L. M. B. Sa; P. A. A. P. Santos; M. L. G. D. do Couto; A. M. R. da Costa Pereira; A. A. O. P. Hespanhol; C. M. N. da Costa Santos. "The effect of a test ordering software intervention on the prescription of unnecessary laboratory tests - a randomized controlled trial". 2017. BMC medical informatics and decision making. https://dx.doi.org/10.1186/s12911-017-0416-6. | No (separate) TFT reporting |
| 48 | L. May; P. Franks; A. Jerant; J. Fenton. "Watchful Waiting Strategy May Reduce Low-Value Diagnostic Testing". 2016. Journal of the American Board of Family Medicine : JABFM. https://dx.doi.org/10.3122/jabfm.2016.06.160056. | No (separate) TFT reporting |
| 49 | E. G. McDonald; R. R. Saleh; T. C. Lee. "Mindfulness-Based Laboratory Reduction: Reducing Utilization Through Trainee-Led Daily 'Time Outs'". 2017. The American journal of medicine. https://dx.doi.org/10.1016/j.amjmed.2017.01.011. | No (separate) TFT reporting |
| 50 | C. Minerowicz; N. Abel; K. Hunter; K. C. Behling; E. Cerceo; C. Bierl. "Impact of weekly feedback on test ordering patterns". 2015. The American journal of managed care. | No (separate) TFT reporting |
| 51 | P. Petrou. "Failed Attempts to Reduce Inappropriate Laboratory Utilization in an Emergency Department Setting in Cyprus: Lessons Learned". 2016. The Journal of emergency medicine. https://dx.doi.org/10.1016/j.jemermed.2015.07.025. | No (separate) TFT reporting |
| 52 | G. W. Procop; C. Keating; P. Stagno; K. Kottke-Marchant; M. Partin; R. Tuttle; R. Wyllie. "Reducing duplicate testing: a comparison of two clinical decision support tools". 2015. American journal of clinical pathology. https://dx.doi.org/10.1309/AJCPJOJ3HKEBD3TU. | No (separate) TFT reporting |
| 53 | S. Raad; R. Elliott; E. Dickerson; B. Khan; K. Diab. "Reduction of Laboratory Utilization in the Intensive Care Unit". 2017. Journal of intensive care medicine. https://dx.doi.org/10.1177/0885066616651806. | No (separate) TFT reporting |
| 54 | J. D. Riley; G. Stanley; R. Wyllie; K. Kottke-Marchant; G. W. Procop. "The Impact of an Electronic Expensive Test Notification". 2018. American journal of clinical pathology. https://dx.doi.org/10.1093/ajcp/aqy021. | No (separate) TFT reporting |
| 55 | M. Salinas; M. Lopez-Garrigos; E. Flores; M. Leiva-Salinas; P. Esteban; M. Ahumada; C. Leiva-Salinas. "Indications for laboratory tests in primary care: assessment of the most frequent indications and requests with blank clinical information". 2016. Biochemia medica. | No (separate) TFT reporting |
| 56 | M. S. Sedrak; J. S. Myers; D. S. Small; I. Nachamkin; J. B. Ziemba; D. Murray; G. W. Kurtzman; J. Zhu; W. Wang; D. Mincarelli; D. Danoski; B. P. Wells; J. S. Berns; P. J. Brennan; C. W. Hanson; C. J. Dine; M. S. Patel. "Effect of a Price Transparency Intervention in the Electronic Health Record on Clinician Ordering of Inpatient Laboratory Tests: The PRICE Randomized Clinical Trial". 2017. JAMA internal medicine. https://dx.doi.org/10.1001/jamainternmed.2017.1144. | No (separate) TFT reporting |
| 57 | M. Shirazi; Y. Masoudian; E. Feizabad; F. Golshahi; M. Ghaemi. "Creating a novel strategy to reduce unnecessary laboratory testing based on healthcare cost analysis in high-risk pregnancies and delivery ward". 2021. Journal of clinical laboratory analysis. https://dx.doi.org/10.1002/jcla.23759. | No (separate) TFT reporting |
| 58 | A. Sorita; D. I. Steinberg; M. Leitman; A. Burger; G. Husk; L. Sivaprasad. "The assessment of stat laboratory test ordering practice and impact of targeted individual feedback in an urban teaching hospital". 2014. Journal of hospital medicine. https://dx.doi.org/10.1002/jhm.2108. | No (separate) TFT reporting |
| 59 | R. N. Thakkar; D. Kim; A. M. Knight; S. Riedel; D. Vaidya; S. M. Wright. "Impact of an educational intervention on the frequency of daily blood test orders for hospitalized patients". 2015. American journal of clinical pathology. https://dx.doi.org/10.1309/AJCPJS4EEM7UAUBV. | No (separate) TFT reporting |
| 60 | B. M. Wertheim; A. J. Aguirre; R. P. Bhattacharyya; J. Chorba; A. P. Jadhav; V. B. Kerry; E. A. Macklin; G. Motyckova; S. Raju; K. Lewandrowski; D. P. Hunt; D. E. Wright. "An Educational and Administrative Intervention to Promote Rational Laboratory Test Ordering on an Academic General Medicine Service". 2017. The American journal of medicine. https://dx.doi.org/10.1016/j.amjmed.2016.08.021. | No (separate) TFT reporting |
| 61 | E. J. Wiens; I. Supel; J. Gallardo; C. M. Seifer. "Signage as an intervention on a general medicine ward to reduce unnecessary testing". 2021. Internal medicine journal. https://dx.doi.org/10.1111/imj.14784. | No (separate) TFT reporting |
| 62 | D.A. O'Connor; D. Schram. "Feedback for reducing overuse of pathology test requesting by Australian general practitioners". 2022. https://trialsearch.who.int/Trial2.aspx?TrialID=ACTRN12622000566730. | No primary study |
| 63 | R. S. Bindraban; M. J. Ten Berg; C. A. Naaktgeboren; M. H. H. Kramer; W. W. Van Solinge; P. W. B. Nanayakkara. "Reducing Test Utilization in Hospital Settings: A Narrative Review". 2018. Annals of laboratory medicine. https://dx.doi.org/10.3343/alm.2018.38.5.402. | No primary study |
| 64 | N. Delvaux; K. Van Thienen; A. Heselmans; S. V. de Velde; D. Ramaekers; B. Aertgeerts. "The Effects of Computerized Clinical Decision Support Systems on Laboratory Test Ordering: A Systematic Review". 2017. Archives of pathology & laboratory medicine. https://dx.doi.org/10.5858/arpa.2016-0115-RA. | No primary study |
| 65 | K. P. Hooper; M. H. Anstey; E. Litton. "Safety and efficacy of routine diagnostic test reduction interventions in patients admitted to the intensive care unit: A systematic review and meta-analysis". 2021. Anaesthesia and intensive care. https://dx.doi.org/10.1177/0310057X20962113. | No primary study |
| 66 | M. Rubinstein; R. Hirsch; K. Bandyopadhyay; B. Madison; T. Taylor; A. Ranne; M. Linville; K. Donaldson; F. Lacbawan; N. Cornish. "Effectiveness of Practices to Support Appropriate Laboratory Test Utilization: A Laboratory Medicine Best Practices Systematic Review and Meta-Analysis". 2018. American journal of clinical pathology. https://dx.doi.org/10.1093/ajcp/aqx147. | No primary study |
| 67 | R. E. Thomas; M. Vaska; C. Naugler; T. C. Turin. "Interventions at the laboratory level to reduce laboratory test ordering by family physicians: Systematic review". 2015. Clinical biochemistry. https://dx.doi.org/10.1016/j.clinbiochem.2015.09.014. | No primary study |
| 68 | B. Yeshoua; C. Bowman; J. Dullea; J. Ditkowsky; M. Shyu; H. Lam; W. Zhao; J. Y. Shin; A. Dunn; S. Tsega; A. S Linker; M. Shah. "Interventions to reduce repetitive ordering of low-value inpatient laboratory tests: a systematic review". 2023. BMJ open quality. https://dx.doi.org/10.1136/bmjoq-2022-002128. | No primary study |
| 69 | S. Zare; Z. Meidani; M. Shirdeli; E. Nabovati. "Laboratory test ordering in inpatient hospitals: a systematic review on the effects and features of clinical decision support systems". 2021. BMC medical informatics and decision making. https://dx.doi.org/10.1186/s12911-020-01384-8. | No primary study |
| 70 | Z. Zhelev; R. Abbott; M. Rogers; S. Fleming; A. Patterson; W. T. Hamilton; J. Heaton; J. Thompson Coon; B. Vaidya; C. Hyde. "Effectiveness of interventions to reduce ordering of thyroid function tests: a systematic review". 2016. BMJ open. https://dx.doi.org/10.1136/bmjopen-2015-010065. | No primary study |
| 71 | F. M. H. M. Dupuits; P. Pop; A. Hasman; H. J. A. Schouten. "A rational request behavior: The development of prediction instruments regarding thyroid function tests in primary care". 1999. Methods of Information in Medicine. 10.1055/s-0038-1634145. | Not retrieved |
| 72 | M. Shinwa; A. Bossert; I. Chen; A. Cushing; A. S. Dunn; J. Poeran; S. Weinstein; H. J. Cho. "THINK" Before You Order: Multidisciplinary Initiative to Reduce Unnecessary Lab Testing"". 2019. Journal for healthcare quality : official publication of the National Association for Healthcare Quality. https://dx.doi.org/10.1097/JHQ.0000000000000157. | Not retrieved |
| **Identification through literature search: other methods (n = 19)** | | |
| 73 | T. A. May; M. Clancy; J. Critchfield; F. Ebeling; A. Enriquez; C. Gallagher; J. Genevro; J. Kloo; P. Lewis; R. Smith; V. L. Ng. "Reducing Unnecessary Inpatient Laboratory Testing in a Teaching Hospital". 2006. American Journal of Clinical Pathology. 10.1309/WP59YM73L6CEGX2F. | Conference Abstract |
| 74 | W. B. Applegate; M. D. Bennett; L. Chilton; B. J. Skipper; R. E. White. "Impact of a Cost‐Containment Educational Program on Housestaff Ambulatory Clinic Charges". 1983. Medical Care. | Different aim |
| 75 | N. Baral; B. C. Koner; M. Lamsal; I. Niraula; S. Dhungel. "Thyroid function testing in eastern Nepal and the impact of CME on subsequent requests". 2001. Tropical doctor. 10.1177/004947550103100314. | Different aim |
| 76 | P. M. W. Janssens; G. Wasser. "Managing laboratory test ordering through test frequency filtering". 2013. Clinical chemistry and laboratory medicine. 10.1515/cclm-2012-0841. | Different aim |
| 77 | G. Lippi; M. Brambilla; P. Bonelli; R. Aloe; A. Balestrino; A. Nardelli; G. P. Ceda; M. Fabi. "Effectiveness of a computerized alert system based on re-testing intervals for limiting the inappropriateness of laboratory test requests". 2015. Clinical biochemistry. 10.1016/j.clinbiochem.2015.06.006. | Different aim |
| 78 | J. Trietsch; B. van Steenkiste; R. Grol; B. Winkens; H. Ulenkate; J. Metsemakers; T. van der Weijden. "Effect of audit and feedback with peer review on general practitioners' prescribing and test ordering performance: a cluster-randomized controlled trial". 2017. BMC family practice. 10.1186/s12875-017-0605-5. | Different aim |
| 79 | D. W. Bates; G. J. Kuperman; E. Rittenberg; J. M. Teich; J. Fiskio; N. Ma'luf; A. Onderdonk; D. Wybenga; J. Winkelman; T. A. Brennan; A. L. Komaroff; M. Tanasijevic. "A randomized trial of a computer-based intervention to reduce utilization of redundant laboratory tests". 1999. The American journal of medicine. 10.1016/s0002-9343(98)00410-0. | No (separate) TFT reporting |
| 80 | R. L. Braham; H. S. Ruchlin. "Physician practice profiles: a case study of the use of audit and feedback in an ambulatory care group practice". 1987. Health care management review. 10.1097/00004010-198701230-00005. | No (separate) TFT reporting |
| 81 | P. S. Bunting; C. van Walraven. "Effect of a controlled feedback intervention on laboratory test ordering by community physicians". 2004. Clinical chemistry. 10.1373/clinchem.2003.025098. | No (separate) TFT reporting |
| 82 | R. Calderon-Margalit; S. Mor-Yosef; M. Mayer; B. Adler; S. C. Shapira. "An administrative intervention to improve the utilization of laboratory tests within a university hospital". 2005. International journal for quality in health care : journal of the International Society for Quality in Health Care. 10.1093/intqhc/mzi025. | No (separate) TFT reporting |
| 83 | L. C. Hampers; S. Cha; D. J. Gutglass; S. E. Krug; H. J. Binns. "The effect of price information on test-ordering behavior and patient outcomes in a pediatric emergency department". 1999. Pediatrics. | No (separate) TFT reporting |
| 84 | C. B. Lyle; R. F. Bianchi; J. H. Harris; Z. L. Wood. "Teaching cost containment to house officers at Charlotte Memorial Hospital". 1979. Journal of medical education. 10.1097/00001888-197911000-00005. | No (separate) TFT reporting |
| 85 | A. Sharma; M. Salzmann. "The effect of automated test rejection on repeat requesting". 2007. Journal of clinical pathology. 10.1136/jcp.2006.037408. | No (separate) TFT reporting |
| 86 | M. T. Silvestri; X. Xu; T. Long; T. Bongiovanni; S. L. Bernstein; S. I. Chaudhry; J. I. Silvestri; M. Stolar; E. J. Greene; J. D. Dziura; C. P. Gross; H. M. Krumholz. "Impact of Cost Display on Ordering Patterns for Hospital Laboratory and Imaging Services". 2018. Journal of general internal medicine. 10.1007/s11606-018-4495-6. | No (separate) TFT reporting |
| 87 | B. D. Sommers; N. Desai; J. Fiskio; A. Licurse; M. Thorndike; J. T. Katz; D. W. Bates. "An educational intervention to improve cost-effective care among medicine housestaff: a randomized controlled trial". 2012. Academic medicine : journal of the Association of American Medical Colleges. 10.1097/ACM.0b013e31825373b3. | No (separate) TFT reporting |
| 88 | W. H. J. M. Verstappen; T. van der Weijden; J. Sijbrandij; I. Smeele; J. Hermsen; J. M. Grimshaw; R. Grol. "Effect of a practice-based strategy on test ordering performance of primary care physicians: a randomized trial". 2003. JAMA. | No (separate) TFT reporting |
| 89 | R. A. Winkens; P. Pop; R. P. Grol; A. D. Kester; J. A. Knottnerus. "Effect of feedback on test ordering behaviour of general practitioners". 1992. BMJ (Clinical research ed.). 10.1136/bmj.304.6834.1093. | No (separate) TFT reporting |
| 90 | L. S. Feldman; H. M. Shihab; D. Thiemann; H.-C. Yeh; M. Ardolino; S. Mandell; D. J. Brotman. "Impact of providing fee data on laboratory test ordering: a controlled clinical trial". 2013. JAMA internal medicine. 10.1001/jamainternmed.2013.232. | TFTs in control group |
| 91 | V. Shalev; G. Chodick; A. D. Heymann. "Format change of a laboratory test order form affects physician behavior". 2009. International journal of medical informatics. 10.1016/j.ijmedinf.2009.04.011. | TFTs in control group |

**Abbreviations:** TFT = Thyroid Function Test.
