# Supplementary material for: Effectiveness and mechanisms of interventions to reduce low-value thyroid function tests: a systematic review
Source: Syst Rev. 2026 Feb 25;15:111. doi: 10.1186/s13643-026-03119-8 (PMC13040701; doi:10.1186/s13643-026-03119-8)
Supplement: Supplementary file 9 — Additional file 9. Additional file 9 includes additional information on study characteristics, i.e. reported outcomes, reporting on theoretical foundations, and study period. [file 13643_2026_3119_MOESM9_ESM.docx]

# **Additional Information on Study Characteristics**

| **Study and country** | **Year** | **Intervention** | **Outcomes** | | | | |  | | | | **Reporting on theoretical foundation / protocol** |
| --- | --- | --- | --- | --- | --- | --- | --- | --- | --- | --- | --- | --- |
|  |  |  | **Volume Reduction** | | **Improvement of care** | | | **Study Period** | | | |  |
|  |  |  | **Test rates** | **Expenditure** | **Appropriateness** | **Pattern** | **CoV** | **Pre intervention period** | **Duration (months)** | **Post intervention period** | **Duration (months)** |  |
| **Studies identified in present review (n=21)** | | | | | | | | | | | | |
| Bateman et al, Canada (1) | 2019 | Education + Feedback | + |  |  |  |  | September - December 2017 | 4 | January - June 2018 | 6 | Reporting on theoretical foundation* |
| Bejjanki et al, USA (2) | 2018 | Decision tool | + | + |  |  |  | May 2014 - September 2015 | 17 | October 2015 - February 2017 | 17 |  |
| Bellodi et al, Italy (3) | 2017 | Decision tool | + |  |  |  |  | September - November 2014 | 3 | September - November 2015 | 3 |  |
| Bradshaw et al, USA (4) | 2021 | Decision tool | + |  |  |  |  | January - March 2018 | 3 | January - March 2019 | 3 |  |
| Caldarelli et al, Italy (5) | 2017 | Decision tool | + | + | + |  |  | 2012 | 12 | 2013 - 2014 | 24 |  |
| Chami et al, Canada (6) | 2021 | Decision tool | + |  |  |  |  | April 2006 - October 2012 | 79 | November 2012 - March 2018 | 65 |  |
| Dalal et al, USA (7) | 2017 | Decision tool | + |  |  |  |  | May - August 2014 | 3 | May - August 2015 | 3 |  |
| Delvaux et al, Belgium (8) | 2020 | Decision tool |  |  | + |  |  | NA | - | December 2017 - June 2018 | 7 | Trial protocol available (9)  Reporting on theoretical foundation** |
| Elrewini et al, Saudi Arabia (10) | 2022 | Education + Guidelines |  | + | + |  |  | January - June 2019 | 6 | July 2019 - June 2020 | 12 | Reporting on theoretical foundation* |
| Gilmour et al, Canada (11) | 2017 | Education + Decision tool | + |  |  |  |  | October 2013 - September 2014 | 12 | December 2014 - August 2015 | 9 | Reporting on theoretical foundation* |
| Janssens et al, Netherlands (12) | 2015 | Guidelines | + | + |  |  |  | 2012 | NR | 2012 | NR |  |
| Krouss et al, USA (13) | 2022 | Decision tool | + |  |  |  |  | May 18, 2020 to May 23, 2021 | 12 | May 23, 2021 to February 27, 2022 | 9 |  |
| Leis et al, Canada (14) | 2019 | Decision tool | + |  | + |  |  | February - June 1997 | 5 | February - June 2004 | 5 | Reporting on theoretical foundation* |
| Leung et al, USA (15) | 2017 | Education + Reminder |  | + |  |  |  | May - June 2016 | 2 | May - June 2017 | 2 |  |
| MacPherson et al, Australia (16) | 2005 | Guidelines + Decision tool | + |  |  |  |  | November 2013 - January 2014 | 3 | November 2014 - January 2015 | 3 |  |
| Muris et al, Netherlands (17) | 2021 | Decision tool | + |  |  |  |  | September 2017 - March 2019 | 30 | September 2019 - March 2020 | 7 |  |
| Notas et al, Greece (18) | 2018 | Decision tool | + |  |  |  |  | April 2009 - March 2013 | 48 | April 2013 - March 2016 | 36 |  |
| Salinas et al, Spain (19) | 2016 | Decision tool | + |  |  |  |  | January 2010 - April 2011 | 16 | May 2011 - December 2014 | 32 |  |
| Sue et al, USA (20) | 2019 | Decision tool | + |  |  |  |  | July 2018 - September 2018 | 1.5 | September 2018-January 2019 | 3.5 |  |
| Taher et al, Canada (21) | 2020 | Decision tool | + |  |  |  |  | October 2015 - March 2016 | 6 | April 2016 - February 2019 | 34 | Reporting on theoretical foundation* |
| Wintemute et al, Canada (22) | 2019 | Guidelines + Feedback | + |  |  |  |  | January - December 2016 | 12 | January - December 2017 | 12 | Reporting on theoretical foundation* |
| **Studies included in previous review (n=27)** | | | | | | | | | | | | |
| Adlan et al, UK (23) | 2011 | Guidelines | + | + |  |  |  | July - December 2004 | 6 | July - December 2008 | 6 |  |
| Baker et al, UK (24) | 2003 | Guidelines + Feedback | + |  |  |  |  | April 1999 - June 1999 | 3 | July 1999 - March 2000 | 9 |  |
| Berwick and Coltin, USA (25) | 1986 | Feedback/ Education | + |  |  |  | + | NR | 12 | NR | 12 |  |
| Chu et al, Australia (26) | 2013 | Decision tool | + |  |  |  |  | January - June 2009 | 5 | January - June 2010 | 5 |  |
| Cipullo and Mostoufizadeh, USA (27) | 1996 | Guidelines | + |  |  |  |  | 1993 - 1994 | NR | 1994 - 1995 | NR |  |
| Daucourt et al, France (28) | 2000 | Reminder / Decision Tool / Reminder + Decision tool |  |  | + |  |  | NA | - | January - May 2000 | 5 |  |
| Dowling et al, USA (29) | 1989 | Education + Feedback | + |  | + |  |  | NR | NR | NR | 7 |  |
| Emerson and Emerson, USA (30) | 2001 | Decision tool | + |  |  | + |  | January - June | 6 | July - July | 13 |  |
| Feldkamp and Carey, USA (31) | 1996 | Decision tool | + |  | + | + |  | 1989 | NR | 1993 | 36 |  |
| Gama et al, UK (32) | 1991 | Feedback | + |  |  |  |  | November - April 1988 | 6 | May - October 1989 | 6 |  |
| Grivell et al, Australia (33) | 1981 | Feedback | + |  |  |  |  | 1979 | 12 | 1980 | 12 |  |
| Hardwick et al, Canada (34) | 1982 | Guidelines + Change in Funding | + | + |  | + |  | 1974-September 1976 | 31 | October 1976-1979 | 39 |  |
| Horn et al, USA (35) | 2013 | Decision tool | + |  |  |  |  | April2010-April2011 | 12 | May-November 2011 | 6 |  |
| Larsson et al, Sweden (36) | 1999 | Education |  |  |  | + |  | February and March 1996 | 2 | February and March 1997 | 2 |  |
| Mindemark and Larsson, Sweden (follow up) (37) | 2009 | Education |  |  |  | + |  | April - June 2002 | 3 | April - September 2002 | 6 |  |
| Nightingale et al, UK (38) | 1994 | Guidelines + Decision tool + Feedback |  |  | + |  |  | 1990 | 12 | 1991 | 12 |  |
| Rhyne and Gehlbach, USA (39) | 1979 | Education + Guidelines | + |  | + |  |  | October 1976 - March 1977 | 6 | June - November 1977 | 6 |  |
| Schectman et al, USA (40) | 1991 | Education + Reminder + (Feedback) | + |  | + |  |  | NR | NR | NR | 12 |  |
| Stuart et al, Australia (41) | 2002 | Education + Guidelines + Feedback |  | + |  |  |  | November 1998 - April 1999 | 6 | November 1999 - October 2000 | 24 | Reporting on theoretical foundation* |
| Thomas et al, UK (42) | 2006 | Reminder/ Feedback / Reminder + Feedback | + |  |  |  |  | February 2001 - January 2002 | 12 | February 2002 - January 2003 | 12 | Trial protocol available (43)  Reporting on theoretical foundation** |
| Tierney et al, USA (44) | 1988 | Decision tool |  | + |  |  |  | NA | 0 | March - September 1986 | 6 |  |
| Tomlin et al, New Zealand (45) | 2011 | Education + Guidelines + Feedback | + |  |  | + |  | October 2003 - September 2005 | 24 | October 2005 - December 2007 | 27 | Reporting on theoretical foundation** |
| Toubert et al, France (46) | 2000 | Guidelines + Reminders | + |  | + | + |  | June - July 1996 | 2 | June -July 1997 and 1998 | 4 |  |
| Van Walraven et al, Canada (47) | 1998 | Guidelines + Change in Funding + Decision tool | + |  |  | + |  | July 1991 - October 1993 | 28 | November 1993 - April 1997 | 42 |  |
| Vidal-Trecan et al, France (48) | 2003 | Education + Guidelines + Reminders + Decision tool | + |  |  | + |  | June 1995 | 1 | July 1995 - June 1998 | 36 |  |
| Willis and Datta, UK (49) | 2013 | Education + Guidelines | + |  |  |  |  | September 2008 - August 2009 | 12 | September 2009 - Aug 2010 | 12 |  |
| Wong et al, USA (50) | 1983 | Guidelines + Decision tool | + |  |  | + |  | November 1980 - October 1981 | 11.5 | October 1981 - May 1982 | 6.5 |  |

* Reporting on theoretical foundation discussed in our Systematic Review. For relevant text passages see table below.

** Reporting on theoretical foundation not discussed explicitly in our Systematic Review. See table below for theory referenced.

**Abbreviations:** CoV = Coefficient of Variation, NA = Not Applicable, NR = Not Reported.

# Reporting on theoretical foundation:

| **Study and country** | **Reporting on theoretical foundation** |
| --- | --- |
| Bateman et al, Canada (1) | *“A fish bone framework was used for root cause analysis and to identify possible avenues, facilitators, and barriers to intervention. We identified five domains (patient, physician, team, institution and system) with a total of 16 contributing causes to admission Vitamin D and TSH orders. Patient factors include clinically appropriate reasons for testing Vitamin D and/or TSH. The remaining domains encompass causes of admission Vitamin D and TSH orders that are outside the scope of clinical practice guidelines relevant to the study population, and likely lead to unnecessary or inappropriate testing.”*  *“Using the key factors identified from root cause analysis, a series of interventions were planned. For PDSA Cycle #1, we devised an academic detailing education intervention to target two physician factors, possible knowledge gap for best practices and lack of appreciation of potential harms of unnecessary testing, and one institutional factor, lack of awareness of the scope and cost of the problem. Simultaneously, CCDS was used to restrict Vitamin D orders to target two physician factors, habit and possible knowledge gap for appropriate indications for measuring Vitamin D […]. For PDSA Cycle #2, we devised an audit and feedback intervention to target two physician factors, automatic ordering in ‘favourite’ admission caresets and habitual ordering of Vitamin D and/or TSH, as well as two system factors, CPOE-enabled favourite caresets and CPOE-related ease of ordering inappropriate tests. […] A third PDSA cycle was devised to solicit feedback and disseminate results.”* |
| Delvaux et al, Belgium (8) | Search for additional literature by co-authors revealed an article on factors associated with CDSS success and a checklist for successfully implementing CDSS. This literature was not cited in the article:  Van de Velde S, Kunnamo I, Roshanov P, Kortteisto T, Aertgeerts B, Vandvik PO, Flottorp S; GUIDES expert panel. The GUIDES checklist: development of a tool to improve the successful use of guideline-based computerised clinical decision support. *Implement Sci*. 2018 Jun 25;13(1):86. doi: 10.1186/s13012-018-0772-3.  Van de Velde S, Heselmans A, Delvaux N, Brandt L, Marco-Ruiz L, Spitaels D, Cloetens H, Kortteisto T, Roshanov P, Kunnamo I, Aertgeerts B, Vandvik PO, Flottorp S. A systematic review of trials evaluating success factors of interventions with computerised clinical decision support. *Implement Sci*. 2018 Aug 20;13(1):114. doi: 10.1186/s13012-018-0790-1. |
| Elrewini et al, Saudi Arabia (10) | *“The whole process of TSH test requisition and processing was analyzed to identify the root causes and solutions as shown in the Ishikawa diagram. […] The interventions were implemented based on the formulated action plan within the planned framework.”* |
| Gilmour et al, Canada (11) | *“The model for improvement framework for continuous QI was used for project development. Iterative plan‐do‐study‐act cycles were used to refine 2 change ideas. The first change idea focused on providing education to physicians regarding appropriate indications for fT4/fT3 testing; this was accomplished via lectures, emails, and postings in clinical workspaces. The second change idea was to implement a forced‐*  *function reflex fT4 system, in which fT4 was only processed if the TSH was outside the laboratory's reference range or if a clinical justification was provided on the laboratory requisition.”* |
| Leis et al, Canada (14) | *“Most of the orders were thought to represent verbal orders from the physician or transcription errors. From this it was hypothesised that frequent ordering was driven by the format of the admission order set, namely the inclusion of a checkbox for TSH ordering. We proceeded to test this theory in a simulation, the details of which have been published elsewhere, […] and found that the presence of the checkbox did appear to influence ordering.”* |
| Stuart et al, Australia (41) | *“The PRECEDE (Predisposing, Reinforcing, Enabling, Causes in Educational Diagnosis and Evaluation) model was selected because it provides a practical framework for understanding behaviour modification in the context of test ordering by clinicians.[…] The model classifies factors that assist or inhibit behaviour change in to three groups: predisposing, enabling and reinforcing. Predisposing factors consist of preexisting attitudes or knowledge that support test-ordering practice. Enabling factors comprise individual skills, available resources and structural barriers that assist or inhibit appropriate test-ordering behaviour. Reinforcing factors relate to feedback that can positively or negatively influence practice. The intervention reflected the core elements of the PRECEDE model and comprised three integrated components:*  *■ an education program for medical staff (addressing predisposing factors);*  *■ implementation of a protocol for test ordering (addressing enabling factors);*  *■ an audit/feedback process (addressing reinforcing factors).”* |
| Taher et al, Canada (21) | *“To design a QI intervention, the Model for Improvement Framework was used […]. Based on the results from the baseline analysis and other*  *published literature […], the project aim was to reduce fT4 and fT3 testing by 30% from baseline within 1 year. Two change ideas were*  *implemented in April 2016 based off recommendations made in the free thyroid hormone Choosing Wisely toolkit […].”* |
| Thomas et al, UK (42) | Citation of PRECEDE framework by Solomon et al. as referenced by Stuart et al. (41) (see above). Model not explicitly applied. Additional reference to Systematic Review by Grimshaw et al. in order to back the following passage:  *“Furthermore, current systematic reviews suggest that single-intervention strategies could be as effective as multiple complex interventions in changing health-profession practice.”*  Grimshaw JM, Thomas RE, MacLennan G, et al. Effectiveness and efficiency of guideline dissemination and implementation strategies. *Health Technol Assess* 2004; 8: 1–72.  Solomon DH, Hashimoto H, Daltroy L, Liang MH. Techniques to improve physicians’ use of diagnostic tests: a new conceptual framework. *JAMA* 1998; 280: 2020–27. |
| Tomlin et al, New Zealand (45) | Reference of various articles/studies to justify multifaceted intervention. To be found in the following passage  *“There is evidence that multifaceted interventional programmes are needed before guidelines recommendations have a chance of being implemented.”* |
| Wintemute et al, Canada (22) | *“According to Rogers’ theory of diffusion of innovations, uptake is greater if a change is actively promoted by local opinion leaders; is compatible with values of adopters; is not overly complex; and if local adaptation is allowed.[...] The ARTIC project deliberately focused on local physician leadership and allowed local customization after agreement on general aims. There was financial and operational support at each site for local leaders, as well as ongoing communication and shared learning between champions at different sites, consistent with recom-mendations and principles of a Learning Healthcare System. […]”* |

**Abbreviations:** CDSS = Clinical Decision Support System, CPOE = Computerised Order Entry, fT3 = free Triiodothyronine, fT4 = free Thyroxine, PDSA = Plan-Do-Study-Act, QI = Quality Improvement, TSH = Thyroid-Stimulating Hormone.

Literature Cited

1. Bateman EA, Gob A, Chin-Yee I, MacKenzie HM. Reducing waste: A guidelines-based approach to reducing inappropriate Vitamin D and TSH testing in the inpatient rehabilitation setting. BMJ Open Qual. 2019; 8(4).

2. Bejjanki H, Mramba LK, Beal SG, Radhakrishnan N, Bishnoi R, Shah C et al. The role of a best practice alert in the electronic medical record in reducing repetitive lab tests. ClinicoEconomics and outcomes research : CEOR 2018; 10:611–8.

3. Bellodi E, Vagnoni E, Bonvento B, Lamma E. Economic and organizational impact of a clinical decision support system on laboratory test ordering. BMC medical informatics and decision making 2017; 17(1):179.

4. Bradshaw AB, Bonnecaze AK, Burns CA, Beardsley JR. Impact of an Interprofessional Collaborative Quality Improvement Initiative to Decrease Inappropriate Thyroid Function Testing. Hosp. Pharm. 2021; 56(5):481–5.

5. Caldarelli G, Troiano G, Rosadini D, Nante N. Adoption of TSH Reflex algorithm in an Italian clinical laboratory. Annali di igiene : medicina preventiva e di comunita 2017; 29(4):317–22.

6. Chami N, Li Y, Weir S, Wright JG, Kantarevic J. Effect of Strict and Soft Policy Interventions on Laboratory Diagnostic Testing in Ontario, Canada: A Bayesian Structural Time Series Analysis. Health policy 2021; 125(2):254–60.

7. Dalal S, Bhesania S, Silber S, Mehta P. Use of electronic clinical decision support and hard stops to decrease unnecessary thyroid function testing. BMJ Open Qual. 2017; 6(1):u223041. w8346.

8. Delvaux N, Piessens V, Burghgraeve T de, Mamouris P, Vaes B, Stichele RV et al. Clinical decision support improves the appropriateness of laboratory test ordering in primary care without increasing diagnostic error: the ELMO cluster randomized trial. Implementation science : IS 2020; 15(1):100.

9. Evidence-based Laboratory Test Order Sets in Primary Care; 2016. Available from: URL: https://clinicaltrials.gov/study/NCT02950142.

10. Elrewini AM, Zubair M, Afridi NK, Dildar MT, Javed H, Alwalah SM. To determine the effectiveness of different interventions to reduce unnecessary requests of serum thyroid stimulating hormone levels in a hospital. The Professional Medical Journal 2022; 29(05):686–92.

11. Gilmour JA, Weisman A, Orlov S, Goldberg RJ, Goldberg A, Baranek H et al. Promoting resource stewardship: Reducing inappropriate free thyroid hormone testing. J. Eval. Clin. Pract. 2017; 23(3):670–5.

12. Janssens PMW, Staring W, Winkelman K, Krist G. Active intervention in hospital test request panels pays. Clinical chemistry and laboratory medicine 2015; 53(5):731–42.

13. Krouss M, Israilov S, Alaiev D, Hupart K, Da Shin W, Mestari N et al. Free the T3: implementation of best practice advisory to reduce unnecessary orders. The American journal of medicine 2022; 135(12):1437–42.

14. Leis B, Frost A, Bryce R, Lyon AW, Coverett K. Altering standard admission order sets to promote clinical laboratory stewardship: A cohort quality improvement study. BMJ Qual. Saf. 2019; 28(10):846–52.

15. Leung E, Song S, Al-Abboud O, Shams S, English J, Naji W et al. An educational intervention to increase awareness reduces unnecessary laboratory testing in an internal medicine resident-run clinic. Journal of community hospital internal medicine perspectives 2017; 7(3):168–72.

16. MacPherson RD, Reeve SA, Stewart TV, Cunningham AES, Craven ML, Fox G et al. Effective strategy to guide pathology test ordering in surgical patients. ANZ journal of surgery 2005; 75(3):138–43.

17. Muris DMJ, Molenaers M, Nguyen T, Bergmans, P. W. M. P., van Acker BAC, Krekels MME et al. Effect of a price display intervention on laboratory test ordering behavior of general practitioners. BMC Fam. Pract. 2021; 22(1).

18. Notas G, Kampa M, Malliaraki N, Petrodaskalaki M, Papavasileiou S, Castanas E. Implementation of thyroid function tests algorithms by clinical laboratories: A four-year experience of good clinical and diagnostic practice in a tertiary hospital in Greece. Eur. J. Intern. Med. 2018; 54:81–6.

19. Salinas M, López-Garrigós M, Flores E, Leiva-Salinas M, Asencio A, Lugo J et al. Managing inappropriate requests of laboratory tests: From detection to monitoring. Am. J. Managed Care 2016; 22(9):e311-e316.

20. Sue LY, Kim JE, Oza H, Chong T, Woo HE, Cheng EM et al. Reducing Inappropriate Serum T3 Laboratory Test Ordering in Patients with Treated Hypothyroidism. Endocr. Pract. 2019; 25(12):1312–6.

21. Taher J, Beriault DR, Yip D, Tahir S, Hicks LK, Gilmour JA. Reducing free thyroid hormone testing through multiple Plan-Do-Study-Act cycles. Clin. Biochem. 2020; 81:41–6.

22. Wintemute K, Greiver M, McIsaac W, Del Elisabeth Giudice M, Sullivan F, Aliarzadeh B et al. Choosing Wisely Canada campaign associated with less overuse of thyroid testing Retrospective parallel cohort study. Can. Fam. Phys. 2019; 65(11):E487-E496.

23. Adlan MA, Neel V, Lakra SS, Bondugulapati LNR, Premawardhana, L. D. K. E. Targeted thyroid testing in acute illness: Achieving success through audit. J. Endocrinol. Invest. 2011; 34(8 SUPPL.):e210-e213.

24. Baker R, Smith JF, Lambert PC. Randomised controlled trial of the effectiveness of feedback in improving test ordering in general practice. Scand. J. Prim. Health Care 2003; 21(4):219–23.

25. Berwick DM, Coltin KL. Feedback reduces test use in a health maintenance organization. J. Am. Med. Assoc. 1986; 255(11):1450–4.

26. Chu KH, Wagholikar AS, Greenslade JH, O'Dwyer JA, Brown AF. Sustained reductions in emergency department laboratory test orders: Impact of a simple intervention. Postgrad. Med. J. 2013; 89(1056):566–71.

27. Cipullo JA, Mostoufizadeh M. Bringing order to test orders: one lab's story. CAP today 1996; 10(1):20–2.

28. Daucourt V, Saillour-Glénisson F, Michel P, Jutand MA, Abouelfath A. A multicenter cluster randomized controlled trial of strategies to improve thyroid function testing. Med. Care 2003; 41(3):432–41.

29. Dowling PT, Alfonsi G, Brown MI, Culpepper L. An education program to reduce unnecessary laboratory tests by residents. J. Med. Educ. 1989; 64(7):410–2.

30. Emerson JF, Emerson SS. The impact of requisition design on laboratory utilization. AM. J. CLIN. PATHOL. 2001; 116(6):879–84.

31. Feldkamp CS, Carey JL. An algorithmic approach to thyroid function testing in a managed care setting: 3-Year experience. AM. J. CLIN. PATHOL. 1996; 105(1):11–6.

32. Gama R, Nightingale PG, Broughton PM, Peters M, Bradby GV, Berg J et al. Feedback of laboratory usage and cost data to clinicians: does it alter requesting behaviour? Annals of clinical biochemistry 1991; 28 (Pt 2):143–9.

33. Grivell AR, Forgie HJ, Fraser CG, Berry MN. Effect of feedback to clinical staff of information on clinical biochemistry requesting patterns. Clinical chemistry 1981; 27(10):1717–20.

34.  Hardwick DF, Morrison JI, Tydeman J, Cassidy PA, Chase WH. Structuring complexity of testing: a process oriented approach to limiting unnecessary laboratory use. The American journal of medical technology 1982; 48 7:605–8.

35. Horn DM, Koplan KE, Senese MD, Orav EJ, Sequist TD. The impact of cost displays on primary care physician laboratory test ordering. Journal of general internal medicine 2014; 29(5):708–14.

36. Larsson A, Biom S, Wernroth ML, Hultén G, Tryding N. Effects of an education programme to change clinical laboratory testing habits in primary care. Scandinavian journal of primary health care 1999; 17(4):238–43.

37. Mindemark M, Larsson A. Long-term effects of an education programme on the optimal use of clinical chemistry testing in primary health care. Scandinavian journal of clinical and laboratory investigation 2009; 69(4):481–6.

38. Nightingale PG, Peters M, Mutimer D, Neuberger JM. Effects of a computerised protocol management system on ordering of clinical tests. Quality in health care : QHC 1994; 3(1):23–8.

39. Rhyne RL, Gehlbach SH. Effects of an educational feedback strategy on physician utilization of thyroid function panels. The Journal of family practice 1979; 8(5):1003–7.

40. Schectman JM, Elinsky EG, Pawlson LG. Effect of Education and Feedback on Thyroid Function Testing Strategies of Primary Care Clinicians. Arch. Intern. Med. 1991; 151(11):2163–6.

41. Stuart PJ, Crooks S, Porton M. An interventional program for diagnostic testing in the emergency department. The Medical journal of Australia 2002; 177(3):131–4.

42. Thomas RE, Croal BL, Ramsay C, Eccles M, Grimshaw J. Effect of enhanced feedback and brief educational reminder messages on laboratory test requesting in primary care: a cluster randomised trial. Lancet (London, England) 2006; 367(9527):1990–6.

43. Croal B. Diagnostic Request Advisory Model; 2006. Available from: URL: https://www.isrctn.com/ISRCTN06490422?q=ISRCTN06490422&filters=&sort=&offset=1&totalResults=1&page=1&pageSize=10.

44. Tierney WM, McDonald CJ, Hui SL, Martin DK. Computer predictions of abnormal test results. Effects on outpatient testing. J. Am. Med. Assoc. 1988; 259(8):1194–8.

45. Tomlin A, Dovey S, Gauld R, Tilyard M. Better use of primary care laboratory services following interventions to 'market' clinical guidelines in New Zealand: A controlled before-and-after study. BMJ Qual. Saf. 2011; 20(3):282–90.

46. Toubert ME, Chevret S, Cassinat B, Schlageter MH, Beressi JP, Rain JD. From guidelines to hospital practice: Reducing inappropriate ordering of thyroid hormone and antibody tests. Eur. J. Endocrinol. 2000; 142(6):605–10.

47. van Walraven C, Goel V, Chan B. Effect of population-based interventions on laboratory utilization: A time-series analysis. J. Am. Med. Assoc. 1998; 280(23):2028–33.

48. Vidal-Trécan G, Toubert ME, Coste J, Paycha F, Durand-Zaleski I, Fulla Y et al. Reducing the number of T3 orders in the Paris hospital network: Towards better appropriatness of thyroid function test prescription. Ann. Endocrinol. 2003; 64(3):210–5.

49. Willis EA, Datta BN. Effect of an educational intervention on requesting behaviour by a medical admission unit. Ann. Clin. Biochem. 2013; 50(2):166–8.

50. Wong ET, McCarron MM, Shaw ST. Ordering of Laboratory Tests in a Teaching Hospital: Can It Be Improved? JAMA 1983; 249(22):3076–80.
