# Supplementary material for: Effectiveness and mechanisms of interventions to reduce low-value thyroid function tests: a systematic review
Source: Syst Rev. 2026 Feb 25;15:111. doi: 10.1186/s13643-026-03119-8 (PMC13040701; doi:10.1186/s13643-026-03119-8)
Supplement: Supplementary file 10 — Additional file 10. Additional file 10 includes the visualisation of the Risk of Bias assessment for the (cluster) RCTs and controlled studies. [file 13643_2026_3119_MOESM10_ESM.docx]

# Risk of Bias Assessment

We found the Effective Public Health Practice Project tool (EPHPP) used by Zhelev et al. (1) to be generally useful for assessing study design (2), but it did not fully address the complexities of the various study designs in our context. Therefore, we applied more specific tools to assess the risk of bias: the Cochrane Risk of Bias 2.0 tool (RoB 2) for (cluster) randomised controlled studies (RCTs) and the ROBINS-I tool (Risk of Bias In Non-randomised Studies - of Interventions) for non-randomised studies (3, 4). Risk of bias figures were generated for each outcome domain separately using the robvis application (5). Two reviewers (CP, MH) independently conducted the assessments, and any discrepancies were resolved through discussion with a third reviewer (GG). We assessed and verified data extraction and risk of bias for the studies in our review apart from the results presented by Zhelev et al. (1).

## RoB 2: Cluster RCTs (n = 4)


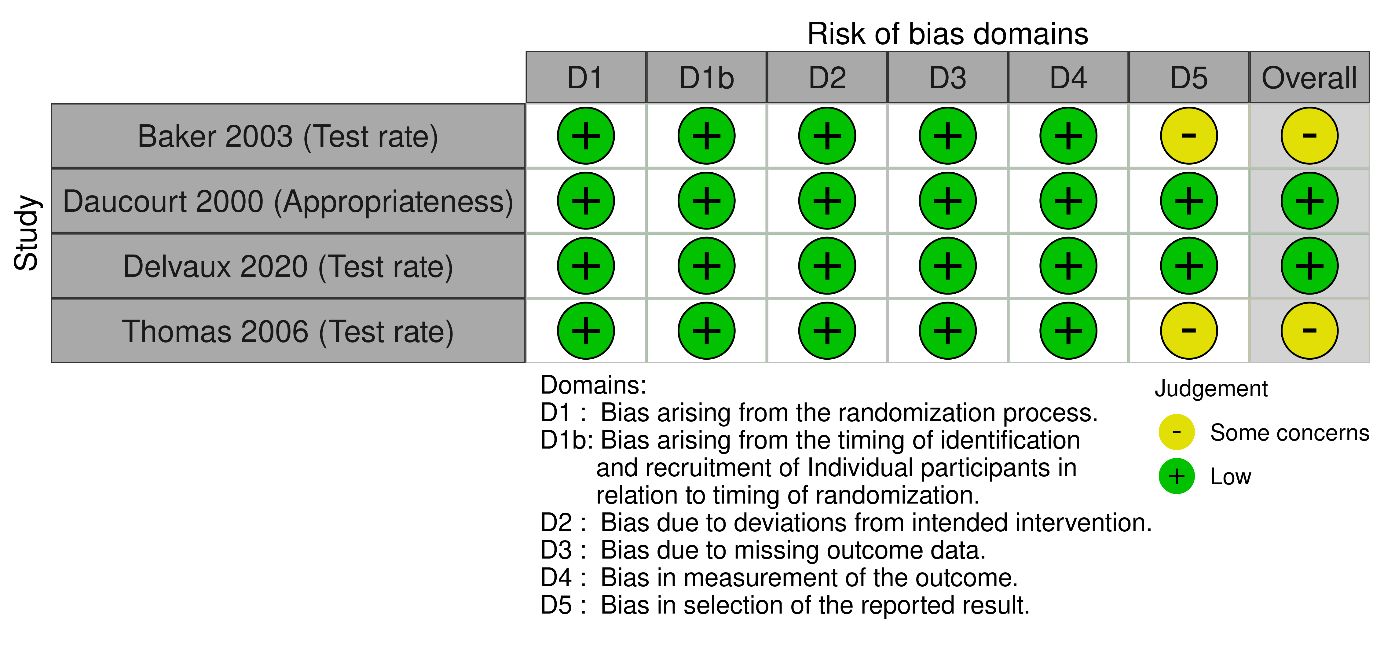


## RoB 2: RCTs (n = 1)


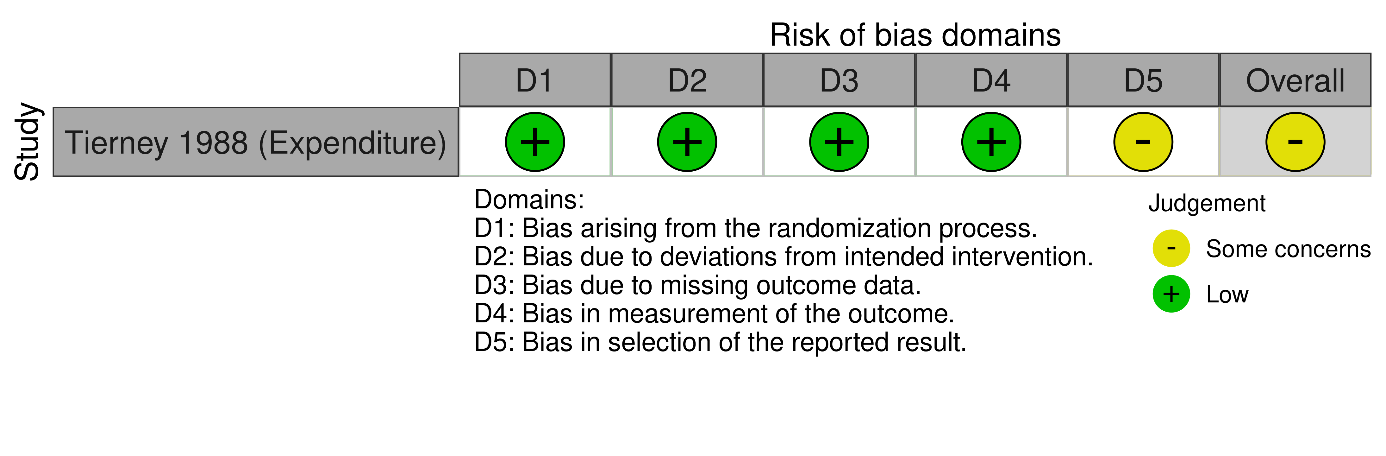


## ROBINS-I: Controlled Studies (n = 9)


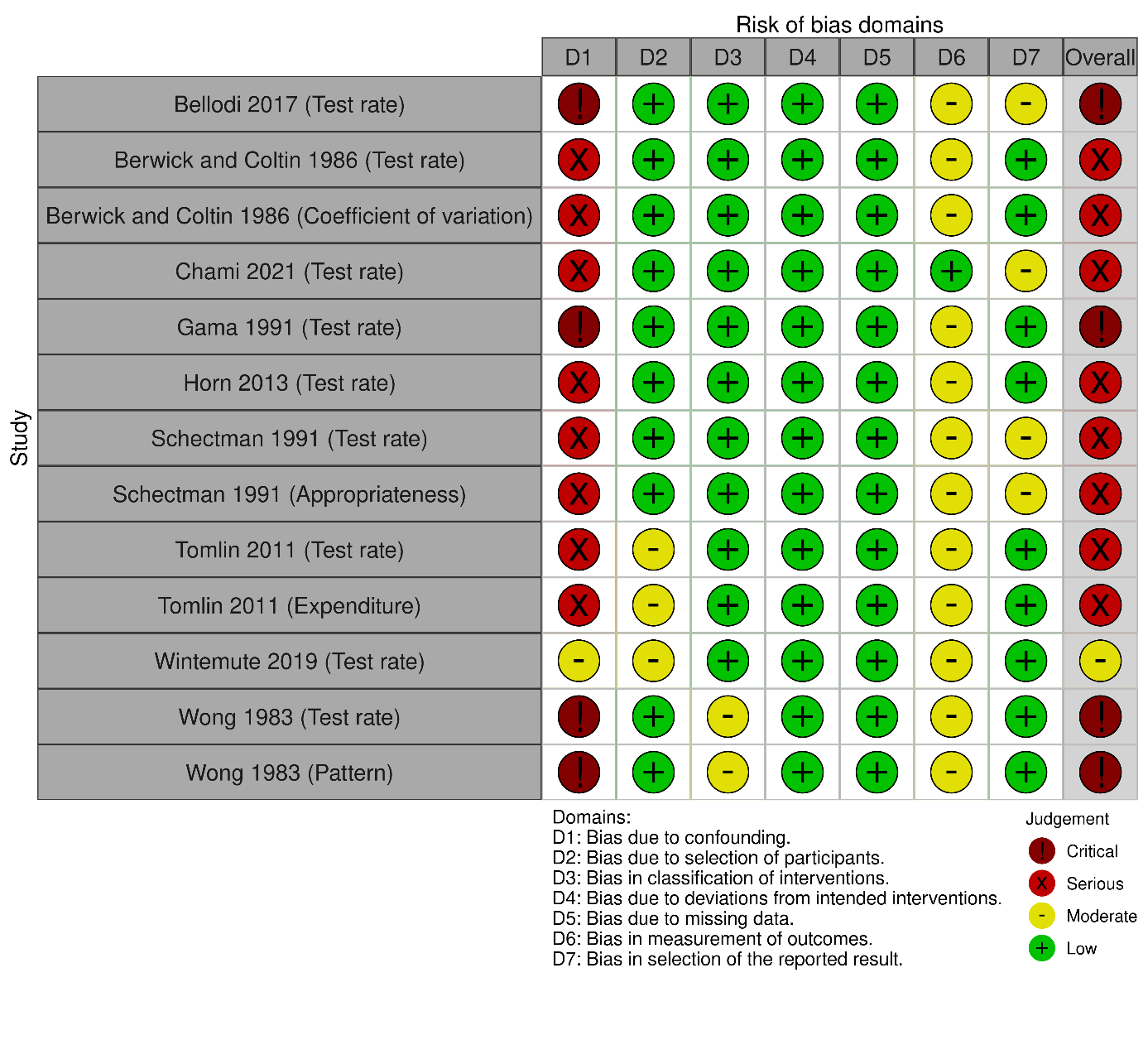


Literature Cited

1. Zhelev Z, Abbott R, Rogers M, Fleming S, Patterson A, Hamilton WT et al. Effectiveness of interventions to reduce ordering of thyroid function tests: a systematic review. BMJ open 2016; 6(6):e010065.

2. McMaster Evidence Review & Synthesis Team. Effective Public Health Practice Project (EPHPP): Quality Assessment Tool for Quantitative Studies; 2022. Available from: URL: https://merst.healthsci.mcmaster.ca/ephpp/.

3. Sterne JA, Hernán MA, Reeves BC, Savović J, Berkman ND, Viswanathan M et al. ROBINS-I: a tool for assessing risk of bias in non-randomised studies of interventions. BMJ 2016; 355:i4919.

4. Sterne JAC, Savović J, Page MJ, Elbers RG, Blencowe NS, Boutron I et al. RoB 2: a revised tool for assessing risk of bias in randomised trials. BMJ 2019; 366:l4898.

5. McGuinness LA, Higgins JPT. Risk-of-bias VISualization (robvis): An R package and Shiny web app for visualizing risk-of-bias assessments. Res Synth Methods 2021; 12(1):55–61.
