# Supplementary material for: Effectiveness and mechanisms of interventions to reduce low-value thyroid function tests: a systematic review
Source: Syst Rev. 2026 Feb 25;15:111. doi: 10.1186/s13643-026-03119-8 (PMC13040701; doi:10.1186/s13643-026-03119-8)
Supplement: Supplementary file 11 — Additional file 11. Additional file 11 includes additional information on study results, i.e. the outcome values (pre/postintervention), notes on outcome measures and statistical indicators (confidence interval, p-value, relative reduction, difference in means). [file 13643_2026_3119_MOESM11_ESM.docx]

# **Additional Information on Study Results**

| **Study and country** | **Type of Study** | **Intervention** | **Outcome Preintervention** | **Outcome Postintervention** | **Difference in means** | **CI** | **p-value** | **Relative Reduction^a^** | **Notes on outcome measure** |
| --- | --- | --- | --- | --- | --- | --- | --- | --- | --- |
| **Appropriateness (Improvement of care)** | | | | | | | | | |
| Daucourt et al, France MPC (1) | RCT | Reminder | 73.9% | I (C): 73.4 (62.0%) | NR | PI: [56.7, 90.1] | 0.37 | +18.4%^b^ | Proportion of TFTs ordered in accordance with guidelines. |
| Daucourt et al, France TRF (1) | RCT | Decision tool | 73.9% | I (C): 82.6 (62.0%) | NR | PI: [73.1, 92.1] | < 0.01^d^ | +30.2%^b^ | See above. |
| Daucourt et al, France Both (1) | RCT | Reminder + Decision tool | 73.9% | I (C): 77.9 (62.0%) | NR | PI: [68.9, 87.0] | NR | +25.7%^b^ | See above. |
| Delvaux et al, Belgium (2) | RCT | Decision tool | NA | I: 0.5 C: 0.45 | 0.05 (0.01–0.09) | PI I: [0.47, 0.54] PI C: [0.42, 0.49] | 0.0136^d^ | +11.1%^b^ | Appropriate tests / total orders. |
| Schectman et al, USA Reminder + Feedback (3) | C | Reminder + Feedback | 42% | 64% | NR | NR | NR | 52.4%^b^ | Compliance rate with TFT protocol. |
| Schectman et al, USA Reminder (3) | C | Reminder | 29% | 79% | NR | NR | 0.005^d^ | 172%^b^ | See above. |
| Caldarelli et al, Italy (4) | NC | Decision tool | TSH/FT4: 1.19 TSH/FT3: 1.23 TSH reflex/TSH: - | TSH/FT4: 1.28 TSH/FT3: 1.4 TSH reflex/TSH: 0.1 | NR | NR | NR | TSH/FT4: +7.6% TSH/FT3: +13.8%^b^ TSH reflex/TSH: NA | Prescriptive appropriateness through the ratios TSH / FT4, TSH / FT3 and "TSH Reflex" / TSH. |
| Dowling et al, USA (5) | NC | Education + Feedback | 0.0023 | 0.0030 | NR | NR | > 0.05 | +30.4%^b^ | Indicated TSH / visit. |
| Elrewini et al, Saudi Arabia (6) | NC | Education + Guidelines | 60% | 49% | NR | NR | < 0.05^d^ | -18.3%^b^ | Unnecessary requests / total TSH requests. |
| Feldkamp and Carey, USA (7) | NC | Decision tool | Share of TSH: 48.0% | Share of TSH: 72.3% | NR | NR | NR | +50.6%^b^ | Shift towards TSH. |
| Leis et al, Canada (8) | NC | Decision tool | Order not indicated: 60.6% Test w/o indication: 73.3% Indication w/o test: 36.8% Overall appropriateness: 46.2% Overall test rate: 85.4% | Order not indicated: 20% Test w/o indication: 2.7%  Indication w/o test: 81.8% Overall appropriateness: 61.9%^b^ Overall test rate: 36.4% | NR | NR | 0.01^d^ < 0.0001^d^ < 0.0001^d^ NR < 0.0001^d^ | -67.0%^b^ -96.3%^b^ +122%^b^ + 34.0%^b^ -57.4%^b^ | Proportion of not indicated physician TSH orders. |
| Nightingale et al, UK (9) | NC | Guidelines + Decision tool + Feedback | 57% ^c^ | 81% ^c^ | NR | NR | NR | +42%^b^ | Patients requiring an investigation / patients tested. |
| Rhyne and Gehlbach, USA (10) | NC | Education + Guidelines | High indication: 45% Low indication: 29% | High indication: 23% Low indication: 19% | NR | NR | > 0.05 | 48.9%^b^ 34.4%^b^ | High / low indication test proportion. |
| Toubert et al, France (11) | NC | Guidelines + Reminders | 45% | 67% | NR | NR | < 0.0001^d^ | +48.9%^b^ | Frequency of appropriate use of thyroid function tests. |
| **Coefficient of variation (Improvement of care)** | | | | | | | | | |
| Berwick and Coltin, USA PCF$ (12) | C | Feedback | NR | I (C): -23.6% (+15.7%) | NR | NR | NR | - 34.0%^b^ | CoV of rate of test use among physicians within centres. |
| Berwick and Coltin, USA PCFY (12) | C | Feedback | NR | I (C): -28.2% (+15.7%) | NR | NR | NR | -37.9%^b^ | See above. |
| Berwick and Coltin, USA TSE (12) | C | Education | NR | I (C): +0.5% (+15.7%) | NR | NR | NR | - 13.1%^b^ | See above. |
| **Expenditure (Volume Reduction)** | | | | | | | | | |
| Tierney et al, USA (13) | RCT | Decision tool | NR | I: 1.12$ C: 1.25$ | NR | NR | > 0.05 | -10.3% | Charges per visit. |
| Tomlin et al, New Zealand (14) | C | Education + Guidelines + Feedback | TSH:FT4 2.4:1 TSH:FT3 7.7:1 | TSH:FT4 3.0:1 TSH:FT3 8.5:1 | NR | NR | NR | I: -19.8% C: -9.5% |  |
| Bejjanki et al, USA (15) | NC | Decision tool | FT4: 15,597.6 $ TSH: 38,827.91 $ | FT4: 11,081.28 $ TSH: 24,519.44 $ | NR | NR | NR | FT4: -29%^b^ TSH: -37%^b^ | Cost savings from reducing duplicates. |
| Caldarelli et al, Italy (4) | NC | Decision tool | 71,134 € | 60,055 € + 1,964 € | NR | NR | NR | - 14.7%^b^ |  |
| Elrewini et al, Saudi Arabia (6) | NC | Education + Guidelines | 49,648 SAR | 30,000 SAR | NR | NR | NR | -39.6%^b^ | Cost spent on the unnecessary requests of TSH tests. |
| Hardwick et al, Canada (16) | NC | Guidelines + Change in Funding | 2,478 $ | 3,058 $ | NR | NR | NR | -34% | Change in total expected costs. |
| Janssens et al, Netherlands (17) | NC | Guidelines | FT4: 810 (8.29€/test) TSH: 2,092 (6.91€/test) | FT4: 306 TSH: 828 | NR | NR | NR | FT4: -62.2%^b^ TSH: -60.4%^b^ |  |
| Leung et al, USA (18) | NC | Education + Reminder | NR | -8176 $ | NR | NR | < 0.05^d^ | NR | Change of laboratory costs. |
| Stuart et al, Australia (19) | NC | Education + Guidelines + Feedback | 0.426 | 0.047 | NR | RR: [0.55, 1.23] | 0.001^d^ | -89% | Mean costs per patient. |
| **Pattern (Improvement of care)** | | | | | | | | | |
| Tomlin et al, New Zealand (14) | C | Education + Guidelines + Feedback | TSH I/C: 223.6/33.8 FT4 I/C: 144.2/29.1 FT3 I/C: 41.6/11.0 | TSH I/C: 215.2/32.0 FT4 I/C: 80.7/25.3 FT3 I/C: 26.6/9.4 | NR | RR: I/C: [-0.05,  -0.02]/  [-0.12, -0.01]  I/C: [-0.47,  -0.41]/  [-0.19, -0.07] I/C: [-0.41,  -0.31]/  [-0.23, -0.06] | I/C: < 0.01/0.11^d^ I/C: < 0.01^d^ I/C: < 0.01^d^ | I/C: -4%/-5% I/C: -44%/-13% I/C: -36%/-15% | Shift towards TSH. |
| Wong et al, USA (20) | C | Guidelines + Decision tool | Complete Thyroid Panel: 83% ^c^ Hyperthyroid Panel: 9% ^c^ Hypothyroid Panel: 7% ^c^ Thyroid Function Screen: 2%^c^ | Complete Thyroid Panel: 12% ^c^ Hyperthyroid Panel: 20% ^c^ Hypothyroid Panel: 37% ^c^ Thyroid Function Screen: 30% ^c^ | NR | NR | NR | Complete Thyroid Panel: -86%^b^ Hyperthyroid Panel: +122%^b^ Hypothyroid Panel: +429%^b^ Thyroid Function Screen: +1,400%^b^ | Sought to reduce complete thyroid panels. |
| Emerson and Emerson, USA (21) | NC | Decision tool | TSH: 5,300 ^c^ FT4: 750 ^c^ TT4: 1,700 ^c^ TT3: 800 ^c^ FTI/T3RU: 900 ^c^ TFT cascade: NA TSH + cascade: 5,300 ^c^ | TSH: 3,000 ^c^ FT4: 1450 ^c^ TT4: 200 ^c^ TT3: 500 ^c^ FTI/T3RU: 100 ^c^ TFT cascade: 1,750 ^c^ TSH + cascade: 4,750 ^c^ | NR | NR | NR | TSH: -43.4%^b^ FT4: +93.3%^b^ TT4: -88.2%^b^ TT3: -37.5%^b^ FTI/T3RU: -88.9%^b^ TFT cascade: +inf ^b^ TSH + cascade: -10.4%^b^ | Shift towards FT4 and thyroid cascade. |
| Hardwick et al, Canada (16) | NC | Guidelines + Change in Funding | Share of T3: 21.8% | Share of T3: 4.7% | NR | NR | NR | -78.4%^b^ | Sought to reduce proportion of T3 tests. |
| Larsson et al, Sweden (22) | NC | Education | TSH/TFTs: 0.124 T3/TSH: 0.129 T4/TSH: 0.333 | TSH/TFTs: 0.141 T3/TSH: 0.056 T4/TSH: 0.301 | TSH/TFTs: 0.017 T3/TSH: -0.073 T4/TSH: -0.032 | NR | TSH/TFTs: 0.0480^d^ T3/TSH: 0.0012^d^ T4/TSH: 0.1300 | TSH/TFTs: +13.7%^b^ T3/TSH: -56.6%^b^ T4/TSH: -9.6%^b^ | Shift towards TSH (primary care centres). |
| Larsson et al, Sweden (22) | NC | Education | TSH/TFTs: 0.120 T3/TSH: 0.152 T4/TSH: 0.334 | TSH/TFTs: 0.144 T3/TSH: 0.069 T4/TSH: 0.305 | NR | NR | NR | TSH/TFTs: +20.0%^b^ T3/TSH: -54.6%^b^ T4/TSH: -8.7%^b^ | Shift towards TSH (individual physicians). |
| Mindemark and Larsson, Sweden (follow up) (23) | NC | Education | T3/TSH 0.029 T4+FT4/TSH 0.273 | T3/TSH 0.022 T4+FT4/TSH 0.237 | TT3/TSH -0.009 TT4+FT4/TSH -0.036 | NR | ns ns | T3/TSH -31%^b^ T4+FT4/TSH -13%^b^ | Median of physicians; shift towards TSH. |
| Toubert et al, France (11) | NC | Guidelines + Reminders | Single TSH: 305  TSH + FT4: 319  TSH + FT3: 23  TSH + FT4 + FT3: 682 FT4 + FT3: 10 | Intervention / Post-intervention  Single TSH: 563 / 512 TSH + FT4: 313 / 333 TSH + FT3: 25 / 20 TSH + FT4 + FT3: 218 / 197 FT4 + FT3: 4 / 9 | NR | NR | NR | Intervention to Pre-intervention  +67.9%^b^ +4.4%^b^ -13.0%^b^ -71.1%^b^ -10.0%^b^ | Shift towards TSH |
| Van Walraven et al, Canada (24) | NC | Guidelines + Change in Funding + Decision tool | TSH: 1,000 ^c^ T4: 1,150 ^c^ | TSH/avoided total: 1,700 ^c^/2,200 T4: 50 ^c^/4,359 | NR | Avoided:  [-1638, 6,039] [-14, 23,430] | 0.03^d^ 0.02^d^ | TSH: - 12% T4: - 96% | Shift towards TSH |
| Vidal-Trecan et al, France (25) | NC | Education + Guidelines + Reminders + Decision tool | TFTs: 27,945 TSH (% TFTs): 12,336 (44%) T3: 339 (1%) FT3: 5,491 (20%) T4: 478 (2%) FT4: 9,301 (33%) | TFTs: 24,794 TSH (% TFTs): 12,526 (51%) T3: 371 (1%) FT3: 3,534 (14%) T4: 238 (1%) FT4: 8,125 (33%) | NR | NR | NR | Share of TSH: +15.9%^b^ | Shift towards TSH |
| **Test numbers or rates (Volume Reduction)** | | | | | | | | | |
| Baker et al, UK (26) | RCT | Guidelines + Feedback | NR | I: 13,2 C: 20,9 | -1,45 | RD: [-4.59, 1.68] | 0.35 | - 9.9%^b^ | Tests per 1,000 patients. |
| Thomas et al, UK Feedback (27) | RCT | Reminder + Feedback | 829 | 802 | NR | OR: [0.84, 0.97] | 0.005 | OR: 0.90 | Tests per 10,000 patients. |
| Thomas et al, UK Reminder (27) | RCT | Feedback | 961 | 891 | NR | OR: [0.83, 0.95] | 0.001 | OR: 0.82 | See above. |
| Thomas et al, UK Both (27) | RCT | Reminder | 891 | 800 | NR | NR | NR | NR | See above. |
| Bellodi et al, Italy (28) | C | Decision tool | NR | NR | NR | NR | NR | Delta: -17.24% Cento: -32,50% Ferrara: - 18,87% | Number of laboratory tests requested by wards. |
| Berwick and Coltin, USA PCF$ (12) | C | Feedback | 189 (72+72+45) | I (C): -12.1% (+1.7%) | NR | NR | NR | - 13.5%^b^ | Tests per 1,000 encounters per physician. |
| Berwick and Coltin, USA PCFY (12) | C | Feedback | 189 (72+72+45) | I (C): +34.0% (+1.7%) | NR | NR | NR | + 31.8%^b^  (negative effect) | See above. |
| Berwick and Coltin, USA TSE (12) | C | Education | 189 (72+72+45) | I (C): -15.9% (+1.7%) | NR | NR | NR | - 17.3%^b^ | See above. |
| Chami et al, Canada (29) | C | Decision tool | 34.38 Mio | 29.66 Mio | NR | NR | 0.09 | -14% | Number of thyroid tests. |
| Gama et al, UK (30) | C | Feedback | I: 0.17 C: 0.05 | I: 0.13 C: 0.06 | NR | NR | I: < 0.01^d^ C: > 0.05 | I: -21.9% C: +20.8% | Tests per outpatient visit. |
| Horn et al, USA (31) | C | Decision tool | I: 174.1 (+0.2%/month) C: 140.3 (+0.1%/month) | I: -0.5%/month C: +0.4%/month | I: -0.7% C: +0.3% | NR | 0.04 | NA | Monthly orders per 1,000 patients. |
| Schectman et al, USA (3) | C | Reminder + Feedback | 1.68 | 1.49 | NR | NR | < 0.0001^d^ | 11.3%^b^ | Number of TFTs per patient; Feedback and Non-Feedback group combined. |
| Tomlin et al, New Zealand (14) | C | Education + Guidelines + Feedback | TSH I/C: 223.6/33.8 FT4 I/C: 144.2/29.1 FT3 I/C: 41.6/11.0 | TSH I/C: 215.2/32.0 FT4 I/C: 80.7/25.3 FT3 I/C: 26.6/9.4 | NR | RR:  I/C: [-0.05,  -0.02]/  [-0.12, -0.01]  I/C: [-0.47,  -0.41]/  [-0.19, -0.07] I/C: [-0.41,  -0.31]/ [-0.23,  -0.06] | I/C: < 0.01/0.11^d^ I/C: < 0.01^d^ I/C: < 0.01^d^ | I/C: -4%/-5% I/C: -44%/-13% I/C: -36%/-15% | Tests per year per GP. |
| Wintemute et al, Canada (32) | C | Guidelines + Feedback | I: 1.74% C: 2.17% | I: 1.52%^b^ C: 2.13%^b^ | I: -0.22% C: -0.04% | RD:  [-0.33%,  -0.13%]  [-0.18%, 0.11%] | < 0.001^d^ 0.62 | I: -13.2% C: -1.8% |  |
| Wong et al, USA (20) | C | Guidelines + Decision tool | Average/month ^c^ TSH: 950 T3-RIA: 850 T3RU and T4-RIA: 1,000 | Average/month ^c^ TSH: 550 T3-RIA: 300 T3RU and T4-RIA: 1,000 | NR | NR | NR | TSH: -38% T3-RIA: -61% T3RU and T4-RIA ^c^: 0% | Tests per month. |
| Adlan et al, UK (33) | NC | Guidelines | 53,8% | 21,7% | NR | NR | < 0.001^d^ | - 59.7%^b^ | Proportion of admitted patients offered TFTs. |
| Bateman et al, Canada (34) | NC | Education + Feedback | 93,7% | 53,1% | NR | NR | NR | -43% | Proportion of admitted patients offered TFTs. |
| Bejjanki et al, USA (15) | NC | Decision tool | FT4: 0.1255 ^b^ TSH: 0.1251 ^b^ | FT4: 0.1132 ^b^ TSH 0.0937 ^b^ | FT4: - 1.23 TSH: -3.14 | RD FT4:  [–0.28, 2.74]  RD TSH:  [2.30, 3.97] | FT4: 0.11  TSH: 0.00^d^ | FT4: -9.8% TSH: -25.1% | Percentage change in the number of inpatient duplicate orders. |
| Bradshaw et al, USA (35) | NC | Decision tool | TSH: 63 FT4: 191 | TSH: 50 FT4: 133 | NR | NR | TSH: 0.062 FT4: 0.01^d^ | TSH: -20.6%^b^ FT4: -30% | Number of inappropriate TSH tests ordered; FT3 excluded due to low baseline numbers. |
| Caldarelli et al, Italy (4) | NC | Decision tool | TSH: 34,985 FT4: 29,283 FT3: 28,260 | TSH: 31,544 FT4: 24,548 FT3: 22,423 | NR | NR | NR | TSH: -9.8% FT4: -16.2% FT3: -20.6% | Number of thyroid tests. |
| Chu et al, Australia (36) | NC | Decision tool | 2.2 | 1.6 | NR | NR | 0.0001^d^ | - 27.3%^b^ | Number of tests ordered per 100 ED presentations. |
| Cipullo and Mostoufizadeh, USA (37) | NC | Guidelines | 0.006 | 0.005 | NR | NR | NR | - 16.7%^b^ | Tests / discharge. |
| Dalal et al, USA (38) | NC | Decision tool | FT3: 162 FT4: 1,164 TSH: 1,362 | FT3: 90 FT4: 664 TSH: 754 | NR | NR | < 0.0001^d^ | FT3: -45.2% FT4: -43.0% TSH: -44.6% | Number of tests of fT3 and fT4 orders per total TSH orders. |
| Dowling et al, USA (5) | NC | Education + Feedback | 0.0082 | 0.0056 | NR | NR | > 0.05 | -31.7%^b^ | Rates of ordering TSH tests per visit. |
| Emerson and Emerson, USA (21) | NC | Decision tool | TSH: 5,300 ^c^ FT4: 750 ^c^ TT4: 1,700 ^c^ TT3: 800 ^c^ FTI/T3RU: 900 ^c^ TFT cascade: NA TSH + cascade: 5,300 ^c^ | TSH: 3,000 ^c^ FT4: 1,450 ^c^ TT4: 200 ^c^ TT3: 500 ^c^ FTI/T3RU: 100 ^c^ TFT cascade: 1,750 ^c^ TSH + cascade: 4,750 ^c^ | NR | NR | NR | TSH: -43.4%^b^ FT4: +93.3%^b^ TT4: -88.2%^b^ TT3: -37.5%^b^ FTI/T3RU: -88.9%^b^ TFT cascade: +inf^b^ TSH + cascade: -10.4%^b^ | Test sets ordered (significance for total TFTs). |
| Feldkamp and Carey, USA (7) | NC | Decision tool | TSH: 832 T4: 667 T3RU: 234 | TSH: 982 T4: 216 T3RU: 159 | NR | NR | NR | TSH: +18.0%^b^ T4: -67.6%^b^ T3RU: -32.1%^b^ | Tests per 1,000 patients (T3 not reported). |
| Gilmour et al, Canada (39) | NC | Education + Decision tool | FT3: 39 FT4: 90 | FT3: 14 FT4: 59 | NR | NR | FT3:  < 0.017^d^ FT4:  < 0.017^d^ | FT3: 64%^b^ FT4: 34%^b^ | Median number of tests performed (FT3 and FT4; TSH used for initial appropriateness). |
| Grivell et al, Australia (40) | NC | Feedback | NR | NR | NR | NR | NR | +20%  (negative effect) | Tests per 1,000 patients. |
| Hardwick et al, Canada (16) | NC | Guidelines + Change in Funding | T3: 29,004 T4: 68,912 ETR: 35,183 | T3: 7,502 T4: 128,343 ETR: 23,703 | NR | NR | NR | T3: -74.1%^b^ T4: +86.2%^b^ ETR: -32.6%^b^ |  |
| Janssens et al, Netherlands (17) | NC | Guidelines | FT4: 810 (8.29€/test) TSH: 2,092 (6.91€/test) | FT4: 306 TSH: 828 | NR | NR | NR | FT4: -62.2%^b^ TSH: -60.4%^b^ |  |
| Krouss et al, USA (41) | NC | Decision tool | T3 (inpatient): 1.65 FT3 (inpatient): 1.47 T3 (outpatient): 1.55 FT3 (outpatient): 1.61 | T3 (inpatient): 0.68 FT3 (inpatient): 0.68 T3 (outpatient): 0.95 FT3 (outpatient): 0.75 | T3 (IP): -0.97 FT3 (IP): -0.79 T3 (OP): -0.60 FT3 (OP): -0.86 | NR | < 0.001^d^ | T3 (inpatient): -58.8%^b^ FT3 (inpatient): -53.7%^b^ T3 (outpatient): -38.7%^b^ FT3 (outpatient): -53.4%^b^ | Orders per 1,000 patient days (inpatient) / per 1,000 encounters (outpatient). |
| Leis et al, Canada (8) | NC | Decision tool | Order not indicated: 60.6% Test w/o indication: 73.3% Indication w/o test: 36.8% Overall appropriateness: 46.2% Overall test rate: 85.4% | Order not indicated: 20% Test w/o indication: 2.7% Indication w/o test: 81.8% Overall appropriateness: 61.9%^b^ Overall test rate: 36.4% | NR | NR | 0.01^d^ < 0.0001^d^ < 0.0001^d^ NR < 0.0001^d^ | -67.0%^b^ -96.3%^b^ +122%^b^ + 34.0%^b^ -57.4%^b^ | Patients with any TSH assay request / patients with physician-signed order. |
| MacPherson et al, Australia (42) | NC | Guidelines + Decision tool | Group I: 5.5% | Group II: 3.0% Group III: 3.5% | NR | NR | < 0.05^d^ | Group II: -45,5%^b^ Group III: -36.4%^b^ |  |
| Muris et al, Netherlands (43) | NC | Decision tool | 15.1 ^c^ | 14.8 ^c^ | NR | NR | < 0.05^d^ | -2,0%^b^ | Mean test ordering rate per 1,000 patients per month per general practice. |
| Notas et al, Greece (44) | NC | Decision tool | FT4/TSH: 92.9% FT4/patient: 16.63% FT3/TSH: 92.4% FT3/patient: 16.55% | FT4/TSH: 17.7% FT4/patient: 2.71% FT3/TSH: 10.8% FT3/patient: 1.66% | NR | NR | < 0.05^d^ NR < 0.05^d^ NR | -81% -83.7%^b^ -88% -90.0%^b^ | Number of TFTs per TSH ordered (FT4 and FT3) and per cent patients with TFT order, inpatients. |
| Notas et al, Greece (44) | NC | Decision tool | FT4/TSH: 96.4% FT3/TSH: 95.7% | FT4/TSH: 41% FT3/TSH: 31% | NR | NR | NR | -57.5%^b^ -67.6%^b^ | Number of TFTs per TSH ordered (FT4 and FT3), outpatients. |
| Rhyne and Gehlbach, USA (10) | NC | Education + Guidelines | 1.0 ^c^ | 0.97 ^c^ | NR | NR | < 0.05^d^ | -3%^b^ | TFTs per 100 patients. |
| Salinas et al, Spain (45) | NC | Decision tool | 0.42 | < 0.25 | NR | NR | NR | > 40.5%^b^ | Ratio of FT4 / TSH. |
| Sue et al, USA (46) | NC | Decision tool | 162.3 | NR | NR | NR | < 0.0001^d^ | > 44% | T3 laboratory tests / 10.000 patients per week. |
| Taher et al, Canada (47) | NC | Decision tool | FT4: 741.2 FT3: 364.8 | FT4 (Cycle 1/2): 574.6/477.8 FT3 (Cycle 1/2): 299.2/208.7 | NR | NR | NR | -24%/-39% -18%/-39% | Total number of fT4 and fT3 tests per month. |
| Toubert et al, France (11) | NC | Guidelines + Reminders | 0.32 | 0.08 | NR | NR | < 0.001^d^ | - 0.75%^b^ |  |
| Van Walraven et al, Canada (24) | NC | Guidelines + Change in Funding + Decision tool | TSH: 1329 FT4: 1011 FT3: 715 | TSH: 1062 FT4: 539 FT3: 226 | NR | NR | NR | -20.0%^b^ -46.7%^b^ -68.4%^b^ | Tests per 100,000 patients per month; comparison with expected values (T3RU not reported). |
| Vidal-Trecan et al, France (25) | NC | Education + Guidelines + Reminders + Decision tool | TSH: 1,000 ^c^ T4: 1,150 ^c^ | TSH/avoided total: 1,700 ^c^/2,200 T4: 50 ^c^/4,359 | NR | Avoided:  [-1,638, 6,039] [-14, 23,430] | 0.03^d^ 0.02^d^ | TSH: - 12% T4: - 96% |  |
| Willis and Datta, UK (48) | NC | Education + Guidelines | TFTs: 27,945 TSH (% TFTs): 12,336 (44%) T3: 339 (1%) FT3: 5,491 (20%) T4: 478 (2%) FT4: 9,301 (33%) | TFTs: 24,794 TSH (% TFTs): 12,526 (51%) T3: 371 (1%) FT3: 3,534 (14%) T4: 238 (1%) FT4: 8,125 (33%) | NR | NR | NR | TFTs: -11% | Tests per admission. |

Interventions sorted by outcome (red) and type of study. Deviations from standard outcome measure listed in last column: *notes on outcomes*.

^a^ Positive direction of effect unless stated otherwise.

^b^ Estimate based on authors calculation.

^c^ Numbers extracted from graph.

^d^ Statistically significant change.

**Abbreviations:** C = Controlled (Study); FT4 = Free Thyroxine; FT3 = Free Triiodothyronine; IP = Inpatient; MPC = Memorandum Pocket Card; NA = Not Applicable; NC = Uncontrolled (Study); NR = Not Reported; OP = Outpatient; TFT = Thyroid Function Test; TRF = Test Request Form; TSE = Test-Specific Education; TSH = Thyroid-Stimulating- Hormone (Thyrotropin); T3 = Triiodothyronine; T3RU = Triiodothyronine Resin Uptake; PCFY = Peer Comparison Feedback on Yield of Tests; PCF$ = Peer Comparison Feedback on Cost of Test Use; RCT = Randomised Controlled Trial; RD = Risk Difference (Difference in Means); RR = Relative Reduction.

Literature Cited

1. Daucourt V, Saillour-Glénisson F, Michel P, Jutand MA, Abouelfath A. A multicenter cluster randomized controlled trial of strategies to improve thyroid function testing. Med. Care 2003; 41(3):432–41.

2. Delvaux N, Piessens V, Burghgraeve T de, Mamouris P, Vaes B, Stichele RV et al. Clinical decision support improves the appropriateness of laboratory test ordering in primary care without increasing diagnostic error: the ELMO cluster randomized trial. Implementation science : IS 2020; 15(1):100.

3. Schectman JM, Elinsky EG, Pawlson LG. Effect of Education and Feedback on Thyroid Function Testing Strategies of Primary Care Clinicians. Arch. Intern. Med. 1991; 151(11):2163–6.

4. Caldarelli G, Troiano G, Rosadini D, Nante N. Adoption of TSH Reflex algorithm in an Italian clinical laboratory. Annali di igiene : medicina preventiva e di comunita 2017; 29(4):317–22.

5. Dowling PT, Alfonsi G, Brown MI, Culpepper L. An education program to reduce unnecessary laboratory tests by residents. J. Med. Educ. 1989; 64(7):410–2.

6. Elrewini AM, Zubair M, Afridi NK, Dildar MT, Javed H, Alwalah SM. To determine the effectiveness of different interventions to reduce unnecessary requests of serum thyroid stimulating hormone levels in a hospital. The Professional Medical Journal 2022; 29(05):686–92.

7. Feldkamp CS, Carey JL. An algorithmic approach to thyroid function testing in a managed care setting: 3-Year experience. AM. J. CLIN. PATHOL. 1996; 105(1):11–6.

8. Leis B, Frost A, Bryce R, Lyon AW, Coverett K. Altering standard admission order sets to promote clinical laboratory stewardship: A cohort quality improvement study. BMJ Qual. Saf. 2019; 28(10):846–52.

9. Nightingale PG, Peters M, Mutimer D, Neuberger JM. Effects of a computerised protocol management system on ordering of clinical tests. Quality in health care : QHC 1994; 3(1):23–8.

10. Rhyne RL, Gehlbach SH. Effects of an educational feedback strategy on physician utilization of thyroid function panels. The Journal of family practice 1979; 8(5):1003–7.

11. Toubert ME, Chevret S, Cassinat B, Schlageter MH, Beressi JP, Rain JD. From guidelines to hospital practice: Reducing inappropriate ordering of thyroid hormone and antibody tests. Eur. J. Endocrinol. 2000; 142(6):605–10.

12. Berwick DM, Coltin KL. Feedback reduces test use in a health maintenance organization. J. Am. Med. Assoc. 1986; 255(11):1450–4.

13. Tierney WM, McDonald CJ, Hui SL, Martin DK. Computer predictions of abnormal test results. Effects on outpatient testing. J. Am. Med. Assoc. 1988; 259(8):1194–8.

14. Tomlin A, Dovey S, Gauld R, Tilyard M. Better use of primary care laboratory services following interventions to 'market' clinical guidelines in New Zealand: A controlled before-and-after study. BMJ Qual. Saf. 2011; 20(3):282–90.

15. Bejjanki H, Mramba LK, Beal SG, Radhakrishnan N, Bishnoi R, Shah C et al. The role of a best practice alert in the electronic medical record in reducing repetitive lab tests. ClinicoEconomics and outcomes research : CEOR 2018; 10:611–8.

16.  Hardwick DF, Morrison JI, Tydeman J, Cassidy PA, Chase WH. Structuring complexity of testing: a process oriented approach to limiting unnecessary laboratory use. The American journal of medical technology 1982; 48 7:605–8.

17. Janssens PMW, Staring W, Winkelman K, Krist G. Active intervention in hospital test request panels pays. Clinical chemistry and laboratory medicine 2015; 53(5):731–42.

18. Leung E, Song S, Al-Abboud O, Shams S, English J, Naji W et al. An educational intervention to increase awareness reduces unnecessary laboratory testing in an internal medicine resident-run clinic. Journal of community hospital internal medicine perspectives 2017; 7(3):168–72.

19. Stuart PJ, Crooks S, Porton M. An interventional program for diagnostic testing in the emergency department. The Medical journal of Australia 2002; 177(3):131–4.

20. Wong ET, McCarron MM, Shaw ST. Ordering of Laboratory Tests in a Teaching Hospital: Can It Be Improved? JAMA 1983; 249(22):3076–80.

21. Emerson JF, Emerson SS. The impact of requisition design on laboratory utilization. AM. J. CLIN. PATHOL. 2001; 116(6):879–84.

22. Larsson A, Biom S, Wernroth ML, Hultén G, Tryding N. Effects of an education programme to change clinical laboratory testing habits in primary care. Scandinavian journal of primary health care 1999; 17(4):238–43.

23. Mindemark M, Larsson A. Long-term effects of an education programme on the optimal use of clinical chemistry testing in primary health care. Scandinavian journal of clinical and laboratory investigation 2009; 69(4):481–6.

24. van Walraven C, Goel V, Chan B. Effect of population-based interventions on laboratory utilization: A time-series analysis. J. Am. Med. Assoc. 1998; 280(23):2028–33.

25. Vidal-Trécan G, Toubert ME, Coste J, Paycha F, Durand-Zaleski I, Fulla Y et al. Reducing the number of T3 orders in the Paris hospital network: Towards better appropriatness of thyroid function test prescription. Ann. Endocrinol. 2003; 64(3):210–5.

26. Baker R, Smith JF, Lambert PC. Randomised controlled trial of the effectiveness of feedback in improving test ordering in general practice. Scand. J. Prim. Health Care 2003; 21(4):219–23.

27. Thomas RE, Croal BL, Ramsay C, Eccles M, Grimshaw J. Effect of enhanced feedback and brief educational reminder messages on laboratory test requesting in primary care: a cluster randomised trial. Lancet 2006; 367(9527):1990–6.

28. Bellodi E, Vagnoni E, Bonvento B, Lamma E. Economic and organizational impact of a clinical decision support system on laboratory test ordering. BMC medical informatics and decision making 2017; 17(1):179.

29. Chami N, Li Y, Weir S, Wright JG, Kantarevic J. Effect of Strict and Soft Policy Interventions on Laboratory Diagnostic Testing in Ontario, Canada: A Bayesian Structural Time Series Analysis. Health policy 2021; 125(2):254–60.

30. Gama R, Nightingale PG, Broughton PM, Peters M, Bradby GV, Berg J et al. Feedback of laboratory usage and cost data to clinicians: does it alter requesting behaviour? Annals of clinical biochemistry 1991; 28 (Pt 2):143–9.

31. Horn DM, Koplan KE, Senese MD, Orav EJ, Sequist TD. The impact of cost displays on primary care physician laboratory test ordering. Journal of general internal medicine 2014; 29(5):708–14.

32. Wintemute K, Greiver M, McIsaac W, Del Elisabeth Giudice M, Sullivan F, Aliarzadeh B et al. Choosing Wisely Canada campaign associated with less overuse of thyroid testing Retrospective parallel cohort study. Can. Fam. Phys. 2019; 65(11):E487-E496.

33. Adlan MA, Neel V, Lakra SS, Bondugulapati LNR, Premawardhana, L. D. K. E. Targeted thyroid testing in acute illness: Achieving success through audit. J. Endocrinol. Invest. 2011; 34(8 SUPPL.):e210-e213.

34. Bateman EA, Gob A, Chin-Yee I, MacKenzie HM. Reducing waste: A guidelines-based approach to reducing inappropriate Vitamin D and TSH testing in the inpatient rehabilitation setting. BMJ Open Qual. 2019; 8(4).

35. Bradshaw AB, Bonnecaze AK, Burns CA, Beardsley JR. Impact of an Interprofessional Collaborative Quality Improvement Initiative to Decrease Inappropriate Thyroid Function Testing. Hosp. Pharm. 2021; 56(5):481–5.

36. Chu KH, Wagholikar AS, Greenslade JH, O'Dwyer JA, Brown AF. Sustained reductions in emergency department laboratory test orders: Impact of a simple intervention. Postgrad. Med. J. 2013; 89(1056):566–71.

37. Cipullo JA, Mostoufizadeh M. Bringing order to test orders: one lab's story. CAP today 1996; 10(1):20–2.

38. Dalal S, Bhesania S, Silber S, Mehta P. Use of electronic clinical decision support and hard stops to decrease unnecessary thyroid function testing. BMJ Open Qual. 2017; 6(1):u223041. w8346.

39. Gilmour JA, Weisman A, Orlov S, Goldberg RJ, Goldberg A, Baranek H et al. Promoting resource stewardship: Reducing inappropriate free thyroid hormone testing. J. Eval. Clin. Pract. 2017; 23(3):670–5.

40. Grivell AR, Forgie HJ, Fraser CG, Berry MN. Effect of feedback to clinical staff of information on clinical biochemistry requesting patterns. Clinical chemistry 1981; 27(10):1717–20.

41. Krouss M, Israilov S, Alaiev D, Hupart K, Da Shin W, Mestari N et al. Free the T3: implementation of best practice advisory to reduce unnecessary orders. The American journal of medicine 2022; 135(12):1437–42.

42. MacPherson RD, Reeve SA, Stewart TV, Cunningham AES, Craven ML, Fox G et al. Effective strategy to guide pathology test ordering in surgical patients. ANZ journal of surgery 2005; 75(3):138–43.

43. Muris DMJ, Molenaers M, Nguyen T, Bergmans, P. W. M. P., van Acker BAC, Krekels MME et al. Effect of a price display intervention on laboratory test ordering behavior of general practitioners. BMC Fam. Pract. 2021; 22(1).

44. Notas G, Kampa M, Malliaraki N, Petrodaskalaki M, Papavasileiou S, Castanas E. Implementation of thyroid function tests algorithms by clinical laboratories: A four-year experience of good clinical and diagnostic practice in a tertiary hospital in Greece. Eur. J. Intern. Med. 2018; 54:81–6.

45. Salinas M, López-Garrigós M, Flores E, Leiva-Salinas M, Asencio A, Lugo J et al. Managing inappropriate requests of laboratory tests: From detection to monitoring. Am. J. Managed Care 2016; 22(9):e311-e316.

46. Sue LY, Kim JE, Oza H, Chong T, Woo HE, Cheng EM et al. Reducing Inappropriate Serum T3 Laboratory Test Ordering in Patients with Treated Hypothyroidism. Endocr. Pract. 2019; 25(12):1312–6.

47. Taher J, Beriault DR, Yip D, Tahir S, Hicks LK, Gilmour JA. Reducing free thyroid hormone testing through multiple Plan-Do-Study-Act cycles. Clin. Biochem. 2020; 81:41–6.

48. Willis EA, Datta BN. Effect of an educational intervention on requesting behaviour by a medical admission unit. Ann. Clin. Biochem. 2013; 50(2):166–8.
