# Supplementary material for: Effectiveness and mechanisms of interventions to reduce low-value thyroid function tests: a systematic review
Source: Syst Rev. 2026 Feb 25;15:111. doi: 10.1186/s13643-026-03119-8 (PMC13040701; doi:10.1186/s13643-026-03119-8)
Supplement: Supplementary file 12 — Additional file 12. Additional file 12 includes additional information on study characteristics, i.e. funding and reported conflict of interest. [file 13643_2026_3119_MOESM12_ESM.docx]

# **Additional Information on Funder and Conflict of Interest (COI)**

| **Study and country** | **Funder** | **Funder (category)** | **COI** | **Ethics approval^1^** |
| --- | --- | --- | --- | --- |
| Bateman et al, Canada (1) | None | No funding/specific grant. | None | Not required. |
| Bejjanki et al, USA (2) | NR | NR | None | Approved. |
| Bellodi et al, Italy (3) | “This work has been supported in the context of the project no. RF-2010-2310570, financed by the Italian Health Ministry under the “Ricerca Finalizzata 2010” call.” | Non-profit | None | Approved. |
| Bradshaw et al, USA (4) | None | No funding/specific grant. | None | Approved. |
| Caldarelli et al, Italy (5) | NR | NR | NR | NR |
| Chami et al, Canada (6) | NR | NR | None | NR |
| Dalal et al, USA (7) | NR | NR | None | Approved. |
| Delvaux et al, Belgium (8) | “The ELMO Study was funded through the Belgian Health Care Knowledge Centre (KCE) Trials Programme agreement KCE16011.” | Non-profit | None | Approved. |
| Elrewini et al, Saudi Arabia (9) | NR | NR | NR | Approved. |
| Gilmour et al, Canada (10) | None | No funding/specific grant. | None | Approved. |
| Janssens et al, Netherlands (11) | None | No funding/specific grant. | None | NR |
| Krouss et al, USA (12) | None | No funding/specific grant. | None | Approved. |
| Leis et al, Canada (13) | None | No funding/specific grant. | None | Approved. |
| Leung et al, USA (14) | None | No funding/specific grant. | None | Not required. |
| MacPherson et al, Australia (15) | NR | NR | NR | NR |
| Muris et al, Netherlands (16) | None | No funding/specific grant. | None | Approved. |
| Notas et al, Greece (17) | NR | NR | NR | NR |
| Salinas et al, Spain (18) | None | No funding/specific grant. | None | Approved. |
| Sue et al, USA (19) | NR | NR | None | NR |
| Taher et al, Canada (20) | None | No funding/specific grant. | None | Approved. |
| Wintemute et al, Canada (21) | “This study was supported by Health Quality Ontario through the ARTIC (Adopting Research to improve Care) program. Dr Greiver holds an investigator award from the Department of Family and Community Medicine at the University of Toronto and was supported by a research stipend from North York General Hospital.” | Non-profit | None | Approved. |
| Adlan et al, UK (22) | NR | NR | NR | NR |
| Baker et al, UK (23) | NR | NR | NR | NR |
| Berwick and Coltin, USA (24) | “This work was supported in part by grants from the National Fund for Medical Education and from the Harvard Community Health Plan Foundation, through the Institute for Health Research.” | Non-profit | NR | NR |
| Chu et al, Australia (25) | “The Queensland Emergency Medicine Research Foundation funded this study (QEMRF-PROJ-2009-016).” | Non-profit | None | Approved. |
| Cipullo and Mostoufizadeh, USA (26) | NR | NR | NR | NR |
| Daucourt et al, France (27) | NR | NR | NR | NR |
| Dowling et al, USA (28) | NR | NR | NR | NR |
| Emerson and Emerson, USA (29) | NR | NR | NR | NR |
| Feldkamp and Carey, USA (30) | NR | NR | NR | NR |
| Gama et al, UK (31) | “RG, PGN, PMGB, and MP acknowledge financial support from the DOH.” | Non-profit | NR | Approved. |
| Grivell et al, Australia (32) | “This work was supported in part by a generous grant from the Australian Commonwealth Department of Health.” | Non-profit | NR | NR |
| Hardwick et al, Canada (33) | “This study was supported in part from the Mr. and Mrs. P. A. Woodward Foundation, Vancouver, Canada, and the British Columbia Ministry of Health.” | Non-profit | NR | NR |
| Horn et al, USA (34) | None | No funding/specific grant. | None | Approved. |
| Larsson et al, Sweden (35) | “This work was supported by Socialstyrelsen and SIPRI, Stockholm, Sweden.” | Non-profit | NR | NR |
| Mindemark and Larsson, Sweden (follow up) (36) | NR | NR | None | NR |
| Nightingale et al, UK (37) | “PGN and Ml4 acknowledge financial support from the Department of Health and the EEC AIM OPENILABS Project.” | Unclear | NR | NR |
| Rhyne and Gehlbach, USA (38) | “This work was supported by a grant from the Robert Wood Johnson Foundation.” | Non-profit | NR | NR |
| Schectman et al, USA (39) | NR | NR | NR | NR |
| Stuart et al, Australia (40) | NR | NR | NR | Approved. |
| Thomas et al, UK (41) | “Charity funded. The Wellcome Trust UK (grant ref: 063790) - Health Services Research Training Fellowship The Health Services Research Unit is funded by the Chief Scientist Office of the Scottish Executive Department of Health. Ruth Thomas is funded by the Wellcome Trust (GR0673790AIA). Bernard Croal was supported by a grant from Grampian Endowments. Jeremy Grimshaw holds a Canada Research Chair in Health Knowledge Transfer and Uptake.” | Non-profit | None | Approved. |
| Tierney et al, USA (42) | “This study was supported in part by research grant HS-04996 from the National Center for Health Services Research, US Department of Health and Human Services.” | Non-profit | NR | NR |
| Tomlin et al, New Zealand (43) | “This research was completed as part of the authors’ employment in Best Practice Advocacy Centre (BPACnz) or its stakeholder organisations.” | Non-profit | None | Not required. |
| Toubert et al, France (44) | NR | NR | NR | NR |
| Van Walraven et al, Canada (45) | “Dr Goel is supported in part by a National Health Scholar Award from Health Canada, Ottawa, Ontario.” | Non-profit | NR | NR |
| Vidal-Trecan et al, France (46) | NR | NR | NR | NR |
| Willis and Datta, UK (47) | None | No funding/specific grant. | None | Not required. |
| Wong et al, USA (48) | NR | NR | NR | NR |

**Abbreviations:** COI = Conflict Of Interest, NR = Not Reported.

^1^ Approval includes waivers by the ethics committee. “Not required” if reported by authors.

Literature Cited

1. Bateman EA, Gob A, Chin-Yee I, MacKenzie HM. Reducing waste: A guidelines-based approach to reducing inappropriate Vitamin D and TSH testing in the inpatient rehabilitation setting. BMJ Open Qual. 2019; 8(4).

2. Bejjanki H, Mramba LK, Beal SG, Radhakrishnan N, Bishnoi R, Shah C et al. The role of a best practice alert in the electronic medical record in reducing repetitive lab tests. ClinicoEconomics and outcomes research : CEOR 2018; 10:611–8.

3. Bellodi E, Vagnoni E, Bonvento B, Lamma E. Economic and organizational impact of a clinical decision support system on laboratory test ordering. BMC medical informatics and decision making 2017 [cited 20171222//]; 17(1):179.

4. Bradshaw AB, Bonnecaze AK, Burns CA, Beardsley JR. Impact of an Interprofessional Collaborative Quality Improvement Initiative to Decrease Inappropriate Thyroid Function Testing. Hosp. Pharm. 2021; 56(5):481–5.

5. Caldarelli G, Troiano G, Rosadini D, Nante N. Adoption of TSH Reflex algorithm in an Italian clinical laboratory. Annali di igiene : medicina preventiva e di comunita 2017; 29(4):317–22.

6. Chami N, Li Y, Weir S, Wright JG, Kantarevic J. Effect of Strict and Soft Policy Interventions on Laboratory Diagnostic Testing in Ontario, Canada: A Bayesian Structural Time Series Analysis. Health policy (Amsterdam, Netherlands) 2021 [cited 20201024//]; 125(2):254–60.

7. Dalal S, Bhesania S, Silber S, Mehta P. Use of electronic clinical decision support and hard stops to decrease unnecessary thyroid function testing. BMJ Open Qual. 2017; 6(1):u223041. w8346.

8. Delvaux N, Piessens V, Burghgraeve T de, Mamouris P, Vaes B, Stichele RV et al. Clinical decision support improves the appropriateness of laboratory test ordering in primary care without increasing diagnostic error: the ELMO cluster randomized trial. Implementation science : IS 2020 [cited 20201104//]; 15(1):100.

9. Elrewini AM, Zubair M, Afridi NK, Dildar MT, Javed H, Alwalah SM. To determine the effectiveness of different interventions to reduce unnecessary requests of serum thyroid stimulating hormone levels in a hospital. The Professional Medical Journal 2022; 29(05):686–92.

10. Gilmour JA, Weisman A, Orlov S, Goldberg RJ, Goldberg A, Baranek H et al. Promoting resource stewardship: Reducing inappropriate free thyroid hormone testing. J. Eval. Clin. Pract. 2017; 23(3):670–5.

11. Janssens PMW, Staring W, Winkelman K, Krist G. Active intervention in hospital test request panels pays. Clinical chemistry and laboratory medicine 2015; 53(5):731–42.

12. Krouss M, Israilov S, Alaiev D, Hupart K, Da Shin W, Mestari N et al. Free the T3: implementation of best practice advisory to reduce unnecessary orders. The American journal of medicine 2022; 135(12):1437–42.

13. Leis B, Frost A, Bryce R, Lyon AW, Coverett K. Altering standard admission order sets to promote clinical laboratory stewardship: A cohort quality improvement study. BMJ Qual. Saf. 2019; 28(10):846–52.

14. Leung E, Song S, Al-Abboud O, Shams S, English J, Naji W et al. An educational intervention to increase awareness reduces unnecessary laboratory testing in an internal medicine resident-run clinic. Journal of community hospital internal medicine perspectives 2017 [cited 20170713//]; 7(3):168–72.

15. MacPherson RD, Reeve SA, Stewart TV, Cunningham AES, Craven ML, Fox G et al. Effective strategy to guide pathology test ordering in surgical patients. ANZ journal of surgery 2005; 75(3):138–43.

16. Muris DMJ, Molenaers M, Nguyen T, Bergmans, P. W. M. P., van Acker BAC, Krekels MME et al. Effect of a price display intervention on laboratory test ordering behavior of general practitioners. BMC Fam. Pract. 2021; 22(1).

17. Notas G, Kampa M, Malliaraki N, Petrodaskalaki M, Papavasileiou S, Castanas E. Implementation of thyroid function tests algorithms by clinical laboratories: A four-year experience of good clinical and diagnostic practice in a tertiary hospital in Greece. Eur. J. Intern. Med. 2018; 54:81–6.

18. Salinas M, López-Garrigós M, Flores E, Leiva-Salinas M, Asencio A, Lugo J et al. Managing inappropriate requests of laboratory tests: From detection to monitoring. Am. J. Managed Care 2016; 22(9):e311-e316.

19. Sue LY, Kim JE, Oza H, Chong T, Woo HE, Cheng EM et al. Reducing Inappropriate Serum T3 Laboratory Test Ordering in Patients with Treated Hypothyroidism. Endocr. Pract. 2019; 25(12):1312–6.

20. Taher J, Beriault DR, Yip D, Tahir S, Hicks LK, Gilmour JA. Reducing free thyroid hormone testing through multiple Plan-Do-Study-Act cycles. Clin. Biochem. 2020; 81:41–6.

21. Wintemute K, Greiver M, McIsaac W, Del Elisabeth Giudice M, Sullivan F, Aliarzadeh B et al. Choosing Wisely Canada campaign associated with less overuse of thyroid testing Retrospective parallel cohort study. Can. Fam. Phys. 2019; 65(11):E487-E496.

22. Adlan MA, Neel V, Lakra SS, Bondugulapati LNR, Premawardhana, L. D. K. E. Targeted thyroid testing in acute illness: Achieving success through audit. J. Endocrinol. Invest. 2011; 34(8 SUPPL.):e210-e213.

23. Baker R, Smith JF, Lambert PC. Randomised controlled trial of the effectiveness of feedback in improving test ordering in general practice. Scand. J. Prim. Health Care 2003; 21(4):219–23.

24. Berwick DM, Coltin KL. Feedback reduces test use in a health maintenance organization. J. Am. Med. Assoc. 1986; 255(11):1450–4.

25. Chu KH, Wagholikar AS, Greenslade JH, O'Dwyer JA, Brown AF. Sustained reductions in emergency department laboratory test orders: Impact of a simple intervention. Postgrad. Med. J. 2013; 89(1056):566–71.

26. Cipullo JA, Mostoufizadeh M. Bringing order to test orders: one lab's story. CAP today 1996; 10(1):20–2.

27. Daucourt V, Saillour-Glénisson F, Michel P, Jutand MA, Abouelfath A. A multicenter cluster randomized controlled trial of strategies to improve thyroid function testing. Med. Care 2003; 41(3):432–41.

28. Dowling PT, Alfonsi G, Brown MI, Culpepper L. An education program to reduce unnecessary laboratory tests by residents. J. Med. Educ. 1989; 64(7):410–2.

29. Emerson JF, Emerson SS. The impact of requisition design on laboratory utilization. AM. J. CLIN. PATHOL. 2001; 116(6):879–84.

30. Feldkamp CS, Carey JL. An algorithmic approach to thyroid function testing in a managed care setting: 3-Year experience. AM. J. CLIN. PATHOL. 1996; 105(1):11–6.

31. Gama R, Nightingale PG, Broughton PM, Peters M, Bradby GV, Berg J et al. Feedback of laboratory usage and cost data to clinicians: does it alter requesting behaviour? Annals of clinical biochemistry 1991; 28 (Pt 2):143–9.

32. Grivell AR, Forgie HJ, Fraser CG, Berry MN. Effect of feedback to clinical staff of information on clinical biochemistry requesting patterns. Clinical chemistry 1981; 27(10):1717–20.

33.  Hardwick DF, Morrison JI, Tydeman J, Cassidy PA, Chase WH. Structuring complexity of testing: a process oriented approach to limiting unnecessary laboratory use. The American journal of medical technology 1982; 48 7:605–8.

34. Horn DM, Koplan KE, Senese MD, Orav EJ, Sequist TD. The impact of cost displays on primary care physician laboratory test ordering. Journal of general internal medicine 2014 [cited 20131121//]; 29(5):708–14.

35. Larsson A, Biom S, Wernroth ML, Hultén G, Tryding N. Effects of an education programme to change clinical laboratory testing habits in primary care. Scandinavian journal of primary health care 1999; 17(4):238–43.

36. Mindemark M, Larsson A. Long-term effects of an education programme on the optimal use of clinical chemistry testing in primary health care. Scandinavian journal of clinical and laboratory investigation 2009; 69(4):481–6.

37. Nightingale PG, Peters M, Mutimer D, Neuberger JM. Effects of a computerised protocol management system on ordering of clinical tests. Quality in health care : QHC 1994; 3(1):23–8.

38. Rhyne RL, Gehlbach SH. Effects of an educational feedback strategy on physician utilization of thyroid function panels. The Journal of family practice 1979; 8(5):1003–7.

39. Schectman JM, Elinsky EG, Pawlson LG. Effect of Education and Feedback on Thyroid Function Testing Strategies of Primary Care Clinicians. Arch. Intern. Med. 1991; 151(11):2163–6.

40. Stuart PJ, Crooks S, Porton M. An interventional program for diagnostic testing in the emergency department. The Medical journal of Australia 2002; 177(3):131–4.

41. Thomas RE, Croal BL, Ramsay C, Eccles M, Grimshaw J. Effect of enhanced feedback and brief educational reminder messages on laboratory test requesting in primary care: a cluster randomised trial. Lancet (London, England) 2006; 367(9527):1990–6.

42. Tierney WM, McDonald CJ, Hui SL, Martin DK. Computer predictions of abnormal test results. Effects on outpatient testing. J. Am. Med. Assoc. 1988; 259(8):1194–8.

43. Tomlin A, Dovey S, Gauld R, Tilyard M. Better use of primary care laboratory services following interventions to 'market' clinical guidelines in New Zealand: A controlled before-and-after study. BMJ Qual. Saf. 2011; 20(3):282–90.

44. Toubert ME, Chevret S, Cassinat B, Schlageter MH, Beressi JP, Rain JD. From guidelines to hospital practice: Reducing inappropriate ordering of thyroid hormone and antibody tests. Eur. J. Endocrinol. 2000; 142(6):605–10.

45. van Walraven C, Goel V, Chan B. Effect of population-based interventions on laboratory utilization: A time-series analysis. J. Am. Med. Assoc. 1998; 280(23):2028–33.

46. Vidal-Trécan G, Toubert ME, Coste J, Paycha F, Durand-Zaleski I, Fulla Y et al. Reducing the number of T3 orders in the Paris hospital network: Towards better appropriatness of thyroid function test prescription. Ann. Endocrinol. 2003; 64(3):210–5.

47. Willis EA, Datta BN. Effect of an educational intervention on requesting behaviour by a medical admission unit. Ann. Clin. Biochem. 2013; 50(2):166–8.

48. Wong ET, McCarron MM, Shaw ST. Ordering of Laboratory Tests in a Teaching Hospital: Can It Be Improved? JAMA 1983; 249(22):3076–80.
